# Design, Synthesis, Molecular Docking, Dynamics Simulation, and Biological Evaluation of Novel Thiazolidinedione Derivatives Against Breast Cancer with Apoptosis-Inducing Activity

**DOI:** 10.1021/acsomega.5c07713

**Published:** 2025-12-23

**Authors:** Pouria Zarrin, Sarah Gado, Ali Farhang Boroujeni, Ibrahim Gadaşlı, Fatma Zeynep Bozkurt, Demet Cansaran-Duman, Pelin Mutlu, Zeynep Ates-Alagoz

**Affiliations:** † 37504 Ankara University, Faculty of Pharmacy, Department of Pharmaceutical Chemistry, Ankara 06560, Turkey; ‡ Ankara University, Graduate School of Health Sciences, Ankara 06110, Turkey; § Ankara University, Biotechnology Institute, Department of Biotechnology, Ankara 06135, Turkey

## Abstract

Breast cancer remains one of the leading causes of cancer-related deaths among women worldwide. The chemotherapeutic drugs used in treatment often have serious side effects. In light of their anticancer potential, thiazolidinedione (TZD) derivatives are considered to be promising candidates for the development of novel antitumor agents. The objective of this study is to synthesize and produce two sets of TZD derivatives by combining the structural features of microtubule-targeting drugs used in breast cancer treatment, and to determine their molecular docking, molecular dynamics simulations, ADMET profile, antiproliferative, and apoptotic effect potential. In the present study, PZ-11 was determined by xCELLigence analysis to have the highest antiproliferative potential among all compounds tested on MCF-7 breast cancer cells. The cytotoxic activity of the synthesized compounds was evaluated against MCF-7 breast cancer cells, revealing IC_50_ values of 29.44 μM for PZ-9 and 17.35 μM for PZ-11, compared to 6.45 μM for the reference drug vincristine. Analysis of the gene expression of the PZ-11 compound, which has a stronger cytotoxic effect potential, showed that PZ-11 significantly downregulates *AIFM1, BAG3*, and *BIRC3*, while upregulating pro-apoptotic genes such as *BAD, HRK, CASP10*, and *CASP14*. PZ-11’s binding affinities were screened using a molecular docking workflow via KNIME. The robust and persistent interactions between PZ-11 and AIF were substantiated by molecular dynamics simulation. It is demonstrated by ADMET predictions that PZ compounds possess suitable pharmacokinetic properties. PZ-11 is a promising TZD-based anticancer drug candidate against breast cancer cells, as determined by computational and experimental analysis. However, further validation is required through *in vivo* analysis to support these findings.

## Graphical abstract

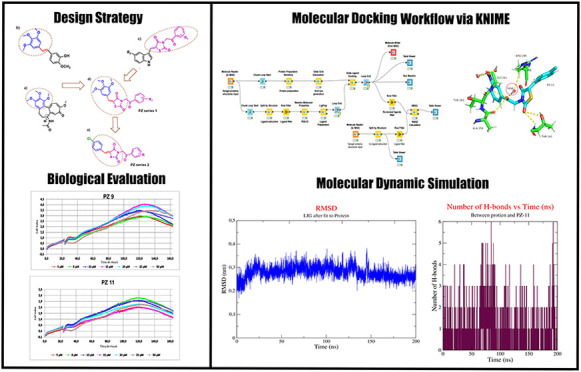

## 1 Introduction

Cancer continues to have a considerable impact on global health, with GLOBOCAN’s 2022 estimates indicating nearly 20 million new cases and approximately 9.7 million fatalities.^1^ Despite significant advancements in reducing breast cancer mortality in recent decades, breast cancer remains the second leading cause of cancer-related deaths in women. The five-year relative survival rate exhibits a significant decrease, from over 99% for localized disease to a mere 32% for distant-stage disease. Concurrently, current therapeutic interventions continue to demonstrate decreased effects for advanced and triple-negative breast cancers.^2^ Despite advances in early detection and development of targeted therapies, resistance to conventional chemotherapeutic agents and adverse side effects commonly restrict the efficacy of therapy. Consequently, the search for innovative therapeutic agents characterized by increased selectivity, lower toxicity, and the potential to induce apoptosis in cancer cells remains an important focus in breast cancer research.^3^

Thiazolidinedione (TZD) compounds, traditionally used as insulin inducers for type 2 diabetes mellitus, have received significant interest in oncology due to their various biological effects, including pro-apoptotic, anti-inflammatory, and antiproliferative effects. The anticancer effects of these drugs are demonstrated through a variety of mechanisms, including cell cycle arrest, the induction of intrinsic apoptotic pathways, and the regulation of peroxisome proliferator-activated receptor γ (PPAR-γ). The TZD ring has been identified as a potentially significant scaffold for the development of new anticancer drugs, with recent studies indicating that structural modifications can significantly increase its anticancer potential^4,5^

In this study, Thiazolidinedione (TZD) was selected as the primary scaffold due to its demonstrated properties of broad-spectrum DNA toxicity with selective effects on DNA replication and transcription. The indole-thiazolidinedione derivatives synthesized in our previous study^6^ exhibited a significant cytotoxic effect on MCF-7 breast cancer cells, through apoptosis and cell cycle arrest. Consequently, the integration of hybrid derivatives of TZD within cancer therapy is derived from the compound’s capacity to exhibit diverse impacts on multiple cancer pathways, in conjunction with the scaffold’s inherent ability to seamlessly accommodate various pharmacophores. The combination of the TZD scaffold with additional biologically active moieties has the potential to produce novel, more effective, and reduced-toxicity compounds. The majority of synthesized TZD-hybrids are classified as conjugated hybrids, wherein an additional scaffold possessing anticancer properties is connected to the TZD scaffold at designated positions (C5 or the acidic -NH atom of the TZD ring). The reactivity characteristics of the TZD ring have been shown to form structurally diverse hybrids via Knoevenagel condensation and N-substitution, thereby facilitating the synthesis of novel compounds capable of effectively modulating cancer pathways.^7^

In the present study, colchicine, vincristine, and combretastatin A-4 were chosen as reference compounds due to their comprehensively defined mechanisms as microtubule-targeting agents and their proven anticancer effects, particularly through induction of cell cycle arrest and apoptosis. It is notable that these three molecules are mechanistically relevant and pharmacologically potent benchmarks, given that several TZD derivatives are thought to disrupt comparable cellular functions, such as microtubule integrity and apoptotic signaling.^8^

The selection of the moiety to be linked to the TZD ring was determined by the chemical structure of colchicine and combretastatin A4 (Figure 1). In structure–activity relationship (SAR) studies of colchicine, which has been determined to have a therapeutic effect on breast cancer due to its potential to inhibit cell proliferation and induce apoptosis, the 3,4,5-trimethoxyphenyl group is essential for its tubulin destabilizing function, which creates its cytotoxic potential on cancer cells.^9,10^ In addition to colchicine, Combretastatin A4 (CA-4) (Figure 1) is a chemotherapeutic agent with promising inhibitory potential against various cancer cells, including breast cancer, and phase III studies are in progress for CA-4 in the form of water-soluble drugs for different solid tumors. Structure–activity studies indicate that the 3,4,5-trimethoxyphenyl and olefinic bonds in CA-4 and its analogues are important for exhibiting anticancer effects.^11–13^

**Figure 1:**
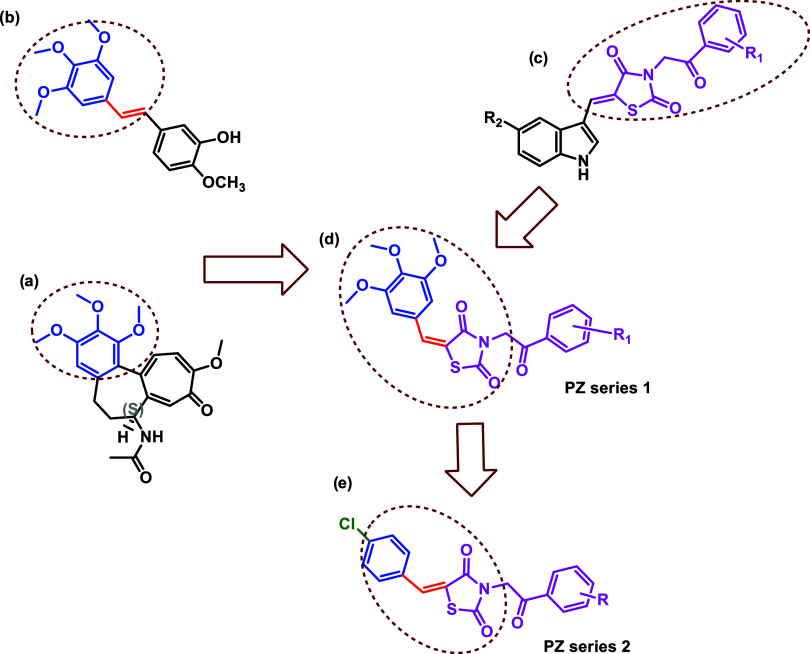
(a) Colchicine. (b) Combretastatin A4 (CA-4). (c) Our previously synthesized compounds. (d, e) Target molecules planned to be synthesized.

This study aims to design, synthesize, and evaluate the potential of two series of thiazolidinedione derivatives as CA-4 analogues that preserve the trimethoxyphenyl structure and conjugated double bond in CA-4, and to determine their potential as drug candidates for breast cancer. Each derivative is constructed around the thiazolidinedione core, with the first series featuring a trimethoxybenzylidene moiety at the fifth position and the second series featuring a para-chlorobenzylidene moiety linked via an unsaturated aliphatic linker. The addition of an acetophenone group to the nitrogen atom of the thiazolidine ring increases the chemical complexity of the derivatives. For this purpose, within the scope of this study, it is possible to analyze variations in biological activity associated with specific derivatives, as each acetophenone unit possesses a different aromatic substitution property. Furthermore, MCF-7 breast cancer cells, which are estrogen receptor-positive (ER^+^) and represent a well-characterized luminal A subtype of breast cancer, were used to evaluate the cytotoxic and apoptosis-inducing potential of the newly synthesized compounds as candidates against chemotherapeutic agents with similar mechanisms. The study compared the potential effects of TZD compounds on breast cancer with those of vincristine, a standard microtubule-targeting agent. This revealed the potential of structural modifications to the TZD skeleton to enhance anticancer potential, particularly through mechanisms related to mitochondrial apoptosis or microtubule destabilization. This study demonstrated the significant impact of structural optimization on biological effects and provided notable support for the potential of new thiazolidinedione-based compounds as chemotherapy candidates for breast cancer treatment.

## 2 Experimental Section

### 2.1 Chemicals and Reagents

All chemical substances utilized in this study, including both solvents and reagents, were purchased from Sigma-Aldrich and BLDpharm. These materials were employed in the experimental procedures as received without undergoing any additional purification processes. Furthermore, all synthetic reactions and procedures were conducted under standard laboratory conditions, specifically at room temperature or at the temperatures explicitly stated for each individual step.

### 2.2 Physical Measurements

The progression of chemical reactions was routinely monitored using thin-layer chromatography (TLC), which was performed on commercially available silica gel plates (Kieselgel 60 F254, Merck, Germany). Visualization of the TLC spots was achieved by exposure to ultraviolet light at a wavelength of 254 nm and 366 nm. For molecular weight determination and compound identification, mass spectrometric analyses were conducted by using a Waters ZQ Micromass LC–MS spectrometer (Waters Co., Milford, MA) equipped with an electrospray ionization (ESI) source. Nuclear magnetic resonance (NMR) spectroscopy was employed for structural characterization, with spectra recorded in deuterated dimethyl sulfoxide (DMSO-*d*_6_) or deuterated chloroform (CDCl_3_) as solvents. A Varian Mercury FT-NMR spectrometer (Varian, Inc., Palo Alto, California) was used for this purpose, employing tetramethylsilane (TMS) as the internal chemical shift reference standard. Proton NMR (^1^H NMR) spectra were obtained at a frequency of 500 MHz, while carbon-13 NMR (^13^C NMR) spectra were recorded at 125 MHz. Reported values include coupling constants (*J*) in hertz (Hz) and chemical shifts (δ) in parts per million (ppm). Additionally, the melting points of the synthesized compounds were determined by using a Buchi B540 melting point apparatus, with samples placed in open capillary tubes. No corrections were applied to the recorded melting point values.

### 2.3 Chemical Synthesis

The synthesis pathway of the final compounds was shown in Figure 2. First, thiazolidine-2,4-dione was prepared by reacting chloroacetic acid (10 g) with thiourea (8.55 g) in 10 mL of water under reflux in a sealed flask. The reaction was monitored by TLC. When complete, the solid was filtered, washed with water, dissolved in methanol, and recrystallized. For arrival at the compounds, thiazolidine-2,4-dione (0.01 mol) was dissolved in 10 mL of methanol. Potassium hydroxide (0.01 mol), dissolved in 6.5 mL of methanol, was added dropwise. After stirring for 10 min, the required acetophenone (0.01 mol, substituted or unsubstituted) was added. The mixture was stirred for another 5 min, then refluxed for 40 h. After the reaction finished, the mixture was filtered and rinsed with methanol. The resulting solid was recrystallized from ethanol to give the intermediate compounds^6,14^

**Figure 2:**
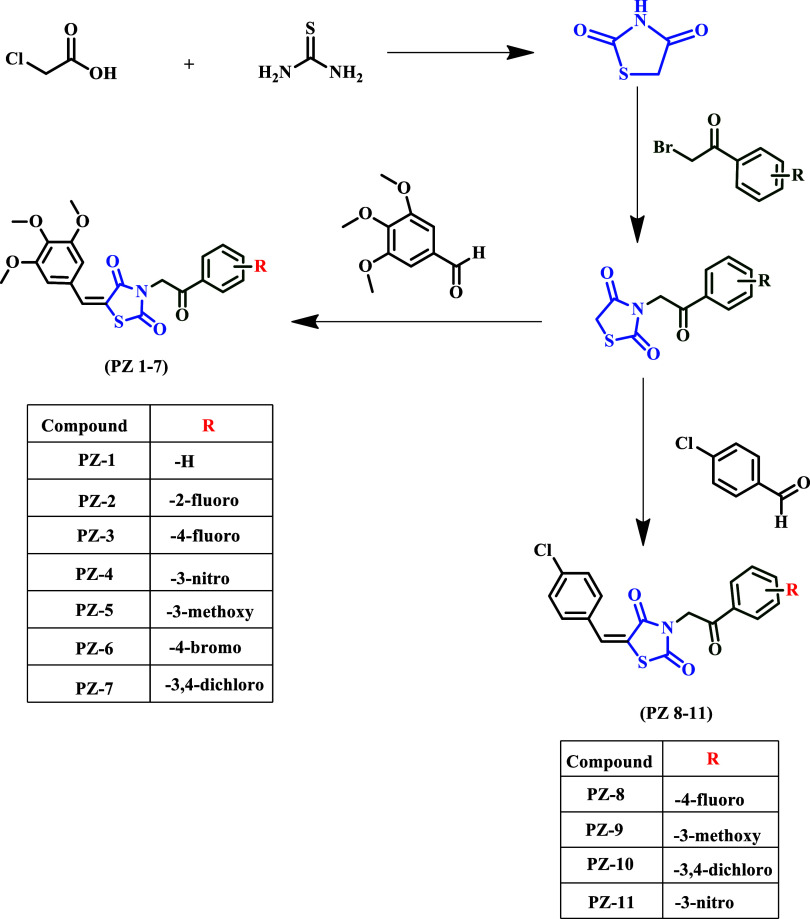
All steps were involved in the synthesis of the compounds.

#### 2.3.1 General Method for the Synthesis of 3-(2-oxo-2-phenylethyl)-5-(3,4,5-trimethoxybenzylidene)­thiazolidine-2,4-dione Derivatives **(PZ1-PZ7**) & 5-(4-chlorobenzylidene)-3-(2-oxo-2-phenylethyl)­thiazolidine-2,4-dione Derivatives **(PZ8-PZ11)**

In the final step, a solution of trimethoxybenzaldehyde (1.2 mmol) or 4-chlorobenzaldehyde in glacial acetic acid (4 mL) was prepared, and 3-(substituted phenacyl)-2,4-thiazolidinedione (1 mmol) along with sodium acetate (0.4 g) was added. The reaction mixture was refluxed at 170–180 °C for 40 h. The resulting precipitate was filtered, washed with water and methanol, and purified using column chromatography (cc) with silica gel 60 (230–400 mesh ASTM). A mixture of *n*-hexane and chloroform (4:1) or *n*-hexane and ethyl acetate (3:1) was used as the eluting solvent to purify the final compounds.^6,12^

#### 2.3.2 (E/Z)-3-(2-oxo-2-phenylethyl)-5-(3,4,5-trimethoxybenzylidene)­thiazolidine-2,4-dione **(1)**

Compound **PZ-1** was prepared according to the general method starting from 3-(2-oxoethyl)­thiazolidine-2,4-dione (1 mmol, 0.235 g) and 3,4,5-trimethoxybenzaldehyde (1.2 mmol, 0.235 g). The residue was purified by cc using a mixture of chloroform/*n*-hexane (4:1) as the eluent. Mp 199–200 °C, (70% yield). MS (ES^+^) (%): 436.63 ([M + Na]^+^, 40%), 849.9 ([2 M + Na]^+^, 100%). ^1^H NMR (500 MHz, DMSO-d6): δ ppm 3.75 (s, 3H), 3.86 (s, 6H), 5.34 (s, 2H), 7.01­(s, 2H), 7.60–7.63 (t, *J* = 15.55 Hz, 2H), 7.74–7.77 (t, *J* = 14.85 Hz, 1H), 7.97­(s, 1H), 8.09–8.10 (t, *J* = 8.4 Hz, 2H). ^13^C NMR (125 MHz, DMSO-d6): δ ppm 48.37, 56.54, 60.71, 108.26, 120.24, 128.79, 129.53, 134.25, 134.63, 134.96, 140.25, 153.74, 165.65, 167.48, 191.80.

#### 2.3.3 (E/Z)-3-(2-(2-fluorophenyl)-2-oxoethyl)-5-(3,4,5-trimethoxybenzylidene)­thiazolidine-2,4-dione **(2)**

Compound **PZ-2** was prepared according to the general method starting from 3-(2-(2-fluorophenyl)-2-oxoethyl)­thiazolidine-2,4-dione (1 mmol, 0.253 g) and 3,4,5-trimethoxybenzaldehyde (1.2 mmol, 0.235 g). The residue was purified by cc using the mixture of chloroform/*n*-hexane (4:1) as the eluent. Mp 172–173 °C, (62% yield). MS (ES^+^) (%): 454.6 ([M + Na]^+^, 100%). ^1^H NMR (500 MHz, CDCl_3_): δ ppm 3.94 (d, *J* = 2 Hz, 9H), 5.13 (d, *J* = 3.75 Hz, 2H), 6.79 (s, 2H), 7.22–7.33­(m, 2H), 7.64–7.65­(m, 1H), 7.89 (s, 1H), 8.00–8.04 (td, *J* = 7.5, 1.65 Hz, 1H). ^13^C NMR (125 MHz, CDCl_3_): δ ppm 50.9, 51.03, 56.23, 61.07, 107.51, 116.78 (d, *J* = 23.4 Hz), 120.35, 122.29, 122.40, 124.9 (d, *J* = 2.7 Hz), 128.63, 131.10 (d, *J* = 2.5 Hz), 134.54, 136.17 (d, *J* = 9.2 Hz), 140.35, 153.61, 161.68, 167.70 (d, *J* = 224 Hz), 167.60, 188.03 (d, *J* = 5 Hz).

#### 2.3.4 (E/Z)-3-(2-(4-fluorophenyl)-2-oxoethyl)-5-(3,4,5-trimethoxybenzylidene)­thiazolidine-2,4-dione **(3)**

Compound **PZ-3** was prepared according to the general method starting from 3-(2-(4-fluorophenyl)-2-oxoethyl)­thiazolidine-2,4-dione (1 mmol, 0.253 g) and 3,4,5-trimethoxybenzaldehyde (1.2 mmol, 0.235 g). The residue was purified by cc using the mixture of chloroform/*n*-hexane (4:1) as the eluent. Mp 164–165 °C, (65% yield). MS (ES^+^) (%): 454.6 ([M + Na]^+^, 100%). ^1^H NMR (500 MHz, CDCl_3_): δ ppm 3.94 (d, *J* = 3.5 Hz, 9H), 5.16 (s, 2H), 6.78 (s, 2H), 7.22 (t, *J* = 8.5 Hz, 2H), 7.88­(s, 1H), 8.03–8.06 (m, 2H). ^13^C NMR (125 MHz, CDCl_3_): δ ppm 47.26, 56.24, 61.08, 107.54, 116.27 (d, *J* = 21.9 Hz), 120.21, 128.56, 130.65 (d, *J* = 2.99 Hz), 130.67, 130.92 (d, *J* = 9.5 Hz), 134.72, 140.43, 153.62, 165.35, 165.78, 167.39, 167.59, 188.25.

#### 2.3.5 (E/Z)-3-(2-(3-nitrophenyl)-2-oxoethyl)-5-(3,4,5-trimethoxybenzylidene)­thiazolidine-2,4-dione **(4)**

Compound **PZ-4** was prepared according to the general method starting from 3-(2-(3-nitrophenyl)-2-oxoethyl)­thiazolidine-2,4-dione (1 mmol, 0.280 g) and 3,4,5-trimethoxybenzaldehyde (1.2 mmol, 0.235 g). The residue was purified by cc using the mixture of chloroform/*n*-hexane (4:1) as the eluent. Mp 160–161 °C, (51% yield). MS (ESI^+^) (%): 481.33 ([M + Na]^+^, 40%), 167.18 (C_9_H_11_O_3_^+^; trimethoxybenzyl cation, 100%). ^1^H NMR (500 MHz, CDCl_3_): δ ppm 3.94 (d, *J* = 2 Hz, 9H), 5.24 (s, 2H), 6.79 (s, 2H), 7.80 (t, *J* = 8 Hz, 1H), 7.90 (s, 1H), 8.34–8.36 (m, 1H), 8.52–8.55 (m, 1H), 8.85 (t, *J* = 1.8 Hz, 1H). ^13^C NMR (125 MHz, CDCl_3_): δ ppm 47.41, 56.25, 61.10, 107.60, 119.93, 123.09, 128.44, 128.48, 130.42, 133.66, 135.10, 135.37, 140.57, 148.53, 153.65, 165.60, 167.48, 188.16.

#### 2.3.6 (E/Z)-3-(2-(3-Methoxyphenyl)-2-oxoethyl)-5-(3,4,5-trimethoxybenzylidene)­thiazolidine-2,4-dione **(5)**

Compound **PZ-5** was prepared according to the general method starting from 3-(2-(3-methoxyphenyl)-2-oxoethyl)­thiazolidine-2,4-dione (1 mmol, 0.265 g) and 3,4,5-trimethoxybenzaldehyde (1.2 mmol, 0.235 g). The residue was purified by cc using the mixture of chloroform/*n*-hexane (4:1) as the eluent. Mp 182–183 °C, (63% yield). MS (ESI^+^) (%): 909.9 ([2 M + Na]^+^, 100%), 466.59 ([M + Na]^+^, 30%), ^1^H NMR (500 MHz, DMSO-d6): δ ppm 3.04 (s, 3H), 3.86 (d, *J* = 1.5 Hz, 9H), 5.34 (s, 2H), 7.00 (s, 2H), 7.31–7.33­(m, 1H), 7.51–7.57 (m, 2H), 7.70 (d, *J* = 7.9 Hz, 1H), 7.97­(s, 1H). ^13^C NMR (125 MHz, DMSO-d6): δ ppm 22.99, 48.53, 55.94, 56.54, 60.72, 108.26, 113.17,120.23, 121.12, 121.19, 128.78, 130.73, 134.64, 135.56, 140.25, 153.74, 160.03, 165.64, 167.48,173.34, 191.66.

#### 2.3.7 (E/Z)-3-(2-(4-bromophenyl)-2-oxoethyl)-5-(3,4,5-trimethoxybenzylidene)­thiazolidine-2,4-dione **(6)**

Compound **PZ-6** was prepared according to the general method starting from 3-(2-(4-bromophenyl)-2-oxoethyl)­thiazolidine-2,4-dione (1 mmol, 0.314 g) and 3,4,5-trimethoxybenzaldehyde (1.2 mmol, 0.235 g). The residue was purified by cc using the mixture of chloroform/*n*-hexane (4:1) as the eluent. Mp 180–181 °C, (66% yield). MS (ESI^+^) (%): 514.27 ([M + Na]^+^, 40%), 167.24 (C_9_H_11_O_3_^+^; trimethoxybenzyl cation, %75). ^1^H NMR (500 MHz, CDCl_3_): δ ppm 3.94 (d, *J* = 2 Hz, 9H), 5.15 (s, 2H), 6.78 (s, 2H), 7.69 (d, *J* = 8.5 Hz, 2H), 7.87 (d, *J* = 8.9 Hz, 3H). ^13^C NMR (125 MHz, CDCl_3_): δ ppm 47.27, 56.24, 61.08, 107.55, 120.16, 128.54, 129.61,129.64, 132.37, 132.90, 134.78, 140.45, 153.63, 165.74, 167.56, 188.94.

#### 2.3.8 (E/Z)-3-(2-(3,4-Dichlorophenyl)-2-oxoethyl)-5-(3,4,5-trimethoxybenzylidene)­thiazolidine-2,4-dione **(7)**

Compound **PZ-7** was prepared according to the general method starting from 3-(2-(3,4-dichlorophenyl)-2-oxoethyl)­thiazolidine-2,4-dione (1 mmol, 0.304 g) and 3,4,5-trimethoxybenzaldehyde (1.2 mmol, 0.235 g). The residue was purified by cc using the mixture of chloroform/*n*-hexane (4:1) as the eluent. Mp 157–158 °C, (58% yield). MS (ESI^+^) (%): 987.9 ([2 M + Na]^+^, 100%), 167.24 (C_9_H_11_O_3_^+^; trimethoxybenzyl cation, 90%). ^1^H NMR (500 MHz, CDCl_3_): δ ppm 3.94 (d, *J* = 2 Hz, 9H), 5.14 (s, 2H), 6.78 (s, 2H), 7.64 (d, *J* = 8.5 Hz, 1H), 7.84 (dd, *J* = 8.4 Hz, *J* = 2 Hz, 1H), 7.89 (s, 1H), 8.96 (d, *J* = 2 Hz, 1H). ^13^C NMR (125 MHz, CDCl_3_): δ ppm 47.23, 56.25, 61.09, 107.57, 120.03, 127.08, 128.48, 130.16, 131.17, 133.67, 133.88, 134.95, 139.97, 140.52, 140.35, 153.64, 165.64, 167.49, 187.97.

#### 2.3.9 (E/Z)-5-(4-Chlorobenzylidene)-3-(2-(4-fluorophenyl)-2-oxoethyl)­thiazolidine-2,4-dione **(8)**

Compound **PZ-8** was prepared according to the general method starting from 3-(2-(4-fluorophenyl)-2-oxoethyl)­thiazolidine-2,4-dione (1 mmol, 0.253 g) and 4-chlorobenzaldehyde (1.2 mmol, 0.168 g). The residue was purified by cc using the mixture of chloroform/*n*-hexane (4:1) as the eluent. Mp 198 °C, (68% yield). MS (ESI^+^) *m*/*z* (%): 241.36 (C_10_H_8_ClNO_2_S^+^, 40%), 179.33 (C_9_H_6_ClNO^+^, 100%), 157.73 (C_5_H_4_NO_3_S^+^, 80%). ^1^H NMR (500 MHz, CDCl_3_): δ ppm 5.17 (s, 2H), 7.20–7.24 (m, 2H), 7.48 (s, 4H), 7.91 (s, 1H), 8.03–8.06 (m, 2H). ^13^C NMR (125 MHz, CDCl_3_): δ ppm 47.32, 116.20, 116.38, 121.89, 129.63, 130.61, 130.64, 130.89, 130.97, 131.38, 131.61, 133.12, 136.87, 165.71, 167.27, 188.14

#### 2.3.10 (E/Z)-5-(4-Chlorobenzylidene)-3-(2-(3-methoxyphenyl)-2-oxoethyl)­thiazolidine-2,4-dione **(9)**

Compound **PZ-9** was prepared according to the general method starting from 3-(2-(3-methoxyphenyl)-2-oxoethyl)­thiazolidine-2,4-dione (1 mmol, 0.265 g) and 4-chlorobenzaldehyde (1.2 mmol, 0.168 g). The residue was purified by cc using the mixture of chloroform/*n*-hexane (4:1) as the eluent. Mp 209 °C, (55% yield). MS (ESI^+^) *m*/*z* (%): 241.36 (C_10_H_8_ClNO_2_S^+^, 60%), 179.33 (C_9_H_6_ClNO^+^, 100%), 157.86 (C_5_H_4_NO_3_S^+^, 60%). ^1^H NMR (500 MHz, CDCl_3_): δ ppm 3.88 (s, 3H), 5.18 (s, 2H), 7.19–7.21 (m, 1H), 7.43–7.46 (t, *J* = 8.05, 1H), 7.48 (s, 4H), 7.51–7.52 (m, 1H), 7.58–7.59 (m, 1H), 7.90 (s,1H). ^13^C NMR (125 MHz, CDCl_3_): δ ppm 47.32, 116.20, 116.38, 121.89, 129.63, 130.61, 130.64, 130.89, 130.97, 131.38, 131.61, 133.12, 136.87.

#### 2.3.11 *(E/Z*)-5-(4-Chlorobenzylidene)-3-(2-(3,4-dichlorophenyl)-2-oxoethyl)­thiazolidine-2,4-dione **(10)**

Compound **PZ-10** was prepared according to the general method starting from 3-(2-(3,4-dichlorophenyl)-2-oxoethyl)­thiazolidine-2,4-dione (1 mmol, 0.304 g) and 4-chlorobenzaldehyde (1.2 mmol, 0.168 g). The residue was purified by cc using the mixture of chloroform/*n*-hexane (4:1) as the eluent. Mp 250 °C, (75% yield). MS (ESI^+^) *m*/*z* (%): 463.00 ([M + K]^+^, 10%), 241.42 (C_10_H_8_ClNO_2_S^+^, 30%), 179.22 (C_9_H_6_ClNO^+^, 100%), 157.48 (C_5_H_4_NO_3_S^+^, 60%). ^1^H NMR (500 MHz, CDCl_3_): δ ppm 5.14 (s, 2H), 7.49 (s, 4H), 7.63–7.65 (d, *J* = 8 Hz, 1H), 7.82–7.84 (dd, *J* = 2 Hz, 1H), 7.92 (s, 1H), 8.091–8.095 (d, *J* = 2 Hz, 1H). ^13^C NMR (125 MHz, CDCl_3_): δ ppm 47.29, 121.73, 127.07, 129.66, 130.16, 131.18, 131.39, 131.55, 133.34, 133.63, 133.91, 136.97, 139.12

#### 2.3.12 (E/Z)-5-(4-Chlorobenzylidene)-3-(2-(3-nitrophenyl)-2-oxoethyl)­thiazolidine-2,4-dione **(11)**

Compound **PZ-11** was prepared according to the general method starting from 3-(2-(3-nitrophenyl)-2-oxoethyl)­thiazolidine-2,4-dione (1 mmol, 0.280 g) and 4-chlorobenzaldehyde (1.2 mmol, 0.168 g). The residue was purified by cc using the mixture of chloroform/*n*-hexane (4:1) as the eluent. Mp 220 °C, (50% yield). MS (ESI^+^) *m*/*z* (%): 425.68 ([M + Na]^+^, 10%), 241.33 (C_10_H_8_ClNO_2_S^+^, 48%), 179.17 (C_9_H_6_ClNO^+^, 100%), 157.70 (C_5_H_4_NO_3_S^+^, 95%). ^1^H NMR (500 MHz, CDCl_3_): δ ppm 5.15 (s, 2H), 7.49 (s, 4H), 7.70 (d, *J* = 8.65 Hz, 2H), 7.86–7.91 (m, 3H). ^13^C NMR (125 MHz, CDCl_3_): δ ppm 47.33, 121.86, 129.60, 129.64, 129.68, 131.38, 131.60, 132.39, 132.88, 133.18, 136.90.

### 2.4 Cell Viability Analysis

#### 2.4.1 Cell Culture

MCF-7 breast cancer cell line was cultured in Dulbecco’s Modified Eagle Medium (Gibco, Thermo Fisher Scientific, Inc., Waltham, MA) supplemented with 10% (v/v) fetal bovine serum (Gibco, Thermo Fisher Scientific, Inc., Waltham, MA) and 1% penicillin-streptomycin in an incubator (Thermo Fisher Scientific Steri-Cycle, CO_2_ Incubator, Japan) at 37 °C with 5% CO_2_.

#### 2.4.2 MTT Assay

The MTT assay is a colorimetric technique commonly used to assess cell viability, proliferation, and cytotoxicity of chemical compounds. It is based on the ability of metabolically active cells to reduce the yellow tetrazolium salt (MTT) to purple formazan crystals via mitochondrial dehydrogenase enzymes. The amount of formazan produced is directly proportional to the number of viable cells and can be quantified by measuring the absorbance at 570 nm using a microplate reader. This method provides a reliable and sensitive approach for evaluating the cytotoxic effects of new drug candidates *in vitro*.

The cytotoxic potential of the PZ compounds against MCF-7 breast cancer cells was initially determined by an MTT assay. Vincristine (Koçak Pharma) was used as a reference drug for cytotoxicity analysis. Cells were seeded into 96-well plates at a density of 1 × 10^4^ cells/mL and incubated for 24 h to allow cell adherence. After incubation, cells were treated with three different concentrations of the compounds (5, 50, and 100 μM) dissolved in DMSO for 48 h. Control groups received DMSO alone at a final concentration of not exceeding 0.1%. The cytotoxic effects of the synthesized compounds were compared with those of vincristine (3, 6.5, and 10 μM).^14,15^

#### 2.4.3 xCELLigence Real-Time Cell Analyzer (RTCA) System

According to the MTT results, the two compounds with the highest cytotoxicity on MCF-7 breast cancer cells (PZ-9 and PZ-11) were selected, and their IC_50_ concentrations were determined by xCelligence real-time cell analysis (xCELLigence RTCA S16, Acea Biosciences, San Diego, CA). In the 16-well e-plate, 100 μL of the cell culture medium was added to each well and measured to ensure normalization. Subsequently, MCF-7 breast cancer cells were seeded at a rate of 5 × 10^3^ cells per well in the laminar flow. The cells were then incubated in the laminar flow for half an hour to allow for uniform distribution across the bottom of the e-plate. After the designated period, the e-plate was returned to the xCELLigence RTCA S16 instrument, which was placed in an incubator. The requisite parameters were applied, and the program was activated to set the measurement interval at 15 min and the total analysis time at 120 h. Following a 24-h incubation period, the instrument was terminated, and the 16-well e-plate was removed from the xCELLigence RTCA S16 incubator. From each well of the e-plate, 100 μL of the medium was removed and discarded. This was followed by the addition of 100 μL of PZ-9 and PZ-11 at concentrations of 5, 8, 10, 15, 20, 25, and 50 μM. The appropriate medium for the cell line was used as control. The experiment was completed at 160 h by monitoring the measurements, and RTCA Software Lite was used to calculate IC_50_ concentration.

### 2.5 Gene Expression Analysis

#### 2.5.1 Total RNA Extraction and cDNA Synthesis

MCF-7 breast cancer cells were incubated with an IC_50_ concentration of PZ-11, which is found as the most cytotoxic compound, for 48 h. After the incubation period, total RNA was extracted from PZ-11-treated and nontreated MCF-7 breast cancer cells using TRIzol reagent (Invitrogen, 15596026, Waltham, MA). The quality and quantity of extracted RNAs were assessed by optical density measurement using a spectrophotometer (NanoDrop, ND-1000, Wilmington, DE). cDNA synthesis was performed using the iScript cDNA Synthesis Kit according to the manufacturer’s instructions (Bio-Rad Laboratories, Hercules, CA). The reaction was incubated at 42 °C for 60 min to allow for reverse transcription, followed by an inactivation step at 70 °C for 5 min to terminate the reaction.

#### 2.5.2 Quantitative Real-Time PCR (qRT-PCR)

The synthesized cDNA was then used for downstream applications, including qRT-PCR to quantify the expression levels of the target genes. Quantitative real-time PCR analysis was performed with RT^2^ Profiler PCR Array (Qiagen) (Figure 3). Cycling conditions were as follows: initial denaturation at 95 °C for 10 min, followed by 45 cycles of 95 °C for 15 s and 60 °C for 1 min. The expression levels were normalized against those of the housekeeping gene to account for variations in RNA input and cDNA synthesis efficiency. The results were analyzed using the comparative 2−ΔΔCT method to determine relative gene expression levels across PZ-11-treated and nontreated MCF-7 breast cancer cells.

**Figure 3:**
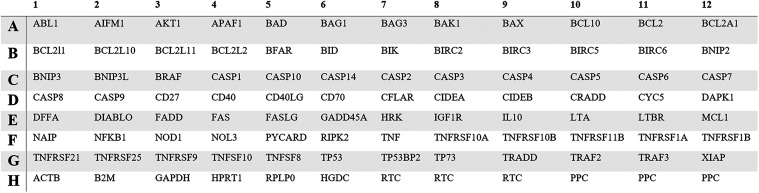
Layout for the RT^2^ Profiler PCR Array (HGDC: human genomic DNA contamination; RTC: reverse transcription control; PPC: positive PCR control).

### 2.6 Statistical Analyses

Statistical analyses were conducted using RTCA Software Lite (ver. 2.0) and GraphPad Prism (version 9.5.1). The RTCA Software Lite was utilized for real-time cell analysis, enabling continuous cell proliferation and viability monitoring through impedance measurements. This software facilitated the assessment of cellular responses to various treatments over time, providing dynamic data crucial for evaluating experimental conditions’ effects on cell behavior. GraphPad Prism was employed to perform statistical evaluations for the analysis of the quantitative data. Data were expressed as the mean ± standard deviation (SD) or the mean ± standard error of the mean (SEM), as appropriate. Comparisons between groups were made using one-way analysis of variance (ANOVA), followed by post-hoc tests (Dunnett’s test) to determine significant differences among groups. A *p*-value of less than 0.05 was considered statistically significant. GraphPad Prism was also used to generate graphs and visual representations of the data, enhancing the clarity and interpretability of the results.

### 2.7 Computational Studies

#### 2.7.1 Molecular Docking Validation

The crystal structures of proteins encoded by the most significant genes in gene expression analysis were imported from the RCSB Protein Data Bank^16^ into Konstanz Information Miner (KNIME)^17^ as starting inputs for the molecular docking workflow. First, in the KNIME workflow (Figure 4), we used the “Get PDB” node to import the input PDB crystal structures. The structures included Tubulin polymerase vinca-domain (5J2T, 2.20 Å), Tubulin α/β-subunits (1SA0, 3.58 Å), apoptosis-inducing factor 1 (AIF) (4LII, 1.88 Å), death-associated protein kinase 1 (DAPK1) (9INV, 1.61 Å), tumor necrosis factor α (TNFa) (2AZ5, 2.10 Å), and insulin-like growth factor 1 receptor (IGF-1R) (2OJ9, 2.00 Å). Then, the validation process was performed before screening of PZ-11 against these target proteins. For validation, we extracted the coligands using the “split by structure” node followed by the “row filter” node, and then the coligands were subjected to a ligand preparation process using the “Ligprep” node in order to add polar hydrogens and perform Epik integration and energy minimization using the OPLS4 force field. Target proteins were subjected to a protein preparation process as well in order to add polar hydrogens, delete water and cofactors, if needed, and fill in missing side chains and loops using Prime, also to perform p*K*_a_ calculation using PROPKA, Epik integration, and energy minimization using OPLS4 force field. After determining the grid coordinates using the Glide Grid Generation tool, the original bound coligands were redocked using the Glide Ligand Docking tool in order to compare the redocked pose to the original complex and to calculate the RMSD between the two aligned poses using the RMSD node (Figure 4).

**Figure 4:**
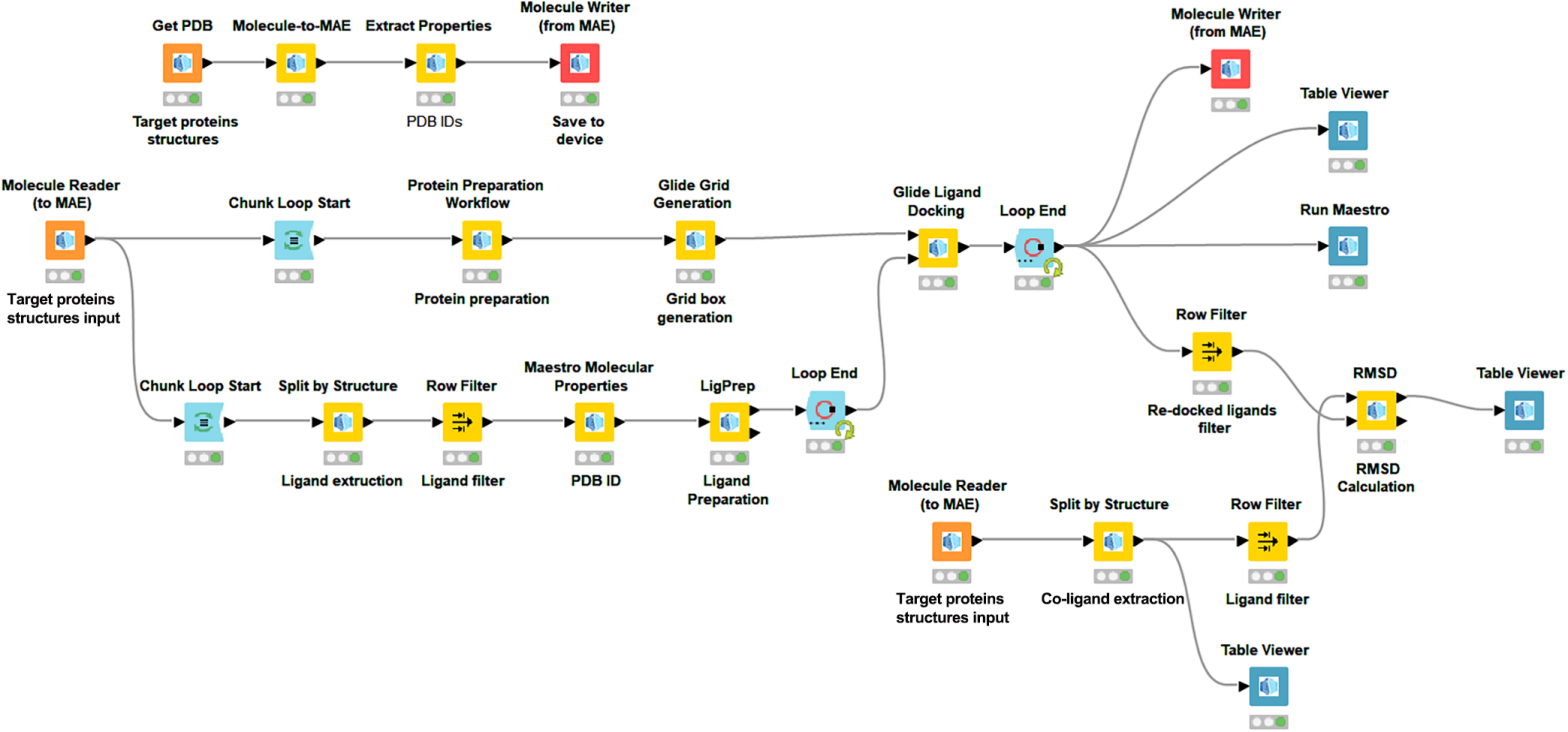
Snapshot of a KNIME workflow used to perform molecular docking validation.

#### 2.7.2 Molecular Docking

Based on the activity results obtained against the MCF-7 breast cancer cells, PZ-11 was selected as the most cytotoxic compound. The chemical structure of the most active compound was drawn in ChemDraw Ultra Version 12.0 software^18^ and was saved in SDF format, using the “Molecule reader” node. PZ-11 was imported into KNIME in MAE format and was subjected to the ligand preparation process. Since the Grid coordinates for every target protein were validated by producing redocked poses with RMSD less than 2, the same docking protocols were used to carry out docking trials for PZ-11 (Figure 5). Molecular docking results and interaction profile analyses were investigated by using Maestro Schrodinger Release 2024-3.^19^

**Figure 5:**
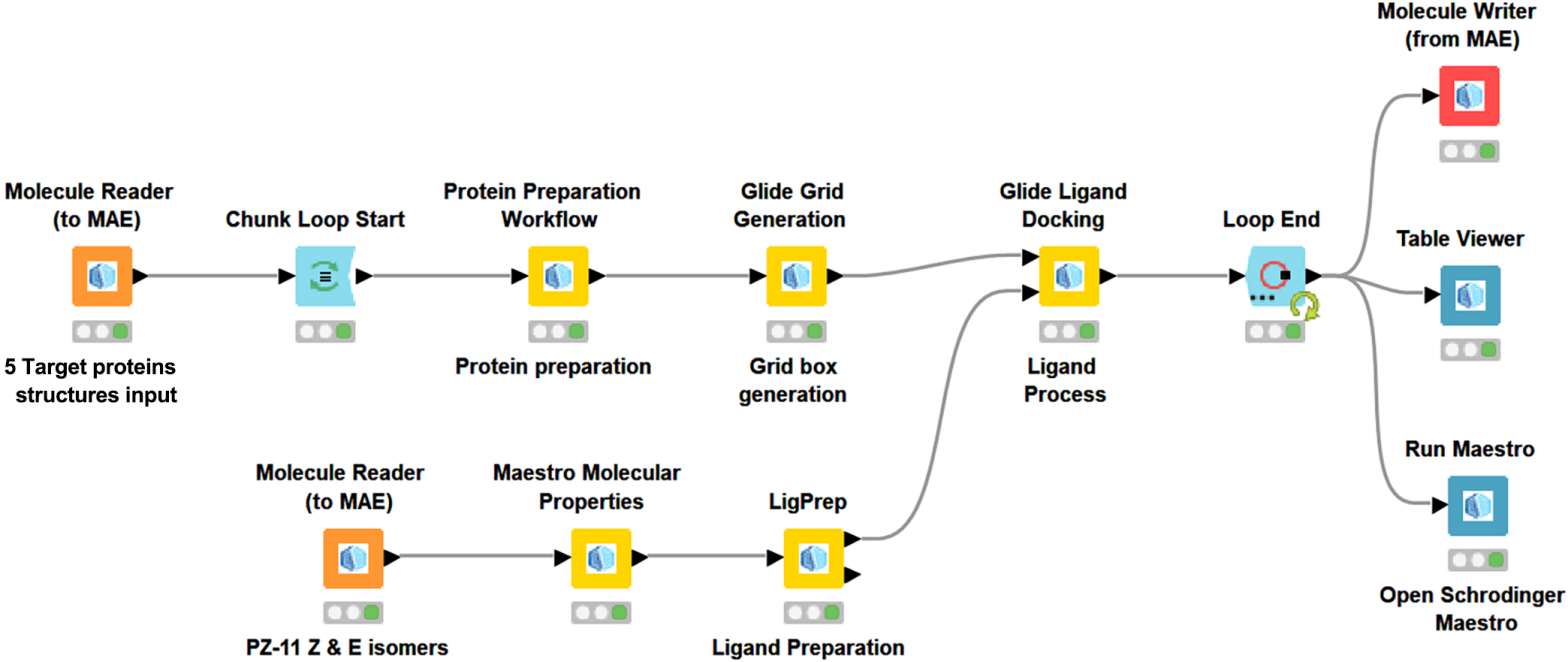
Snapshot of a KNIME workflow used to perform molecular docking trials for PZ-11.

#### 2.7.3 Molecular Dynamics Simulation

The molecular dynamics (MD) simulation was performed using GROMACS version 2025.2.^20^ GROMACS is an open-source, high-performing molecular dynamics simulation software package that provides several advanced techniques for free-energy calculations with a high degree of accuracy and efficiency.^21^ The MDS was performed on a workstation with the following properties: Ubuntu 22.04.5 LTS 64-bit, AMD Ryzen 7 5800X 8-Core CPU with 4 gigabytes dedicated NVIDIA GeForce graphics card, CUDA version: 12.8, 32 GB RAM.

Our active compound, PZ-11, was processed by saving the docked file into pdb format using the Schrodinger Maestro program. For system preparation, the OPLS-AA/L all-atom force field was used for preparing the topology and “.gro” files of the AIF protein (PDB ID: 4LII) using the pdb 2gmx module in GROMACS. Then, LigParGen online Web server^22–24^ was used to generate the “.itp” and “.gro” files for ligand (PZ-11) after applying the OPLS-AA force field. Later on, ligand topology was rejoined to the processed protein structures for building the complex system. A water-solvated system was built by using the TIP4P-Ew water model with dodecahedral periodic boundary conditions. The solvated system was neutralized by adding two Cl anions. Energy minimization was done at 1000 kJ/mol/nm with the steepest descent Algorithm by using the Verlet cutoff scheme, taking Particle Mesh Edward (PME) Coulombic interactions with a maximum of 50,000 steps. Prior to the production simulation, the system underwent a 0.4 ns pre-equilibration phase under both constant volume (NVT) and constant pressure (NPT) ensembles, allowing it to reach thermodynamic stability at 300 K and 1 atom. First, NVT equilibration takes V-rescale (temperature coupling) at 300 K, while in the second step, NPT equilibration takes C-rescale (pressure coupling), 1 bar reference pressure, and 400 ps of steps. The simulation protocol ensured thermal and pressure equilibration of the system, thereby establishing physiologically relevant conditions for molecular dynamics (MD) simulations.

A production run of 200 ns was conducted, offering a sufficient time scale to capture the dynamic behavior and intermolecular interactions within the biomolecular assembly. Throughout the simulation, key thermodynamic and structural parameters were monitored to assess the system stability by saving the simulation trajectory every 10 ps. The simulation results were incorporated into the GROMACS default script. The root-mean-square deviation (RMSD), root-mean-square fluctuation (RMSF), radius of gyration (Rg), and hydrogen-bond profiling were employed to investigate the conformational dynamics and stability of the protein–ligand complex. Finally, interaction profiles after 50 ns, 100 ns, and 190 ns were created and were visualized using the PyMOL Molecular Graphics System v2.5.4.^25^

### 2.8 Prediction of ADME Properties

For the estimation of the ADME properties of the thiazolidinedione derivatives, SMILES codes were generated with the ChemDraw Ultra Version 12.0 software.^18^ SMILES codes for the commercialized reference compound—vincristine—was taken from PubChem. Consequently, all of them were submitted as input information to the SwissADME online program.^26^ Molecular parameters such as consensus log Po/w, permeation through the BBB, P-glycoprotein substrate characteristics, gastrointestinal absorption, CYP450 inhibition, number of rotatable bonds, the topological polar surface area TPSA (Å^2^), and accordance with the Lipinski and Veber filters were evaluated.

### 2.9 In Silico Toxicity Assessment

Metabolic transformations (Phase I and II) of our compounds were predicted using BioTransformer 3.0,^27^ a comprehensive computational tool, which combines rule-based and machine-learning methods with a curated database of known biochemical reactions to predict likely metabolite structures along with their associated reaction types and enzymes (e.g., CYP450, conjugation). To make the predictions more focused and manageable, SMARTCyp^28^ was used to identify the most likely sites in the molecules where CYP450 enzymes would carry out reactions, based on chemical reactivity and molecular accessibility. Based on these results, the two most probable metabolites (as determined by predicted likelihood, reactivity, or abundance) were selected for further genotoxic carcinogenicity assessment. These candidate metabolites were analyzed using Toxtree,^29^ a decision-tree and structural alert–based tool that applies the Benigni/Bossa mutagenicity/carcinogenicity rule base and DNA-binding alerts to identify potential genotoxic or carcinogenic features in the metabolite structures. In this way, the workflow provided a mechanistic *in silico* screening pathway from the parent compound to the metabolite prediction and evaluation of genotoxic carcinogenicity.

## 3 Results and Discussion

### 3.1 Chemical Synthesis

In this study, two series of compounds were designed, synthesized, and evaluated for their biological activity. The core scaffold in both series features a thiazolidinedione moiety that is functionalized at the nitrogen atom with a variety of substituted acetophenones. The thiazolidinedione was further substituted at the fifth carbon with an olefinic linker connecting either 3,4,5-trimethoxybenzaldehyde (Series 1) or para-chlorobenzaldehyde (Series 2) (Figure 2).

In the first series (compounds PZ**1–7**), the thiazolidinedione core was linked to 3,4,5-trimethoxybenzaldehyde, while in the second series (compounds PZ**8–11**), the core was linked to para-chlorobenzaldehyde. The different substituents (R) were attached to the aromatic ring of the acetophenone moiety. These derivatives were chosen to explore the effect of different electronic properties and steric factors on the biological activity of the compounds. The substituents represent a range of electron-withdrawing (e.g., nitro, fluoro, bromo, and dichloro) and electron-donating groups (e.g., methoxy), as well as variations in steric bulk. By systematically varying the substituents on the aromatic ring, this study aims to investigate the SAR of the synthesized compounds to optimize their biological efficacy and selectivity.

The synthesis pathway of the hybridized final compounds was outlined in Figure 2. The synthesis begins with the preparation of thiazolidine-2,4-dione by reacting chloroacetic acid (10 g) with thiourea (8.55 g) in a sealed flask containing 10 mL of water under reflux conditions. The completion of the reaction was monitored using TLC. Upon completion, the precipitate was filtered, washed with water, dissolved in methanol, and recrystallized. Thiazolidine-2,4-dione (0.01 mol) was then dissolved in 10 mL of methanol, and a solution of potassium hydroxide (0.01 mol) in 6.5 mL of methanol was added dropwise. The mixture was stirred for 10 min before adding substituted or unsubstituted acetophenone (0.01 mol). The reaction mixture was stirred for another 5 min and then heated under reflux for 40 h. Once the reaction is complete, the mixture was filtered and rinsed with methanol. The resulting precipitate was recrystallized using ethanol to obtain the intermediate compounds. In the final step, a solution of trimethoxybenzaldehyde (1.2 mmol) or 4-chlorobenzaldehyde in glacial acetic acid (4 mL) was prepared, and (substituted or unsubstituted phenacyl)-2,4-thiazolidinedione (1 mmol), along with sodium acetate (0.4 g), was added. The reaction mixture was refluxed at 170–180 °C for 40 h. The resulting precipitate was filtered, washed with water and methanol, and purified using column chromatography with silica gel 60 (230–400 mesh ASTM). A mixture of *n*-hexane and chloroform (4:1) or *n*-hexane and ethyl acetate (3:1) was used as the eluting solvent to purify the final compounds.^6,14^

### 3.2 Evaluation of Cytotoxic Activity

#### 3.2.1 Cytotoxicity Analysis

In order to ascertain the cytotoxic effects of the synthesized compounds (PZ-1–PZ-11) on the MCF-7 breast cancer cells, initially, an MTT assay was conducted at 48 h. Vincristine, a tubulin polymerase inhibitor that is widely used in the treatment of breast cancer, was used as a reference drug. The synthesized compounds were tested at three different concentrations (5, 50, and 100 μM), in order to determine their cytotoxic range over MCF-7 breast cancer cells. Upon analysis of the results, it became evident that the PZ-9 and PZ-11 compounds exhibited the most cytotoxic effect on MCF-7 breast cancer cells (Table 1). In our study, compounds in Series 2 (parachlorobenzene derivatives) showed significantly greater cytotoxicity against MCF-7 cells than those in Series 1 (3,4,5-trimethoxybenzene derivatives). Prior research on 5-benzylidene-2,4-thiazolidinediones has demonstrated that, in contrast to electron-donating or neutral substituents, electron-withdrawing substituents (such as Chloro) on the benzylidene phenyl ring might increase antiproliferative activity in breast cancer lines, including MCF-7 ^30^ and these findings are consistent with the results obtained in our study.

**Table 1:** Cytotoxic Dose Range of the Compounds at Low, Medium, and High Doses on the MCF-7 Breast Cancer Cells

**Compound #**	**Substitution**	**5 μM**	**50 μM**	**100 μM**	**Toxic range (μM)**	**Selected compounds**
**PZ-1**	unsubstituted	nontoxic	nontoxic	toxic	100	
**PZ-2**	2-fluoro	nontoxic	nontoxic	toxic	100	
**PZ-3**	4-fluoro	nontoxic	nontoxic	toxic	50–100	
**PZ-4**	3-nitro	nontoxic	nontoxic	toxic	100	
**PZ-5**	3-methoxy	nontoxic	nontoxic	toxic	50–100	
**PZ-6**	4-bromo	nontoxic	toxic	toxic	5–50	
**PZ-7**	3,4-dichloro	nontoxic	toxic	toxic	5–50	
**PZ-8**	4-fluoro	nontoxic	toxic	toxic	50	
PZ-9**	3-methoxy	nontoxic	toxic	toxic	5–50	the **second cytotoxic** compound
**PZ-10**	3,4-dichloro	nontoxic	toxic	toxic	5–50	
PZ-11*	3-nitro	nontoxic	toxic	toxic	5–50	the **most cytotoxic** compound

Cytotoxicity analyses were continued with the xCELLigence real-time cell analysis method to determine the IC_50_ concentrations of the two most cytotoxic compounds (PZ-9 and PZ-11) and the reference drug, vincristine. The IC_50_ value of Vincristine was determined to be 6.8 μM, as evidenced by the experimental results (Figure 6 and Table 2). The results of the experiment demonstrated that PZ-9, at concentrations of 5, 8, 10, 15, 20, 25, and 50 μM, exerts a suppressive effect on the MCF-7 breast cancer cells, as 22.83, 33.91, 34.68, 37.43, 41.52, 46.02, and 65.04% (Figure 6), respectively. Upon analysis of the experimental results, the IC_50_ concentration of PZ-9 on the MCF-7 breast cancer cells was determined to be 29.44 μM (Table 2). The xCELLigence RTCA method was also employed to investigate the antiproliferative and cytotoxic effects of PZ-11 on MCF-7 breast cancer cells. The results of the experiment demonstrated that PZ-11 at concentrations of 5, 8, 10, 15, 20, and 25 μM exhibited a suppressive effect on the MCF-7 breast cancer cells, with the percentage inhibition reaching 20.83, 30.02, 32.33, 48.25, 51.27, and 60.13% (Figure 6), respectively. Upon analysis of the experimental results, the IC_50_ concentration of PZ-11 on the MCF-7 breast cancer cells was determined to be 17.35 μM (Table 2).

**Figure 6:**
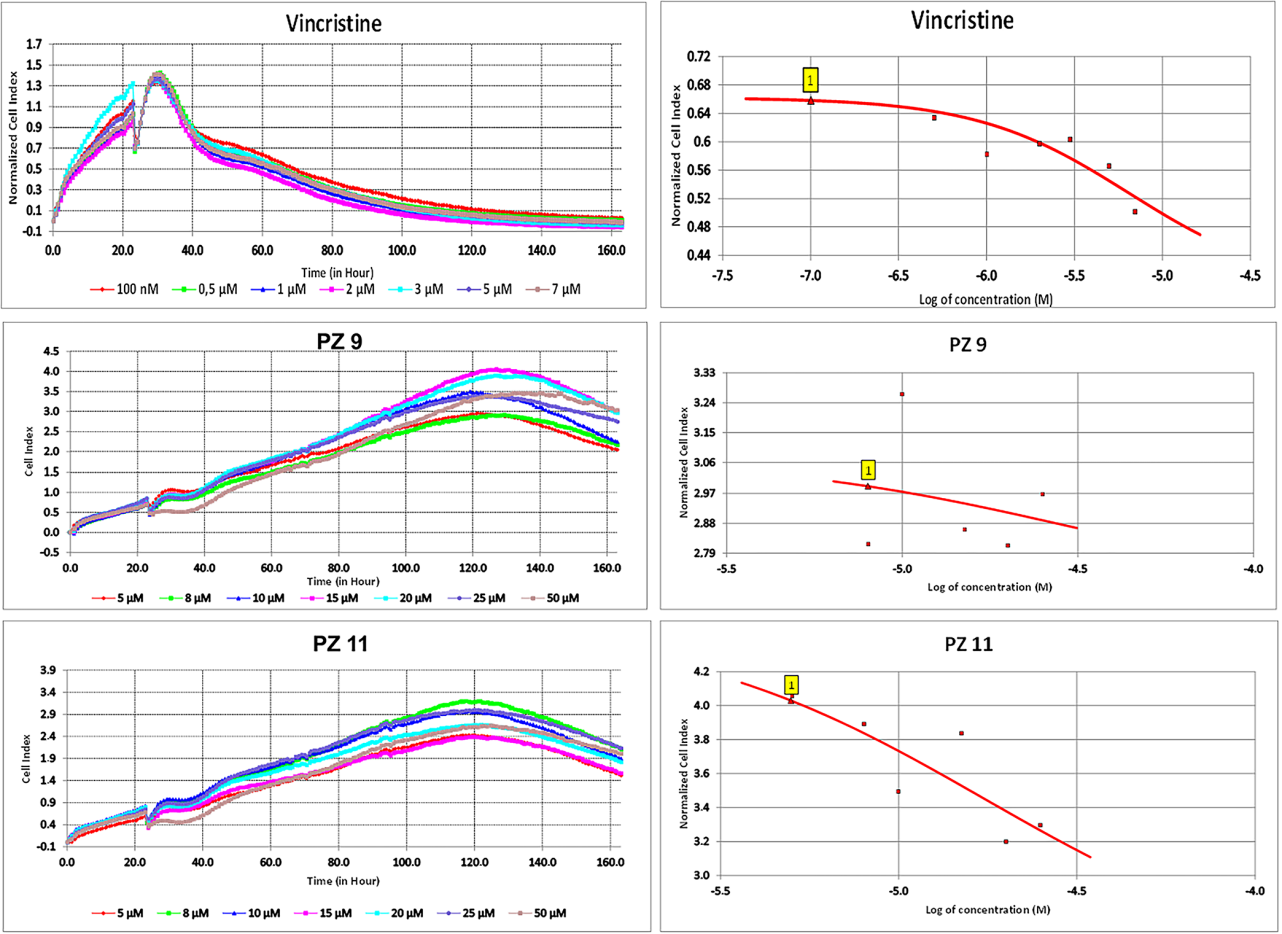
Cytotoxic effect of Vincristine, PZ-9, and PZ-11 on MCF-7 breast cancer cells.

**Table 2:** Inhibitory Concentrations (IC_50_) of Vincristine, PZ-9, and PZ-11 on MCF-7 Breast Cancer Cells

Compounds	IC_50_ (μM)
Vincristine	6.45
PZ-9	29.44
PZ-11	17.35

### 3.3 Gene Expression Analysis

According to both MTT and xCELLigence RTCA results, PZ-11 was found as the most cytotoxic compound with respect to the reference drug. Thus, qRT-PCR analysis was continued with the PZ-11-treated and nontreated MCF-7 breast cancer cells in order to show the effect of the compound on gene expression level. In qRT-PCR analyses, changes in the expression levels of a total of 88 genes related to apoptosis in MCF-7 breast cancer cells were determined due to PZ-11 application. Within the scope of the analyses, gene expression levels increasing and decreasing more than 2-fold with respect to the nontreated control group were considered as statistically significant (*p* < 0.05) (Table 3 and Figure 7).

**Figure 7:**
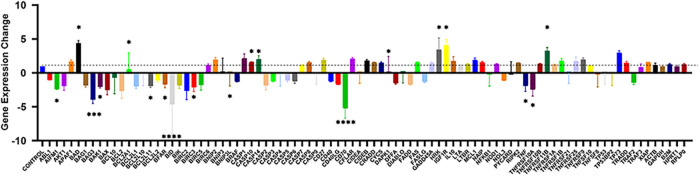
Changes in gene expression levels in MCF-7 breast cancer cells due to the treatment of the PZ-11 compound. GAPDH was used as a housekeeping gene (*p < 0.05 ** *p* < 0.01; *** *p* < 0.001; **** *p* < 0.0001).

**Table 3:** Effect of PZ-11 on the Significance Expression Levels of Apoptosis-Related Genes in MCF-7 Breast Cancer Cells (*p < 0.05)

**Genes**	**Fold regulation ± standard deviation**
*AIFM1*	–2.38 ± 0.04
*BAD*	4.68 ± 0.38
*BAG3*	–4.34 ± 0.55
*BAK1*	–1.92 ± 0.18
*BCL2A1*	2.26 ± 1.40
*BCL2L11*	–2.08 ± 0.16
*BFAR*	–2.04 ± 0.50
*BID*	–4.07 ± 1.28
*BIRC3*	–2.79 ± 0.61
*BNIP3L*	1.28 ± 0.88
*CASP10*	1.59 ± 0.05
*CASP14*	1.64 ± 0.53
*CD70*	–6.25 ± 1.47
*DAPK1*	1.08 ± 1.24
*HRK*	2.22 ± 1.71
*IGF1R*	4.68 ± 0.82
*TNF*	–2.50 ± 0.81
*TNFRS10A*	–3.13 ± 0.95
*TNFRS11B*	2.93 ± 0.48

The current study successfully synthesized novel compounds through molecular hybridization, focusing on the inhibition of tubulin polymerization, a critical process in mitosis that is often dysregulated in cancer cells. The design of these compounds based on the Thiazolidinedione scaffold reflects a strategic approach to enhance anticancer efficacy by combining different pharmacophores. This method has been previously validated as compounds targeting tubulin polymerization have shown significant antiproliferative effects in various cancer models, including breast cancer.^31^

In this study, vincristine, a well-known tubulin inhibitor, was used as a reference drug for the evaluation of the cytotoxic effects of PZ compounds. In the literature, the IC_50_ concentration of vincristine on MCF-7 breast cancer cells was found as 5.45 μM.^15^ Correlatively, the IC_50_ concentration of vincristine was determined as 6.45 μM in our results. Cytotoxicity assays conducted against MCF-7 breast cancer cells revealed that several of the synthesized compounds (PZ-9 and PZ-11) exhibited significant highest antiproliferative activity. This finding aligns with previous studies indicating that compounds targeting tubulin polymerization can effectively inhibit cancer cell proliferation.^32,33^ Beyond their traditional function as PPARγ agonists, TZD derivatives also influence a variety of oncogenic pathways, such as apoptosis, proliferation, angiogenesis, and inflammatory signaling.^34^ Specifically, it is shown by structure–activity relationship (SAR) investigations that hybridizations and substituent effects have a significant impact on both the potency and selectivity. According to our results, PZ-11, a TZD derivative hybridized with structural characteristics that target microtubules, exhibits strong antiproliferative and pro-apoptotic properties and probably benefits from comparable SAR optimizations.

The strategic incorporation of microtubule-disrupting structural motifs into the TZD scaffold in the design of PZ-11 aligns closely with recent trends in anticancer drug development, particularly in the design of hybrid molecules. Such compounds often exhibit enhanced antiproliferative effects by simultaneously modulating multiple cancer-related pathways, including cell cycle regulation and apoptosis induction. For instance, in a recent study, 2,4-thiazolidinedione derivatives were developed as hybrid agents capable of both disrupting the Wnt/β-catenin/TCF4 interaction and inhibiting tubulin polymerization, leading to G2/M cell cycle arrest and apoptosis in colon cancer cells.^35^ Similarly, thiazolidinone-constrained combretastatin analogues have been synthesized by integrating TZD-like scaffolds with microtubule-targeting pharmacophores, resulting in effective disruption of microtubule assembly and inhibition of cancer cell proliferation.^36^ Moreover, α-phthalimido-chalcone hybrids, which act as dual HDAC/tubulin inhibitors, have been shown to suppress β-tubulin polymerization, induce G2/M arrest, and trigger apoptosis in cancer cells.^37^ Collectively, these studies underscore the potential of hybrid molecules like PZ-11, which combine TZD cores with microtubule-interfering motifs, to exert dual-action effects on cell division and survival mechanisms, thereby enhancing their antiproliferative efficacy.

The observed cytotoxic effects at varying concentrations suggest a dose-dependent relationship, which is critical for the therapeutic potential of these compounds. These findings highlight the potential of PZ-9 and PZ-11 as anticancer agents, particularly given their ability to inhibit cell proliferation.

The consistent results from both MTT and xCELLigence RTCA analyses reinforce the robustness of these findings and suggest that PZ-9 and PZ-11 molecules warrant further investigation to elucidate their mechanisms of action and therapeutic potential in breast cancer treatment.

Particularly, the PZ-11 molecule demonstrated a higher antiproliferative effect with respect to PZ-9 at lower concentrations (15 μM % 48.25, 20 μM % 51.27) on the MCF-7 breast cancer cells, positioning it as a promising candidate for anticancer drug development. Thus, qRT-PCR analysis was continued with the PZ-11-treated and nontreated MCF-7 breast cancer cells in order to show the effect of the compound on gene expression level.

The downregulation of pro-apoptotic genes like *BAK1* and *BCL2L11* indicates a potential shift toward reduced apoptotic signaling. *BAK1* is known to promote mitochondrial outer membrane permeabilization, a critical step in apoptosis, while *BCL2L11* (also known as BIM) is a pro-apoptotic member of the Bcl-2 family that facilitates apoptosis in response to various stress signals.^38,39^ According to qRT-PCR results, the decrease in these genes could imply that PZ-11 may inhibit apoptosis in MCF-7 breast cancer cells, potentially allowing for enhanced cell survival under certain conditions.

Conversely, the downregulation of antiapoptotic genes such as *BAG3* and *BIRC3* suggests that PZ-11 may also be promoting apoptosis through the inhibition of survival pathways. BAG3 is involved in cellular stress responses and has been shown to stabilize antiapoptotic proteins, thereby contributing to cell survival.^40,41^ Its downregulation could sensitize cells to apoptotic signals, indicating that PZ-11 might be acting as an apoptosis-inducing agent by disrupting the balance between pro- and antiapoptotic factors. The downregulation of *TNF* and its receptor TNFRSF10A (also known as TRAIL receptor 1) suggests a potential reduction in inflammation-related signaling pathways, which can influence tumor progression and response to therapy.^42^ This could indicate that PZ-11 not only affects apoptotic pathways but also modulates the tumor microenvironment, potentially reducing the inflammatory signals that can promote cancer cell survival and proliferation. The observed changes in gene expression highlight the complexity of the cellular response to PZ-11 and suggest that its mechanism of action may involve multiple pathways, including apoptosis, survival signaling, and inflammation. This multifaceted impact could contribute to the overall therapeutic effect of PZ-11 in treating breast cancer, particularly in overcoming resistance mechanisms often seen in MCF-7 breast cancer cells.^43^ The BCL-2 family of proteins, which includes both pro-apoptotic (BAD, HRK) and antiapoptotic members (BCL2A1) plays a crucial role in regulating apoptosis. The upregulation of *BAD* and *HRK* suggests a shift toward promoting apoptosis in response to PZ-11 treatment as these proteins can inhibit the function of antiapoptotic proteins like BCL2A1 thereby facilitating cell death.^44,45^ The balance between these opposing forces is critical in determining the fate of cancer cells, and the observed changes indicate that PZ-11 may induce a pro-apoptotic environment within the MCF-7 breast cancer cells.

The upregulation of *CASP10* and *CASP14*, both of which are caspases involved in the apoptotic pathway, further supports the notion that PZ-11 promotes apoptosis in MCF-7 breast cancer cells. *CASP10* is known to initiate the extrinsic apoptotic pathway, while *CASP14* has been implicated in the execution phase of apoptosis.^46,47^ The expression of *BNIP3L*, which is associated with autophagy and apoptosis, also indicates a potential mechanism through which PZ-11 exerts its effects. *BNIP3L* can promote cell death under certain conditions, particularly in the context of hypoxia or stress, which may be relevant in the tumor microenvironment.^48,49^ Additionally, the upregulation of *IGF1R* and *TNFRSF11B* (also known as *RANK*) suggests that PZ-11 may also influence survival signaling pathways. *IGF1R* is known to promote cell survival and proliferation, while *TNFRSF11B* is involved in osteoclast differentiation and survival, indicating that PZ-11 may elicit a complex response that includes both pro-apoptotic and survival signals.^48,49^ The interplay between these pathways could be critical in determining the overall response of MCF-7 breast cancer cells to PZ-11.

On the other hand, treatment of MCF-7 breast cancer cells with the synthesized TZD derivative resulted in a significant downregulation of *AIFM1* (apoptosis-inducing factor mitochondria-associated 1) gene expression, with approximately a 2.5-fold decrease compared to the nontreated control group. *AIFM1* is a key regulator of caspase-independent apoptosis and is typically translocated from the mitochondria to the nucleus during cellular stress, where it contributes to chromatin condensation and large-scale DNA fragmentation.^50,51^ The TZD compound may be influencing cell death pathways apart from the AIF-mediated apoptotic pathway, as indicated by the observed decrease in *AIFM1* expression. In line with earlier research showing that specific TZD derivatives could affect mitochondrial integrity and stimulate cytochrome C release, resulting in classical caspase cascade activation, this finding suggests a possible shift toward caspase-dependent apoptosis or other non-AIF-regulated mechanisms.^52^ As a result, the inhibition of *AIFM1* expression could indicate a mechanistic departure from noncanonical apoptosis and lend support to the idea that TZDs cause cytotoxicity via a variety of apoptotic pathways, some of which may be PPAR-γ-independent. In addition, disruption of microtubule integrity is associated with altered signaling pathways that can enhance apoptosis, potentially through *AIFM1* downregulation.^53^

In our study, PZ-11 coupled with downregulation of antiapoptotic genes (*AIFM1, BAG3, BIRC3*) and upregulation of pro-apoptotic markers (*BAD, HRK, CASP10, CASP14*). Also, studies of thiazolidinediones have shown they can trigger apoptosis via suppression of *Bcl-2/Bcl-xL* and activation of caspase pathways, independently of PPAR-γ activation, suggesting that antiapoptotic family member downregulation is a recurrent and relevant mechanism in this class of compounds.^53^

Deng S *et al.* showed that a hybrid molecule (EP-TZD derivative, labeled compound 13o) had an IC_50_ ∼ 3.06 μM against MCF-7 cells, inhibited cell cycle at G0/G1, altered mitochondrial membrane potential, increased ROS, and induced apoptosis via inhibition of the PI3K/Akt/mTOR pathway.^54^ This is highly relevant to our work as PZ-11 also seems to impact apoptotic gene expression, and modeling suggests it binds apoptotic regulator *AIFM1*. While the mechanisms are different *(PI3K*/Akt/mTOR vs *AIFM1*/caspases, etc.), both approaches achieve apoptosis and show that TZD-hybrids can modulate intrinsic death pathways.

Moreover, more general classes of TZD derivatives have previously been shown to modulate apoptotic and cell-cycle-regulatory genes in breast cancer cell lines. TZDs like troglitazone, rosiglitazone, and pioglitazone induce apoptosis and growth arrest, in part by reducing antiapoptotic Bcl-2/Bcl-xL expression and increasing expression of pro-apoptotic proteins, including BAD, as well as enhancing caspase activation.^55^

In addition, breast cancer cells with TZD ligands showed suppression of *survivin* (an inhibitor of apoptosis) and upregulation of *BAX and BAD*, aligning with the axis of apoptotic commitment our PZ-11 appears to influence.^55^

The changes in the expression levels of these critical genes involved in the apoptotic pathway in MCF-7 cells following PZ-11 treatment suggest a potential shift toward apoptosis and reduced cell survival, indicating a promising avenue for further research into the therapeutic applications of PZ-11 in breast cancer treatment.

Overall, the study underscores the significance of PZ-11 as a potential target for novel breast cancer therapies, offering a promising avenue for enhancing the efficacy of current treatment regimens. The SAR observed in this study indicates that specific modifications in the chemical structures of the compounds can lead to enhanced biological activity. This is consistent with findings from other research that highlights the importance of structural diversity in developing effective tubulin inhibitors.^35,36^

The synthesized PZ compounds represent a promising class of anticancer agents targeting tubulin polymerization. The significant cytotoxicity exhibited by compound PZ-11 warrants further investigation, including *in vivo* studies and exploration of their mechanisms of action. The integration of molecular hybridization with a focus on tubulin destabilization offers a viable strategy for the development of new therapeutic options in cancer treatment. Future research should aim to optimize this compound and assess its efficacy in more complex biological systems, ultimately contributing to the advancement of cancer therapeutics.

### 3.4 Molecular Docking

The molecular docking analysis was designed and executed based on the findings derived from the gene expression profiling of a selected panel of genes. These genes were specifically chosen due to their known involvement in key cellular processes, including microtubule organization, apoptosis, regulation of the cell cycle, tumor suppression mechanisms, and cellular proliferation. Out of the total 88 genes analyzed, the most significant expression changes were observed in genes encoding critical regulatory proteins such as tubulin polymerase, *AIFM1, DAPK1, TNF*, and *IGF-1R*. These genes were identified as central players in the biological pathways under investigation. Prior to the evaluation of the binding interactions of compound PZ-11 with the protein products of these genes, validation of target structures was performed to ensure the relevance and accuracy of the docking study. The validation process was performed according to the method in Section 2.7.1. The calculated RMSD values were 1.81, 0.93, 0.17, 1.85, and 0.92 for 5J2T, 4LII, 9INV, 2AZ5, and 2AZ5, respectively, whereas the threshold value is 2.00 for a reliable docking process. Accordingly, this result allows us to move on to further molecular docking studies. Therefore, exploratory docking was made for these proteins; however, only the analysis involving apoptosis-inducing factor (AIF), the protein encoded by the *AIFM1* gene, yielded reliable results. It is worthy to mention that there is a geometric isomerism in our structures due to the methylene bridge (Figure 2), and thus the E and Z isomers of PZ-11 were examined separately in docking studies. The two isomers showed high affinity toward AIF and exhibited very close docking scores (−6.8 kcal/mol for Z isomer and −6.2 kcal/mol for E isomer). Coming to their interaction profile with AIF, the presence of the nitro group helped to carry out a salt bridge with cationic quaternary amine in Lys-286; besides that, the carbonyl group of the acetophenone moiety accepted 2 hydrogen bonds from Arg-172 and Arg-285. However, the Z isomers differed by accepting 2 more H-bonds from Arg-172 and Asp-438 by the ketones of the TZD ring, suggesting that geometric isomerism can markedly influence hydrogen-bonding patterns and binding strength (Figure 8). In addition to ionic bonds and H-bonds, many other hydrophobic interactions were also observed.

**Figure 8:**
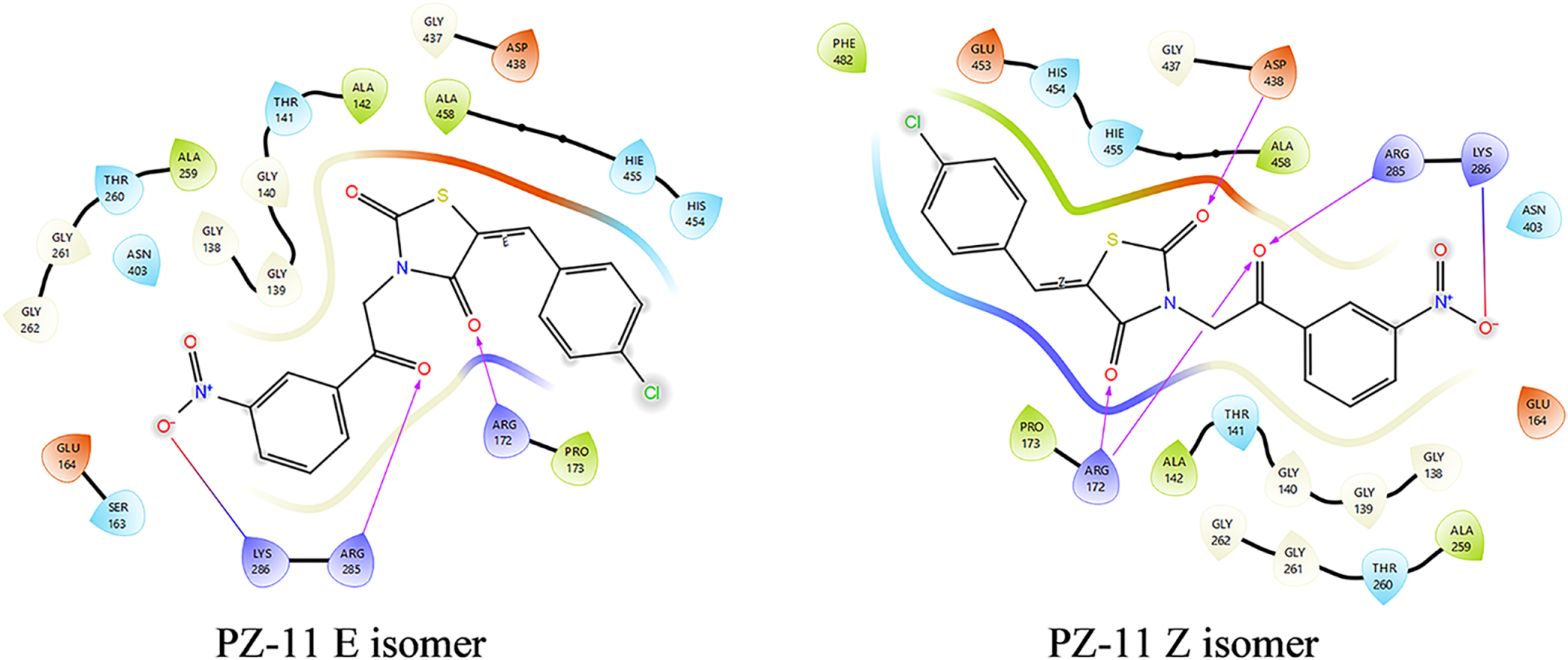
2D diagrams of protein–ligand interactions of compound PZ-11 isomers in the AIF-binding site.

The residue numbering in this study is based on the AIF structure (PDB ID: 4LII, chain A). Although the residues identified in the docking analysis (Arg-172, Arg-285, Lys-286, and Asp-438) correspond to this specific structural numbering, their spatial positions align with the FAD/NAD-binding and redox-active regions of the full-length protein, as previously described by Brosey *et al.* and Sevrioukova. In those studies, residues such as Pro-173, Lys-177, Phe-310, Glu-314, Arg-430, and His-454 were highlighted as residues that stabilize the FAD+ cofactor near the NADH-binding site and contribute to ligand stabilization and redox function.^56,57^ The roles of Arg-172 and Arg-285 in forming hydrogen bonds and stabilizing polar groups are consistent with their location near the NADH-binding pocket, while Lys-286 likely contributes to electrostatic interactions within the FAD-binding site. In addition, hydrogen bonding with acidic residues like Asp-438 supports earlier findings by Russo *et al.* and Doti *et al.*, who reported that similar polar interactions improved peptide binding on the AIF protein surface.^58,59^ Overall, our docking results confirm that electrostatic interactions (e.g., nitro–Lys bridging) and carbonyl-centered hydrogen bonding are key to ligand binding and stability in AIF, providing a rational basis for future optimization of AIF-binding molecules.

### 3.5 Molecular Dynamics Simulations

The RMSD parameter provides important information about the structural conformation of the protein–ligand complex.^60^ The stability of compound PZ-11 within the AIF (*AIFM1*) active site was assessed by analyzing the RMSD profiles obtained from molecular dynamics simulation. The smallest value of RMSD indicates the good stability of the structure. Figure 9A shows the RMSD (nm) versus time (ns) plot for the AIF-ligand complex for the 200 ns simulation. In the first 10 ns, there was a slight increase by 0.1 nm in the RMSD values; after that, a stable plateau of RMSD values was observed. This may indicate that it took the AIF-ligand complex around 10 ns to settle down, reaching a stabilization state. The RMSD values remained below the 0.4 nm indicating a very stable ligand–protein interactions since the threshold value for RSMD in some studies is 0.4 nm^61^ and in other studies is 0.6 nm.^62^ Overall, the RMSD results show that the MD trajectories were relatively stable and were within an acceptable range.

**Figure 9:**
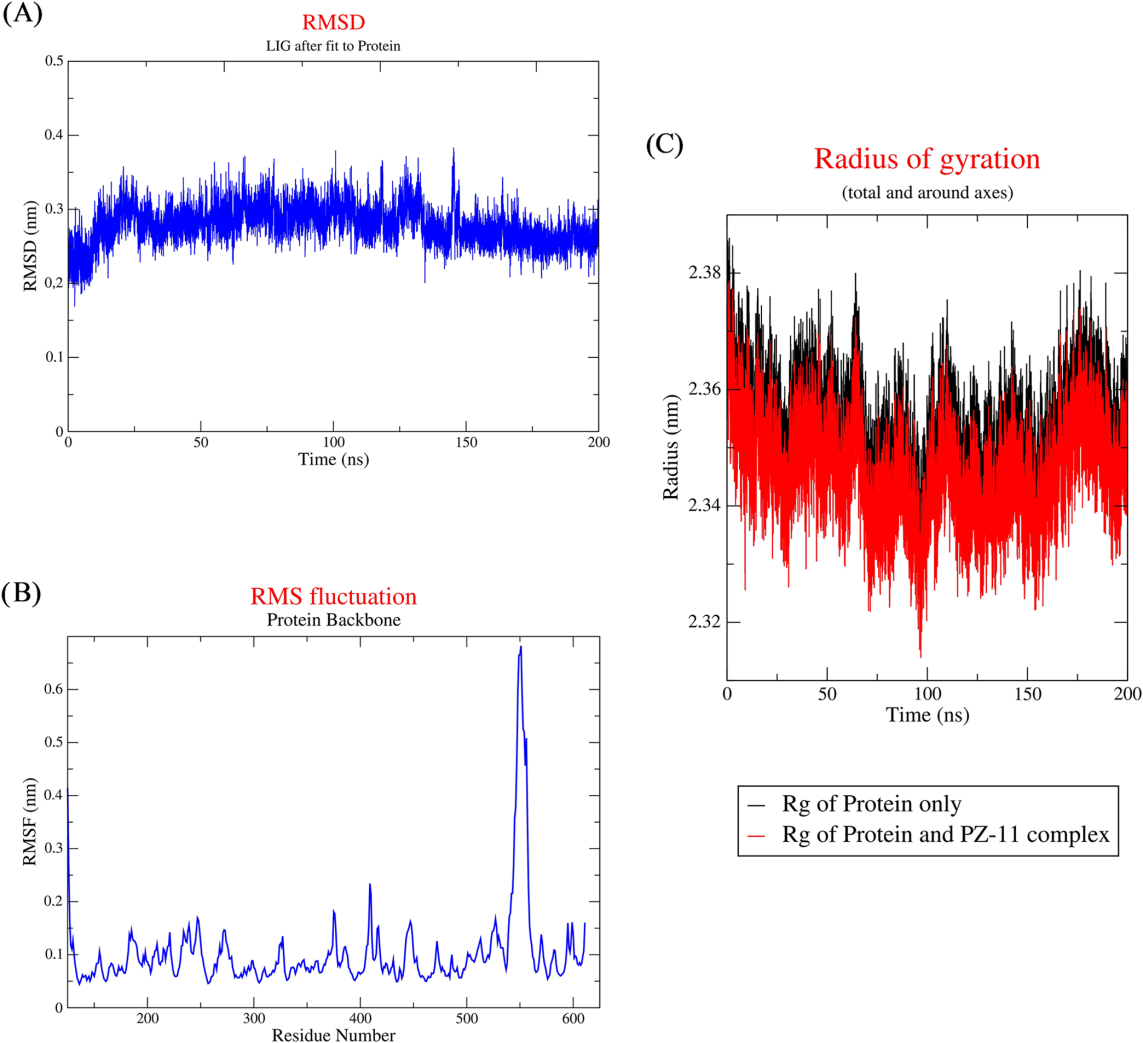
Molecular dynamics simulation trajectory analysis of the AIF with PZ-11. (A) RMSD plot showing the stability of compound PZ-11 in the AIF active site. (B) RMSF plot illustrating the flexibility of each residue in the AIF structure during the MD. The RMSF plot starts at residue 125 to match the original numbering in the 4LII PDB file. (C) Rg plot of original AIF and AIF-PZ11 complex.

Conformational changes in the AIF residues involved in the AIF–PZ11 complex interactions were assessed through RMSF analysis, which provided insights into the flexibility and dynamic fluctuations of individual residues within the complex.^61^ A high RMSF value indicates increased residue flexibility and reduced structural stability during the MD simulation, whereas a lower RMSF value reflects limited flexibility and greater conformational stability of the system.^63^ Figure 9B presents the RMSF profile for the AIF backbone; this analysis is crucial for evaluating residue-level flexibility following ligand binding. Notably, there was one distinct peak in the RMSF plot, which indicates a region of increased mobility, potentially reflecting functionally relevant dynamics or structural adaptations following ligand accommodation.

The deep analysis of RMSF values revealed that the residue exhibiting the highest degree of atomic fluctuation (Pro-551, RMSF = 0.7304 nm) was located at a considerable distance from the ligand-binding site. In contrast, the residues directly involved in key interactions with PZ-11 displayed the lowest RMSF values (0.0624, 0.0701, and 0.0585 nm for Thr-141, Arg-172, and Arg-285, respectively). This observation suggests that the binding site region remains structurally stable throughout the molecular dynamics (MD) simulation with minimal positional deviation of the atoms involved in ligand recognition and binding. Such low fluctuations in the active site residues indicate a well-defined and rigid interaction interface, which is typically associated with strong and persistent ligand-protein binding. Furthermore, the overall RMSF profile across the entire protein structure remained relatively low (below 0.02 nm), underscoring the global conformational stability of the protein–ligand complex over the simulation.^64^

The radius of gyration (Rg) serves as a key indicator of the structural compactness of a protein, both in its free form and when bound to a ligand. Throughout MD simulations, Rg is commonly employed to assess whether the complex maintains a stably folded conformation or undergoes unfolding. Decreased Rg in complex indicates a more compact structure upon ligand binding, while increased Rg in complex indicates a possible domain separation, loop flexibility, or unfolding.^65,66^ The average Rg obtained for the AIF-PZ11 complex was lower than the Rg value obtained for the AIF protein only, which means that the binding of PZ-11 led to a 0.02 nm decrease in Rg, signifying a more compact conformation of the protein structure upon ligand binding (Figure 9C).

Hydrogen-bond interactions, crucial for determining the specificity and affinity of ligand binding, were quantitatively depicted in Figure 10. Herein Figure 10A, the maximum number of hydrogen bonds versus time for the AIF-PZ11 complex during a 200 ns MD simulation was illustrated. The result shows the appearance of a maximum of six H-bond interactions between PZ-11 and AIF. Compound PZ-11 consistently forms at least two or three hydrogen bonds throughout the simulation, suggesting a potentially strong and stable engagement with the active site. To investigate whether water molecules participate in hydrogen bonding with the compound PZ-11, we also analyzed the hydrogen-bond interactions between PZ-11 and the surrounding water molecules. Figure 10B shows a maximum of seven hydrogen bonds formed between PZ-11 and water molecules. The results further indicate that at least two consistent hydrogen bonds were maintained between PZ-11 and one or two water molecules. This highlights the importance of considering the role of water molecules in shaping the ligand’s interaction profile within the binding site.

**Figure 10:**
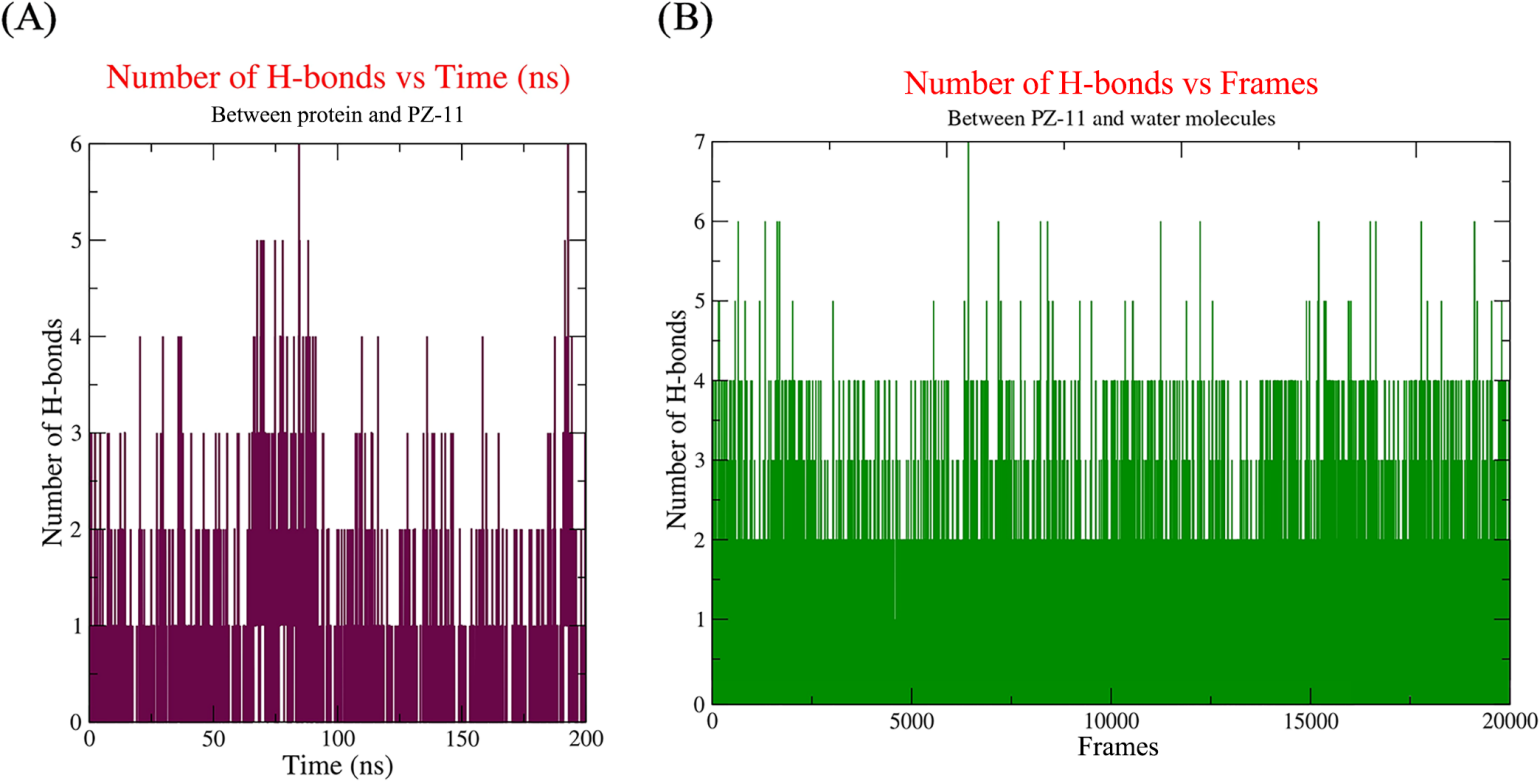
Simulation result showing the number of H-bonds for (A) AIF-PZ11 complexes and (B) PZ-11 and water molecules (100 frames per 1 ns).

Based on the molecular dynamics’ findings, Figure 11 provides 3D representations of the binding conformations of compound PZ-11 within the AIF active site after 50, 100, and 190 ns. It illustrates the detailed interaction network formed by compound PZ-11.

**Figure 11:**
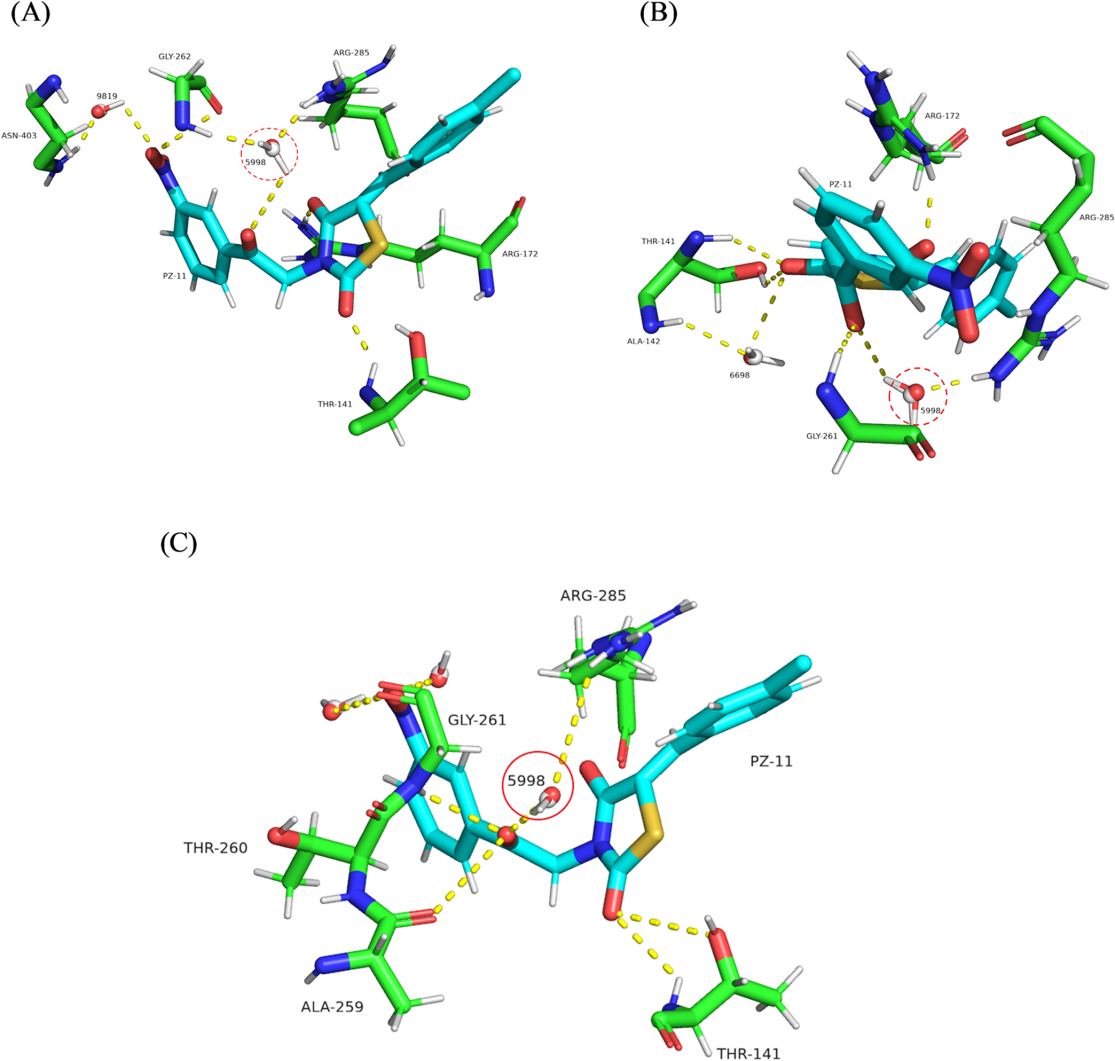
Binding profiles of protein–ligand interactions of compound PZ-11 in the AIF active pocket after (A) 50 ns, (B) 100 ns, and (C) 190 ns.

From Figure 11A–C we could observe that PZ-11 maintained H-bond interactions with four key residues (Thr-141, Arg-172, Gly-261, and Arg-285) and also a water molecule numbered 5998 coordinated H-bonds between PZ-11 and Arg-285 in the investigated frames. The observation of a conserved water molecule mediating a hydrogen bond between the ligand and the same protein residue across multiple simulation frames suggests a stable, water-bridged interaction. Such interactions are known to contribute significantly to ligand binding affinity and overall complex stability, especially when direct hydrogen bonding is sterically hindered. The recurrence of this water-mediated bridge suggests that the water molecule may be structurally conserved and potentially integral to the binding mechanism, acting as an extension of the protein–ligand interface.

### 3.8 ADME Estimation

Among the synthesized compounds, the cytotoxicity analysis indicates that PZ-9 and PZ-11 were the most cytotoxic against MCF-7 breast cancer cells compared to vincristine. The drug-likeness of compounds PZ-9 and PZ-11 was compared with the commercial drug Vincristine in terms of physicochemical parameters and ADME determination. Thus, both PZ-9 and PZ-11 have passed Lipinski’s rule of five^67^ with zero violation, as well as Vincristine (Table 4). Compound PZ-11 showed a lower consensus Log P (2.73), while PZ-9 showed a consensus Log P (3.50), which is very close to Vincristine (3.41). Both compounds did not exhibit permeability for the blood-brain barrier (BBB) and thus theoretically should have no side effects on the central nervous system (CNS). Both compounds were not considered as P-glycoprotein (P-gp) substrates, therefore they may exhibit long-acting activity in the body.

**Table 4:** SwissADME-Predicted Physicochemical and Drug-likeness Properties of Compounds PZ1–11

**Compound #**	**Substrate to P-gp**	**GI absorption**	**BBB permeation**	**CYP450 inhibition** **(out of 5)**	**Consensus log Po/w**	**TPSA (Å** ^**2**^ **)**	**Number of rotatable bonds**	**Lipinski** **violation #**	**Veber** **violation #**
PZ-1	no	high	no	3	2.93	107.44	7	0	0
PZ-2	no	high	no	3	3.15	107.44	7	0	0
PZ-3	no	high	no	3	3.23	107.44	7	0	0
PZ-4	no	low	no	5	2.20	153.26	8	0	1
PZ-5	no	high	no	3	2.94	116.67	8	0	0
PZ-6	no	high	no	3	3.54	107.44	7	0	0
PZ-7	no	high	no	3	3.93	107.44	7	0	0
PZ-8	no	high	no	4	3.81	79.75	4	0	0
PZ-9**	no	high	no	4	3.50	88.98	5	0	0
PZ-10	no	high	no	4	4.53	79.75	4	0	0
PZ-11*	no	high	no	3	2.73	125.57	5	0	0

Cytochrome P450 (CYP450) enzyme inhibition was assessed to evaluate the potential for drug–drug interactions. Only compound PZ-4 achieved the highest inhibition score of 5, indicating that it may inhibit all five major CYP450 isoforms, CYP1A2, CYP2C19, CYP2C9, CYP2D6, and CYP3A4. This suggests that the derivative could pose a risk of multiple drug interactions.^68^

Overall, none of the synthesized compounds were identified as P-gp substrates, nor did they show permeability across the BBB. All compounds complied with Lipinski’s rule of five and demonstrated high predicted gastrointestinal absorption, except for compound PZ-4, which exhibited low absorption and one violation of Veber’s rule^69^ because of its high Topological Polar Surface Area (TPSA) (153.26 Å^2^). TPSA is defined as the sum of the surface areas of all polar atoms (usually O and N) in a molecule, calculated based on their topological (2D) structure rather than 3D coordinates. Compounds with a TPSA ≤ 140 Å^2^ tend to have good intestinal absorption, while compounds with a TPSA ≤ 90 Å^2^ are more likely to cross the BBB. TPSA is considered a good predictor of cell membrane permeability as compounds having TPSA higher than 140–150 Å^2^ tends to have low permeability into cell membrane.^70,71^

### 3.9 *In Silico* Toxicity Assessment

The metabolic profiling and structural alert analysis of the PZ compound series reveal generally consistent biotransformation patterns across the set, with Phase I metabolites ranging from 8 to 10 and Phase II metabolites ranging from 6 to 10. This indicates moderate metabolic stability and comparable metabolic pathways among the compounds. Regarding structural alerts, compounds (PZ-1, PZ-5, PZ-7, and PZ-9) show no alerts for either genotoxic or nongenotoxic carcinogenicity, suggesting a favorable safety profile. However, a subset of compounds—namely PZ-2, PZ-3, PZ-6, PZ-8, and PZ-10—exhibit 1 alert for potential nongenotoxic carcinogenicity, and only two compounds (PZ-4 and PZ-11) display 1 genotoxic alert warranting further toxicological evaluation. Overall, the PZ series demonstrates consistent metabolic characteristics with generally low structural concern, supporting their continued investigation and optimization (Table 5).

**Table 5:** Predicted Possible Phase I Metabolites, Phase II Metabolites, Genotoxic Carcinogenic and Nongenotoxic Carcinogenic Properties of Compounds PZ1–11

**Compound #**	**Total metabolites number in** **Phase I**	**Total metabolites number in** **Phase II**	**Structural alerts for genotoxic carcinogenicity**	**Structural alerts for nongenotoxic carcinogenicity**
PZ-1	8	6	no alert	no alert
PZ-2	10	9	no alert	1 alert
PZ-3	8	6	no alert	1 alert
PZ-4	8	6	1 alert	no alert
PZ-5	9	7	no alert	no alert
PZ-6	8	6	no alert	1 alert
PZ-7	9	7	no alert	no alert
PZ-8	8	7	no alert	1 alert
PZ-9**	9	7	no alert	no alert
PZ-10	8	10	no alert	1 alert
PZ-11*	8	6	1 alert	no alert

## 4 Conclusions

This study demonstrated that the synthesized thiazolidinedione derivatives, particularly PZ-9 and PZ-11, exhibit significant cytotoxic and antiproliferative effects against MCF-7 breast cancer cells, with PZ-11 showing the most promising activity in both MTT and xCELLigence RTCA assays. The superior efficacy of PZ-11 at lower concentrations compared to vincristine highlights its potential as a lead compound for breast cancer therapy. Gene expression analysis revealed that PZ-11 downregulates antiapoptotic genes (*AIFM1*, *BAG3*, *BIRC3*) and upregulates pro-apoptotic markers (*BAD*, *HRK*, *CASP10*), suggesting a dual mechanism involving microtubule disruption and caspase-dependent apoptosis. Molecular docking results with AIF confirm that electrostatic interactions within the FAD-binding site and hydrogen-bonding interactions within the NADH-binding pocket play key roles in ligand binding and stability in AIF. These findings suggest that compounds containing negatively charged groups capable of forming salt bridges (such as nitro–Lys interactions) and carbonyl groups that serve as hydrogen-bond acceptors are important for enhancing ligand affinity toward AIF. The molecular dynamics results further supported the interaction between PZ-11 and the AIF protein, confirming the structural stability and binding affinity of the complex throughout the 200 ns simulation. It is demonstrated by ADMET predictions that PZ compounds possess suitable pharmacokinetic properties. These findings underscore the therapeutic potential of PZ-11 as a multitarget anticancer agent.

The strategic incorporation of microtubule-targeting structural motifs into the TZD scaffold in the design of PZ compounds aligns with emerging trends in anticancer drug development, where hybrid molecules integrating multiple pharmacophores demonstrate enhanced antiproliferative activity and improved modulation of cancer-associated pathways such as cell cycle regulation and apoptosis. While numerous studies have reported the anticancer potential of TZD derivatives, few have explicitly explored the rational fusion of TZDs with microtubule-disrupting moieties. In this context, PZ-11 may represent a novel direction within TZD-based drug design, offering a promising dual-targeting approach that warrants further investigation.

What distinguishes our findings is the broader apoptotic gene signature elicited by PZ-11 treatment, which involves the modulation of both intrinsic and extrinsic apoptosis pathways. Notably, the upregulation of *CASP14* and *CASP10*, along with the downregulation of antiapoptotic genes such as *BIRC3* and *AIFM1*, suggests a multifaceted mechanism of apoptosis induction. *BIRC3*, a member of the inhibitor of the apoptosis protein (IAP) family, is frequently overexpressed in cancer cells and contributes to apoptosis resistance. Its suppression by PZ-11 may therefore enhance susceptibility to programmed cell death. Likewise, *AIFM1* (apoptosis-inducing factor, mitochondria-associated 1), although generally pro-apoptotic under specific conditions, can also play roles in promoting cell survival by maintaining mitochondrial integrity. The significant downregulation of *AIFM1* observed in our study may reflect a disruption of mitochondrial homeostasis, tipping the balance toward cell death. These results indicate that PZ-11 exerts a more potent and complex pro-apoptotic effect compared to previously reported thiazolidinedione monomers or simple derivatives, which often modulate a narrower subset of apoptotic markers. The ability of PZ-11 to simultaneously target multiple apoptotic regulators may, therefore, underlie its superior antiproliferative activity in breast cancer cells.

In conclusion, our study shows the remarkable anticancer potential of new TZD-based compounds, especially PZ-11, which altered important apoptosis-related genes to exhibit strong pro-apoptotic and antiproliferative effects in breast cancer cells. Molecular modeling studies and ADMET analyses further support its potential as a lead compound for therapeutic development. Nonetheless, it is important to acknowledge a number of limitations. The *in vitro* results have not yet been confirmed in other cancer models or *in vivo* systems and are presently limited to the MCF-7 cell line. Furthermore, experimental confirmation of these pathways is still required, even if computational investigations offer insightful information about potential molecular targets and interactions. In order to determine the precise molecular targets of PZ-11, further research should include mechanistic studies, wider screening across a variety of cancer cell lines, and *in vivo* evaluation in pertinent breast cancer models.

## Supplementary Material



## Notes

Spectra (^1^H NMR, ^13^C NMR, MS) of all synthesized compounds (Figure S1–S33), and molecular docking validation poses of the originally bound co-ligand FAD in AIF (Figure S34) (PDF)
