# Supplementary material for: Design, Synthesis, Molecular Docking, Dynamics Simulation, and Biological Evaluation of Novel Thiazolidinedione Derivatives Against Breast Cancer with Apoptosis-Inducing Activity
Source: ACS Omega. 2025 Dec 23;11(1):889–908. doi: 10.1021/acsomega.5c07713 (PMC12809312; doi:10.1021/acsomega.5c07713)
Supplement: Supplementary file 1 [file ao5c07713_si_001.pdf]

(Supporting Information)

Design, Synthesis, Molecular Docking, Dynamics Simulation and Biological Evaluation of Novel Thiazolidinedione Derivatives Against Breast Cancer with Apoptosis-Inducing Activity

[1]Pouria Zarrin, [1,2]Sarah Gado, [1]Ali Farhang Boroujeni, [3]Ibrahim Gadaşlı, [3]Fatma Zeynep Bozkurt, [3]Demet Cansaran-Duman, [3]Pelin Mutlu, [1]Zeynep Ates-Alagoz.*

[1] Ankara University, Faculty of Pharmacy, Department of Pharmaceutical Chemistry, 06560, Ankara, Turkey

[2] Ankara University, Graduate School of Health Sciences, 06110, Ankara, Turkey

[3] Ankara University, Biotechnology Institute, Department of Biotechnology, 06135, Ankara, Turkey

*Corresponding author: Zeynep Ates-Alagoz

Ankara University, Faculty of Pharmacy, Department of Pharmaceutical Chemistry, 06560, Tandogan, Ankara, Turkey

Email: zates@pharmacy.ankara.edu.tr

Table of Contents

ESI-MS, ^{1}H NMR, and ^{13}C NMR spectra of compounds PZ1-PZ11 (S1-S33)....................S3

Figure S1. ^{1}H NMR spectrum of compound PZ-1....................S3

Figure S2. ^{13}C NMR spectrum of compound PZ-1....................S4

Figure S3. ESI-MS spectrum of compound PZ-1....................S5

Figure S4. ^{1}H NMR spectrum of compound PZ-2....................S6

Figure S5. ^{13}C NMR spectrum of compound PZ-2....................S7

Figure S6. ESI-MS spectrum of compound PZ-2....................S8

Figure S7. ^{1}H NMR spectrum of compound PZ-3....................S9

Figure S8. ^{13}C NMR spectrum of compound PZ-3....................S10

Figure S9. ESI-MS spectrum of compound PZ-3....................S11

Figure S10. ^{1}H NMR spectrum of compound PZ-4....................S12

Figure S11. ^{13}C NMR spectrum of compound PZ-4....................S13

Figure S12. ESI-MS spectrum of compound PZ-4....................S14

Figure S13. ^{1}H NMR spectrum of compound PZ-5....................S15

Figure S14. ^{13}C NMR spectrum of compound PZ-5....................S16

Figure S15. ESI-MS spectrum of compound PZ-5....................S17

Figure S16. ^{1}H NMR spectrum of compound PZ-6....................S18

Figure S17. ^{13}C NMR spectrum of compound PZ-6....................S19

Figure S18. ESI-MS spectrum of compound PZ-6....................S20

Figure S19. ^{1}H NMR spectrum of compound PZ-7....................S21

Figure S20. ^{13}C NMR spectrum of compound PZ-7....................S22

Figure S21. ESI-MS spectrum of compound PZ-7....................S23

Figure S22. ^{1}H NMR spectrum of compound PZ-8....................S24

Figure S23. ^{13}C NMR spectrum of compound PZ-8....................S25

Figure S24. ESI-MS spectrum of compound PZ-8....................S26

Figure S25. ^{1}H NMR spectrum of compound PZ-9....................S27

Figure S26. ^{13}C NMR spectrum of compound PZ-9....................S28

Figure S27. ESI-MS spectrum of compound PZ-9....................S29

Figure S28. ^{1}H NMR spectrum of compound PZ-10....................S30

Figure S29. ^{13}C NMR spectrum of compound PZ-10....................S31

Figure S30. ESI-MS spectrum of compound PZ-10....................S32

Figure S31. ^{1}H NMR spectrum of compound PZ-11....................S33

Figure S32. ^{13}C NMR spectrum of compound PZ-11....................S34

Figure S33. ESI-MS spectrum of compound PZ-11....................S35

Figure S34. Alignment of AIF co-ligand FAD (Cyan color) and its re-docked conformer (Green)....................S36

1. ESI-MS, ^{1}H NMR, and ^{13}C NMR spectra of compounds PZ1-PZ11 (S1-S33)

(E)-3-(2-oxo-2-phenylethyl)-5-(3,4,5-trimethoxybenzylidene)thiazolidine-2,4-dione (PZ-1)

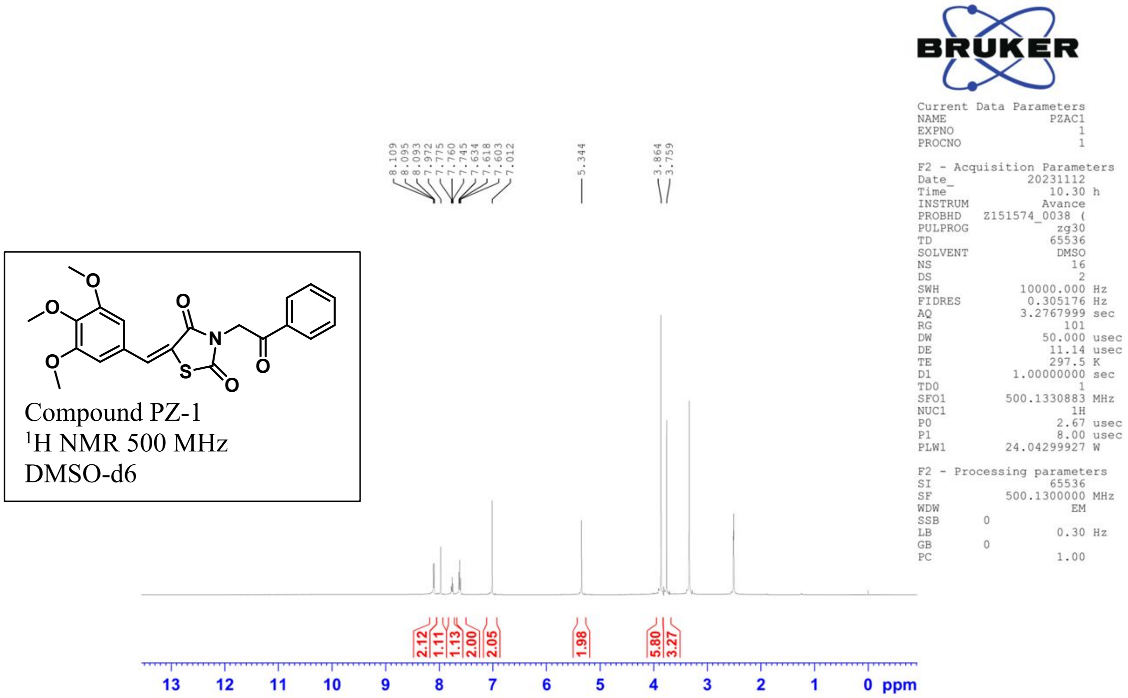

Figure S1. ^{1}H NMR spectrum of compound PZ-1

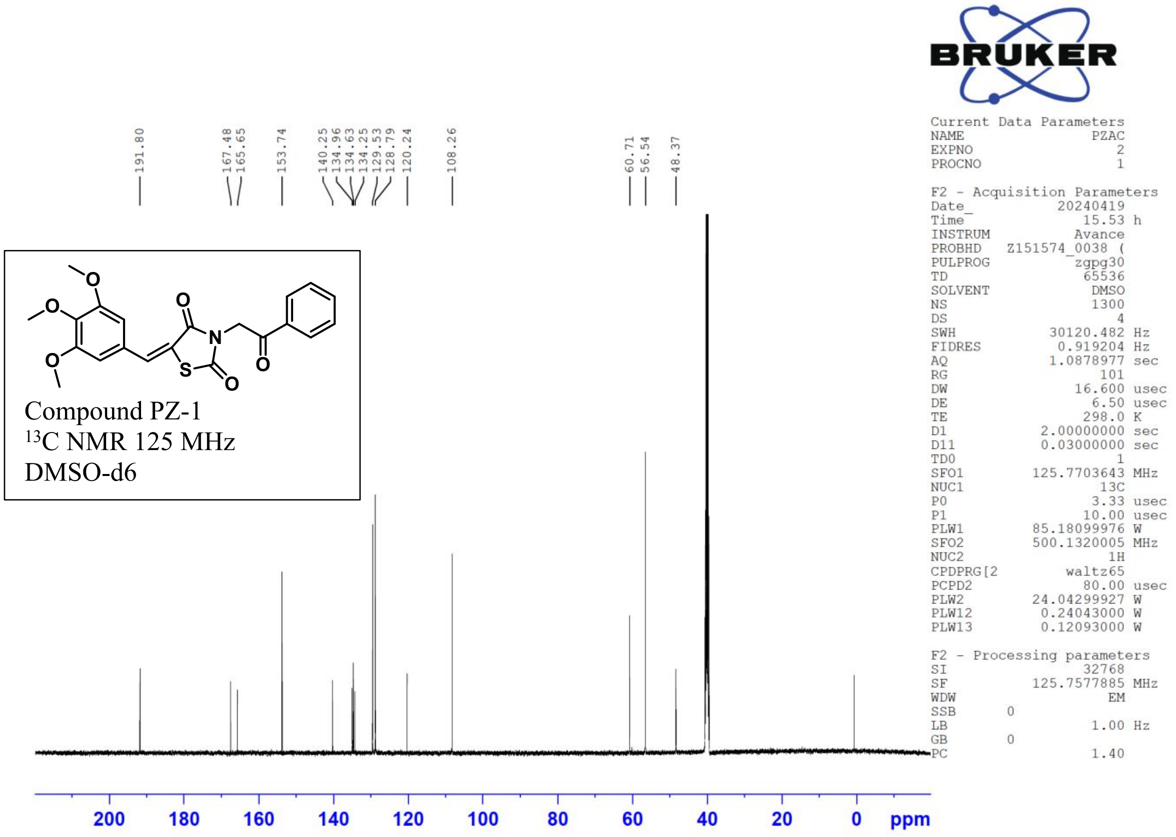

Figure S2. ^{13}C NMR spectrum of compound PZ-1

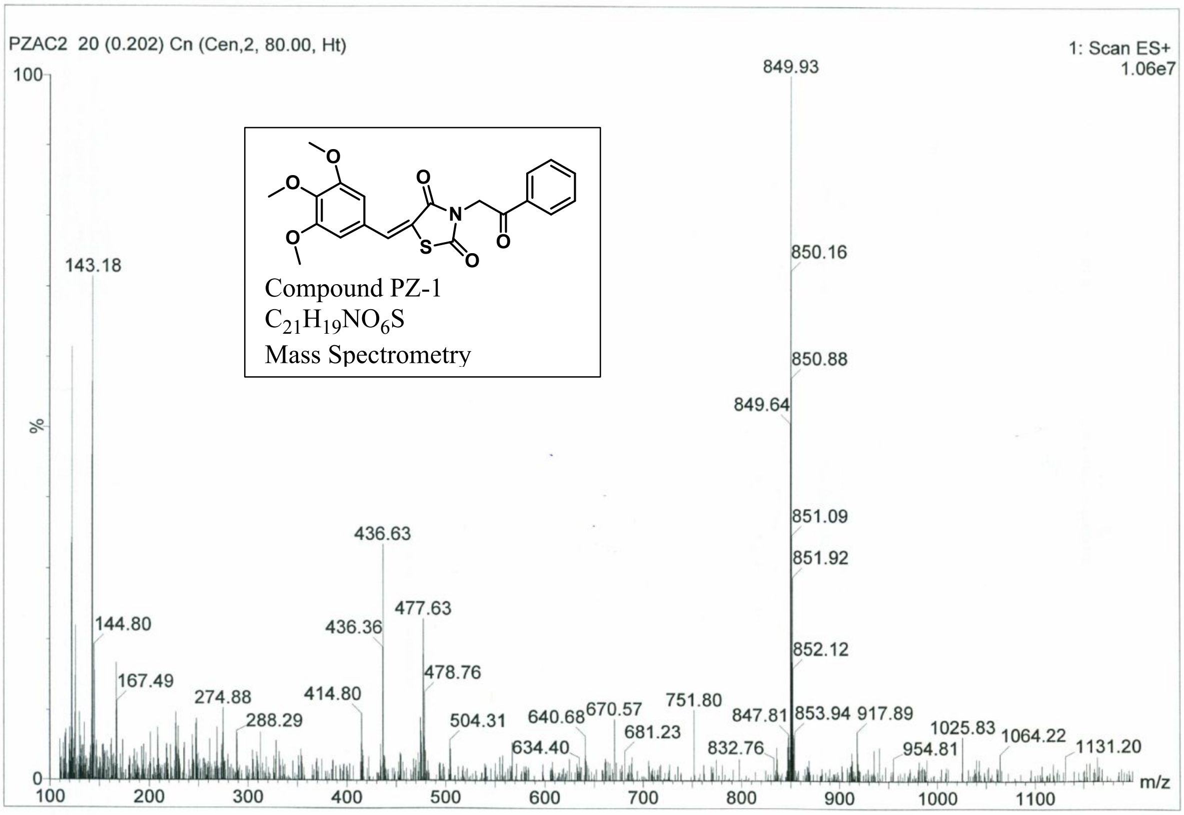

Figure S3. ESI-MS spectrum of compound PZ-1

(E)-3-(2-(2-fluorophenyl)-2-oxoethyl)-5-(3,4,5-trimethoxybenzylidene)thiazolidine-2,4-dione (PZ-2)

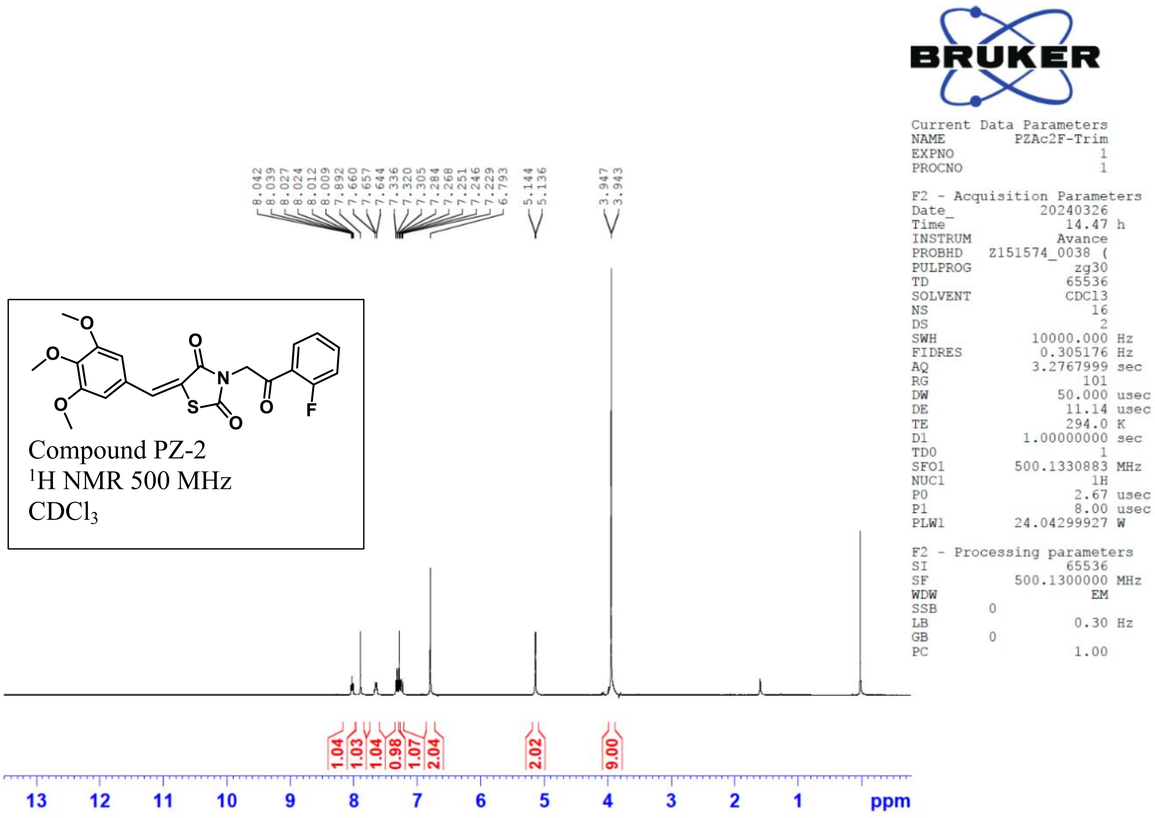

Figure S4. ^{1}H NMR spectrum of compound PZ-2

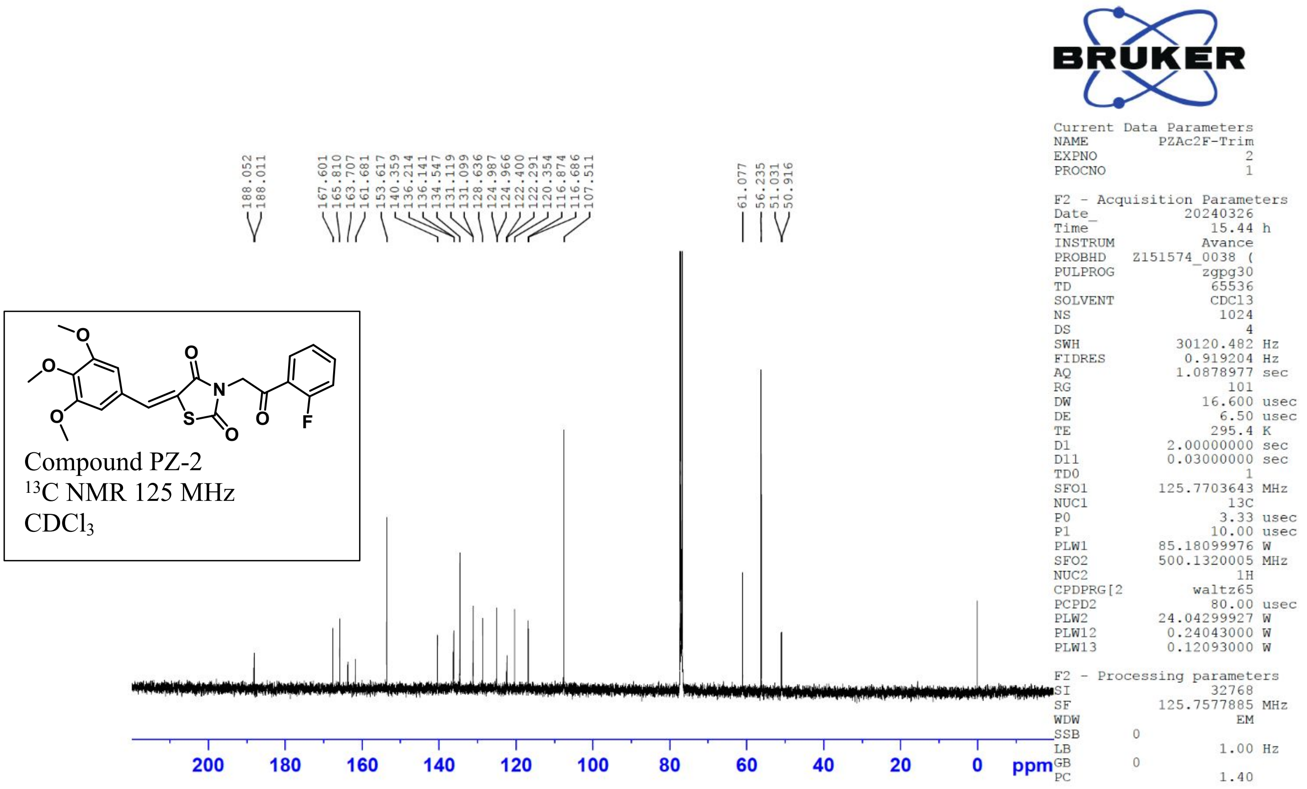

Figure S5. ^{13}C NMR spectrum of compound PZ-2

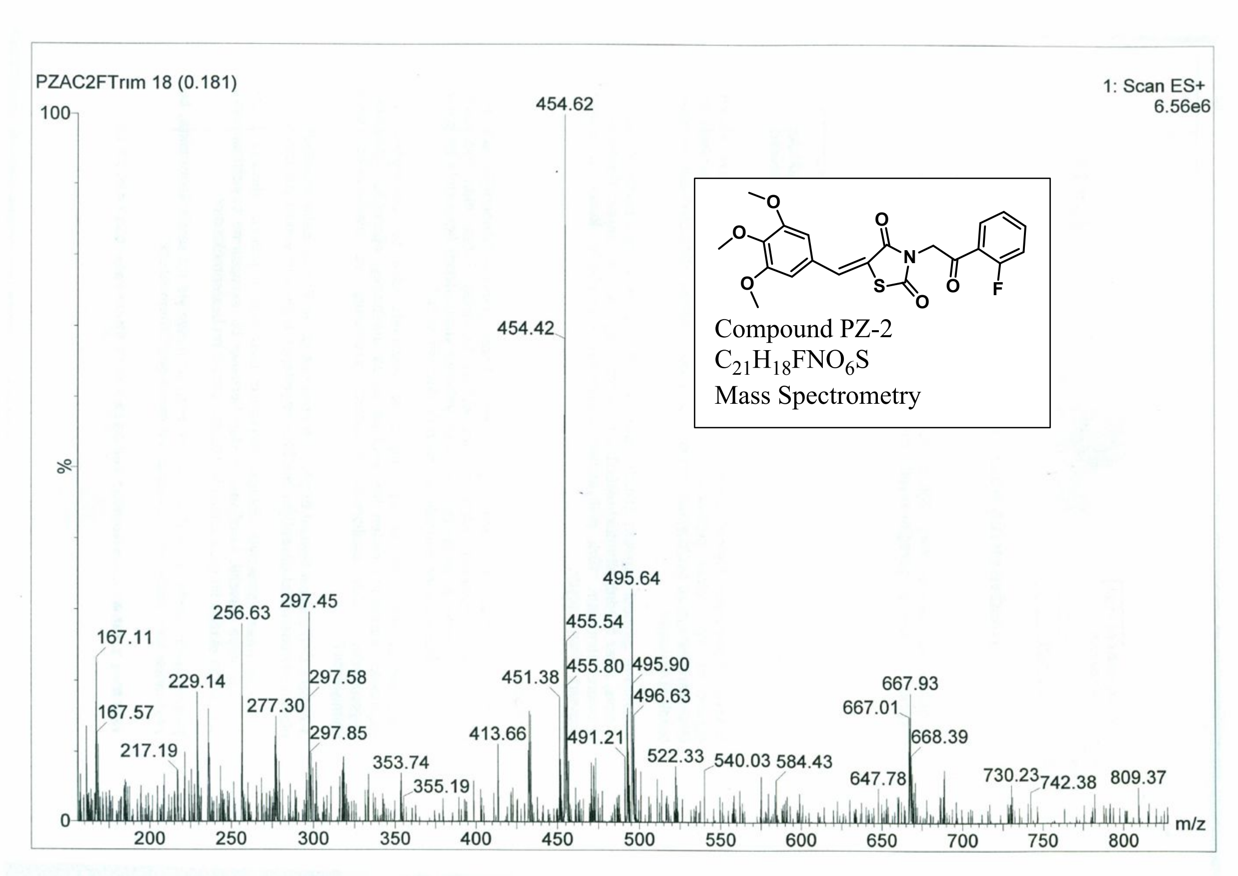

Figure S6. ESI-MS spectrum of compound PZ-2

(E)-3-(2-(4-fluorophenyl)-2-oxoethyl)-5-(3,4,5-trimethoxybenzylidene)thiazolidine-2,4-dione (PZ-3)

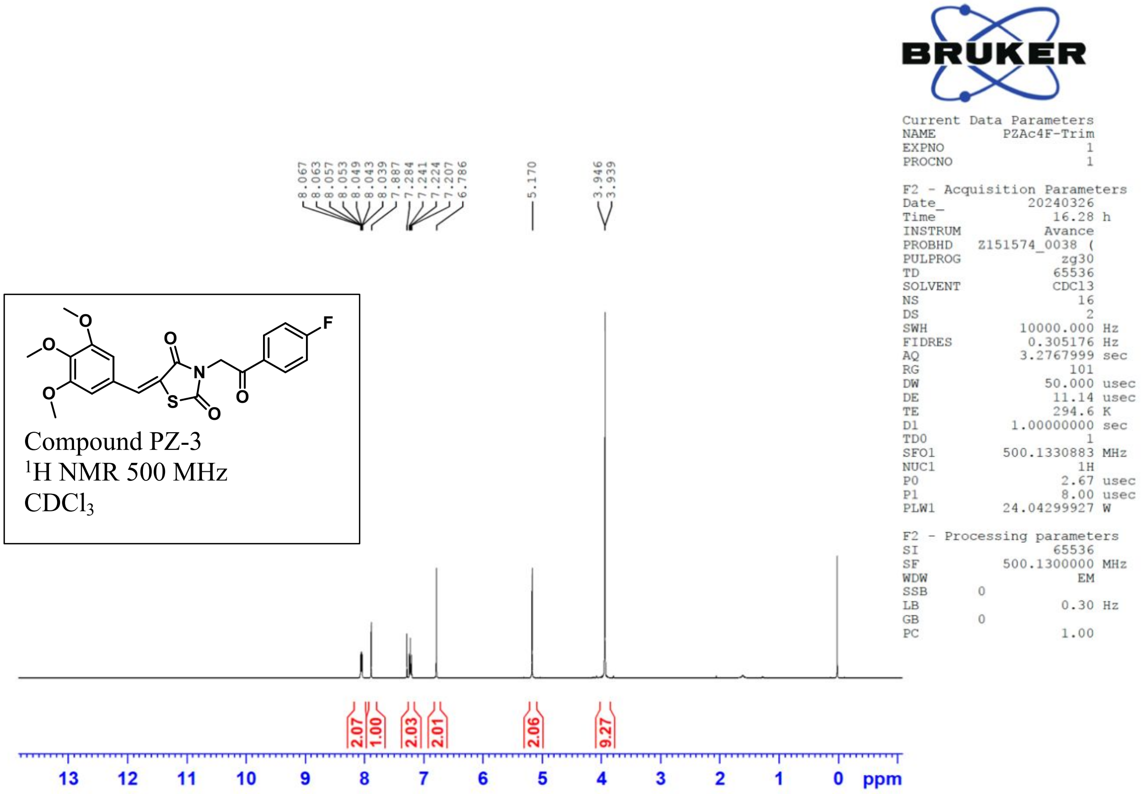

Figure S7. ^{1}H NMR spectrum of compound PZ-3

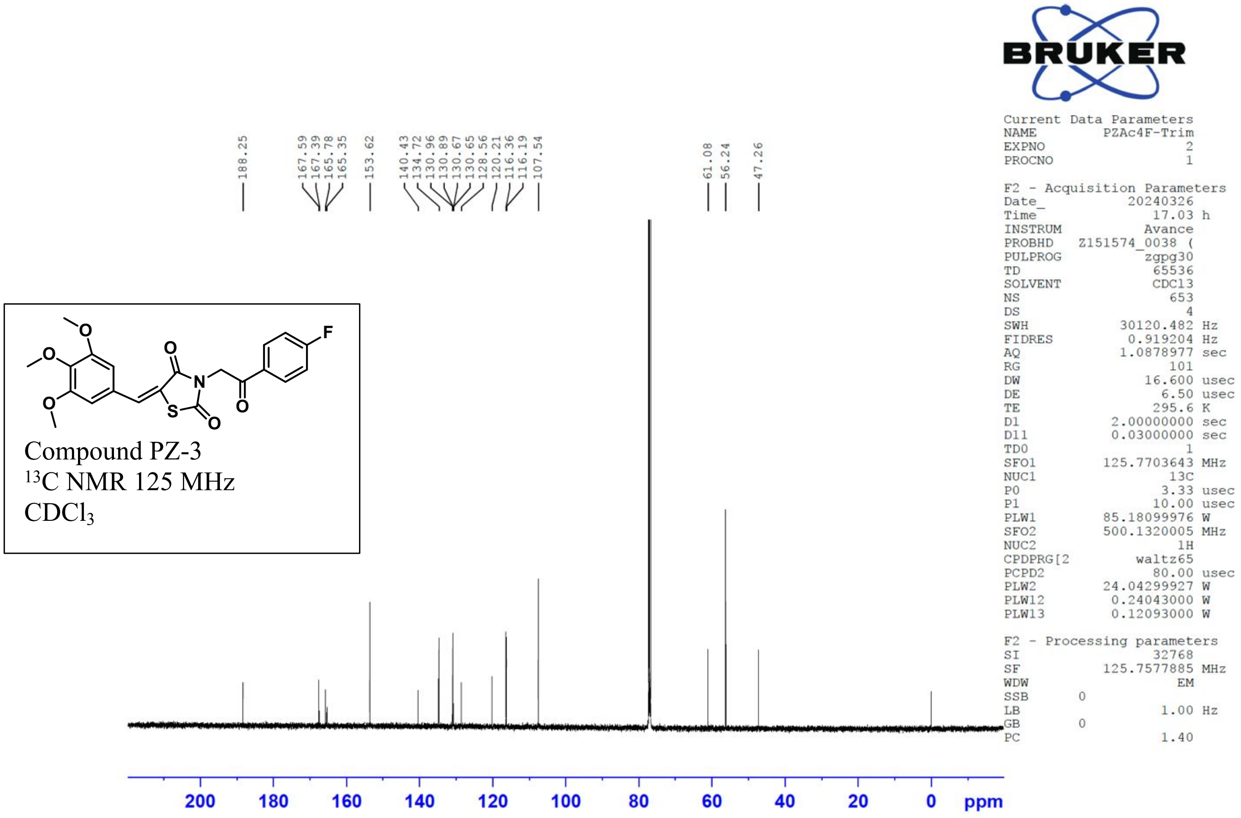

Figure S8. ^{13}C NMR spectrum of compound PZ-3

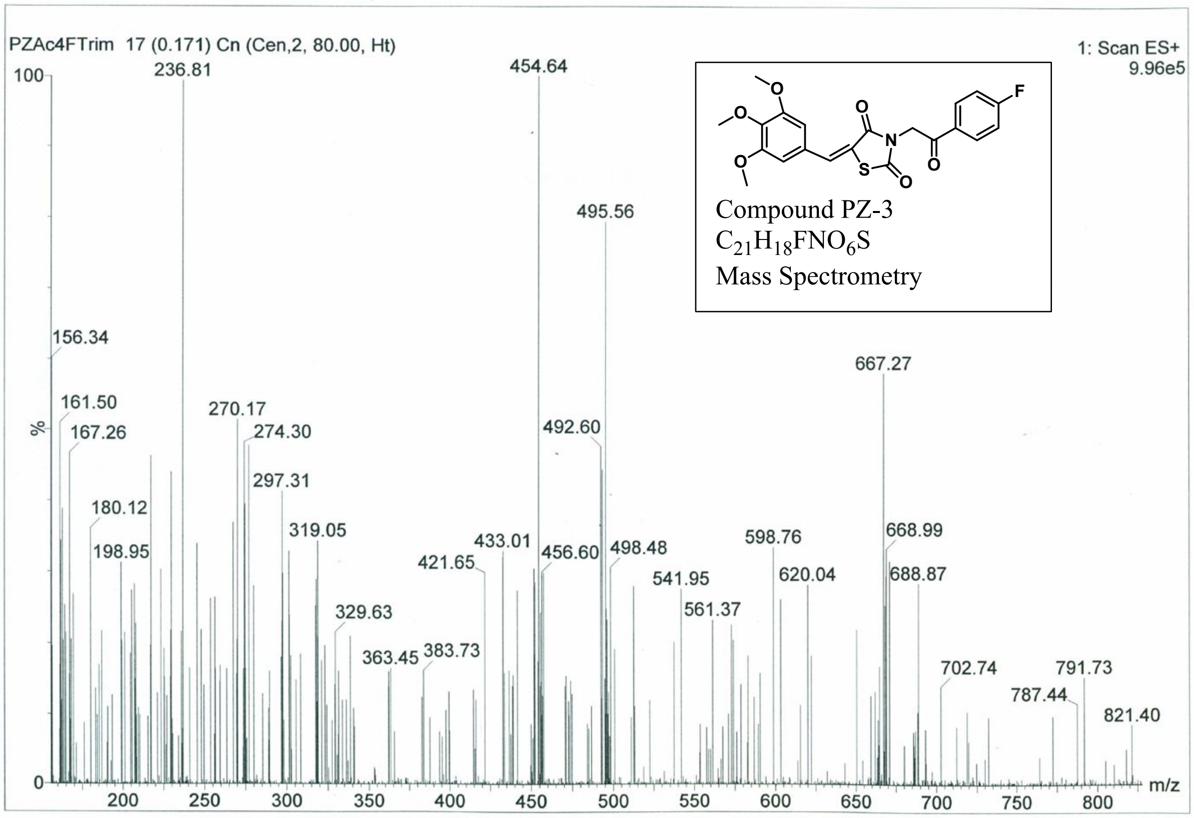

Figure S9. ESI-MS spectrum of compound PZ-3

(E)-3-(2-(3-nitrophenyl)-2-oxoethyl)-5-(3,4,5-trimethoxybenzylidene)thiazolidine-2,4-dione (PZ-4)

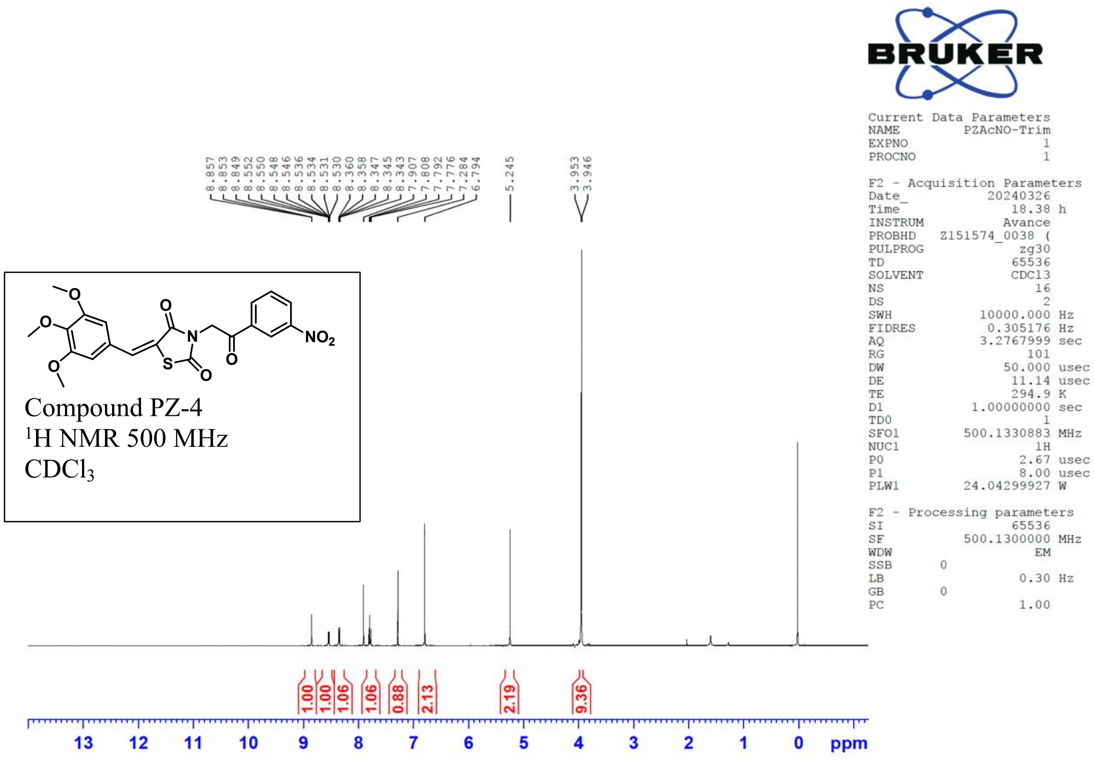

Figure S20. ^{1}H NMR spectrum of compound PZ-4

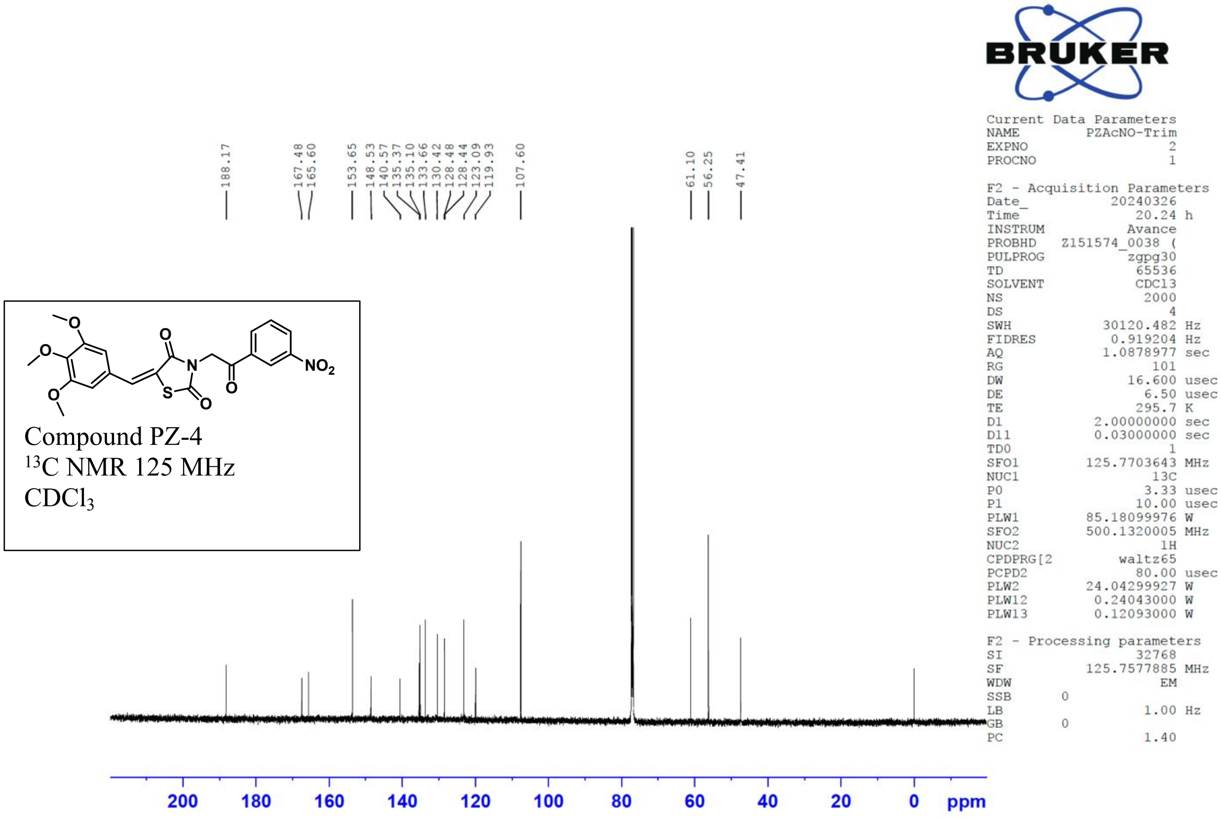

Figure S11. ^{13}C NMR spectrum of compound PZ-4

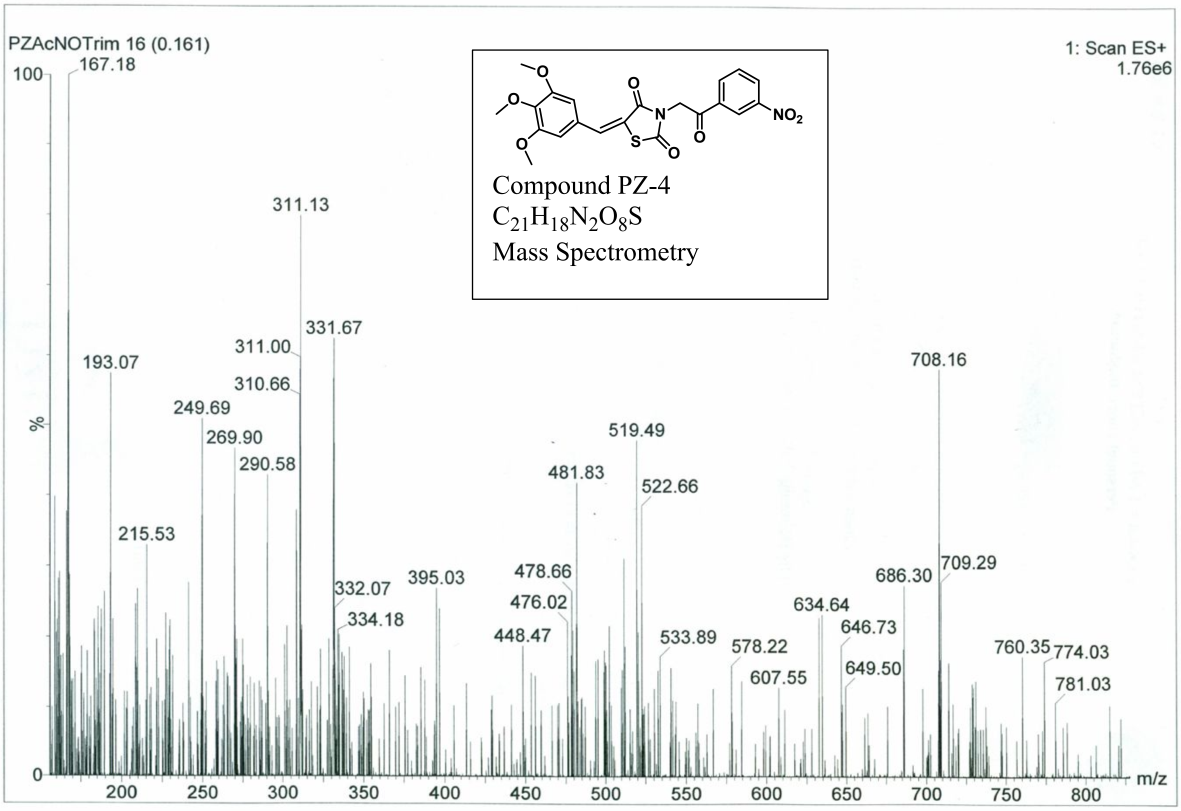

Figure S12. ESI-MS spectrum of compound PZ-4

(E)-3-(2-(3-methoxyphenyl)-2-oxoethyl)-5-(3,4,5-trimethoxybenzylidene) thiazolidine-2,4-dione (PZ-5)

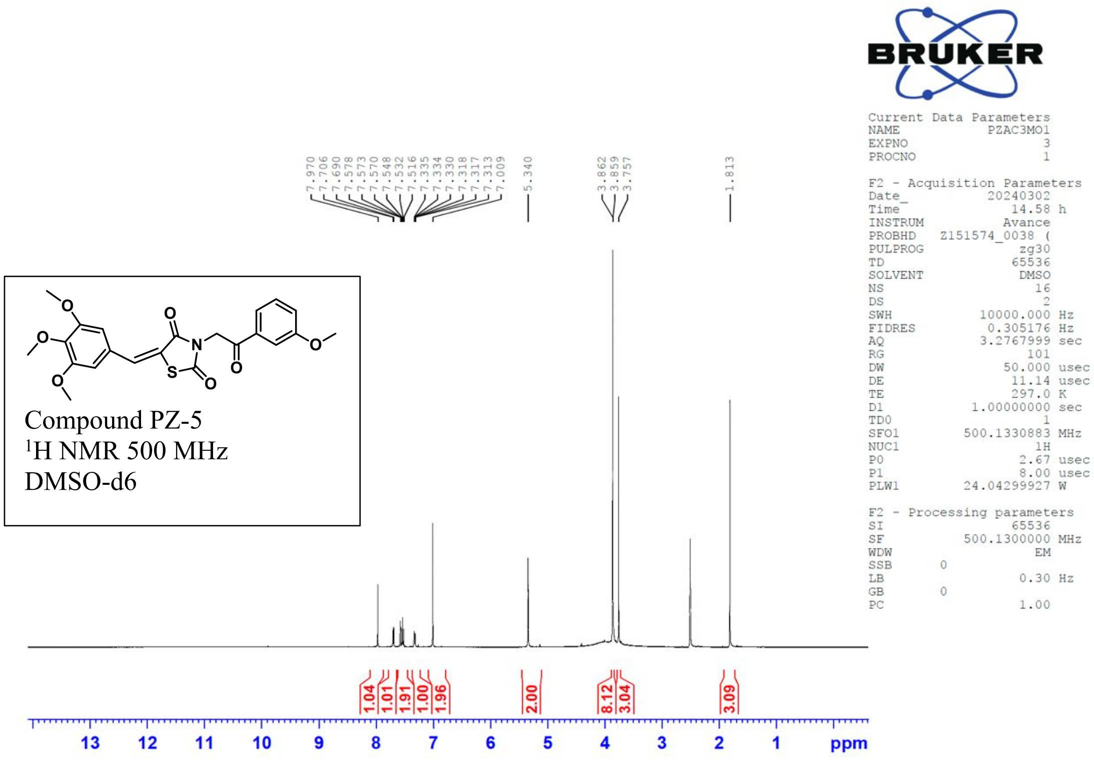

Figure S33. ^{1}H NMR spectrum of compound PZ-5

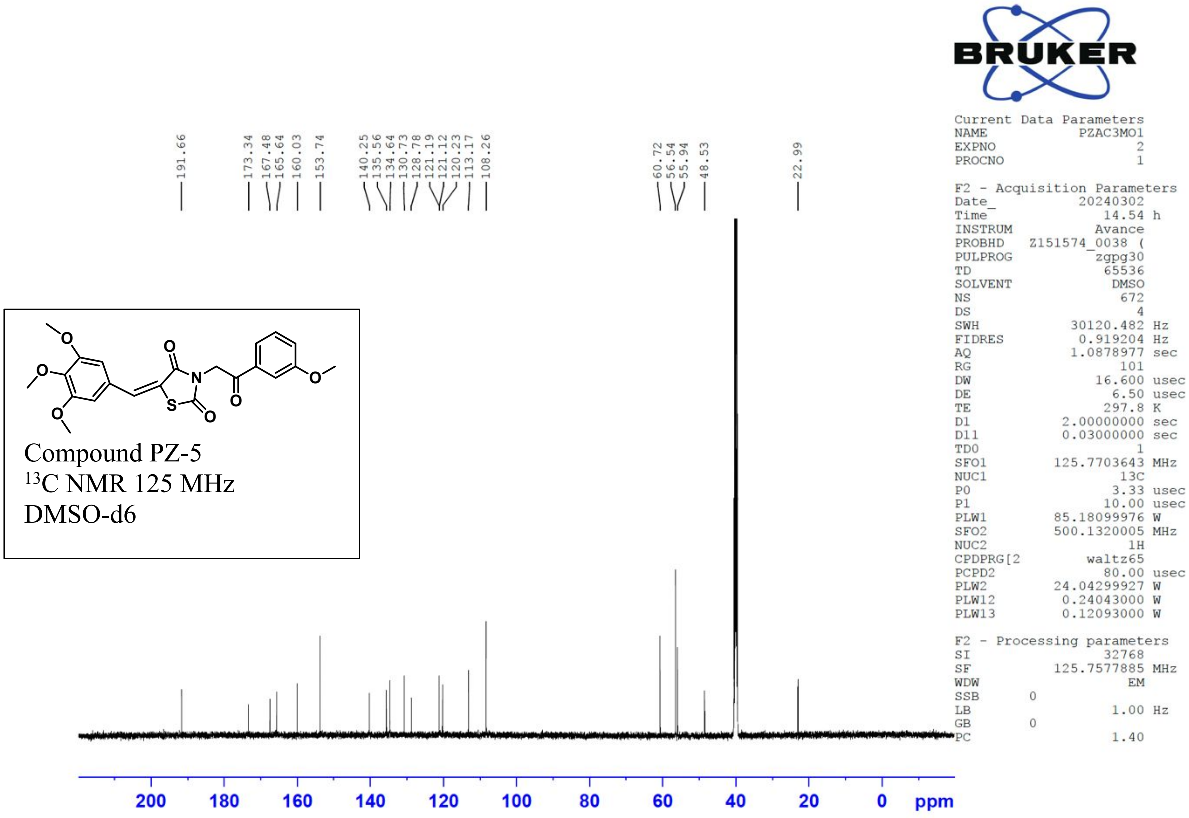

Figure S14. ^{13}C NMR spectrum of compound PZ-5

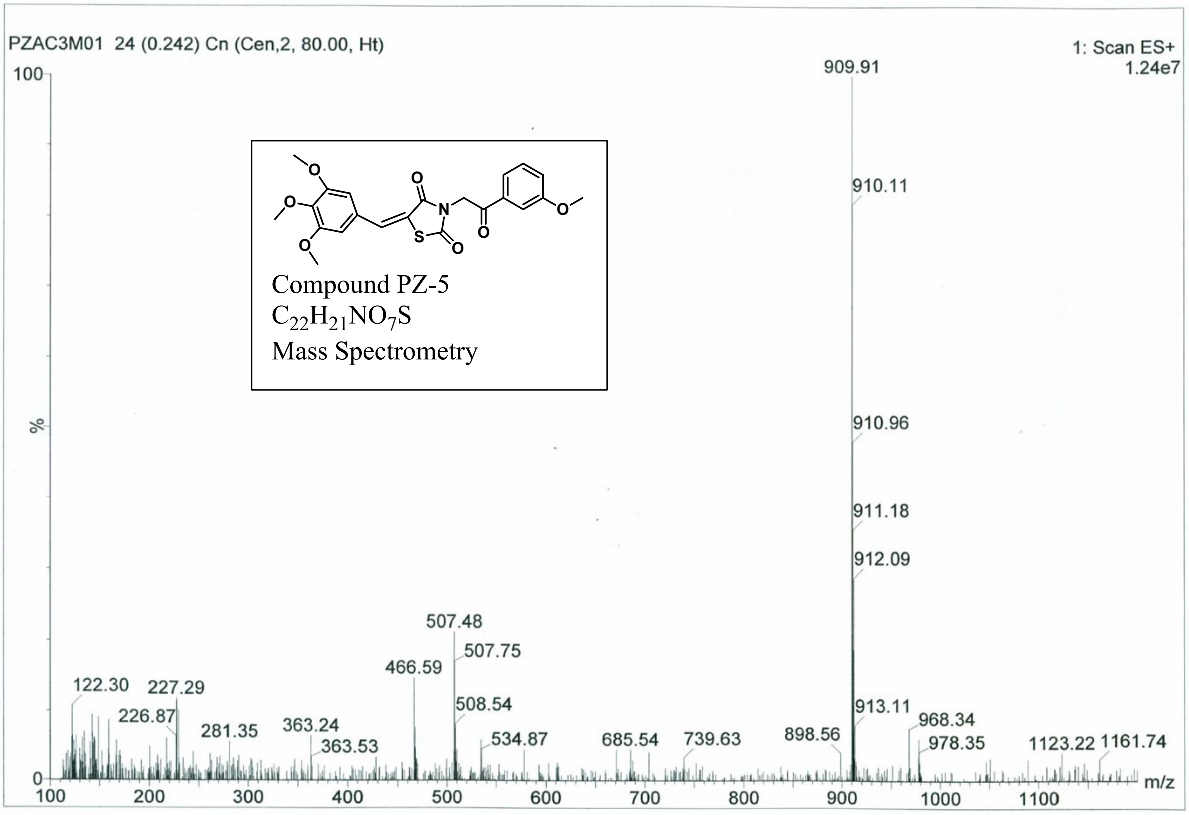

Figure S15. ESI-MS spectrum of compound PZ-5

(E)-3-(2-(4-bromophenyl)-2-oxoethyl)-5-(3,4,5-trimethoxybenzylidene)thiazolidine-2,4-dione (PZ-6)

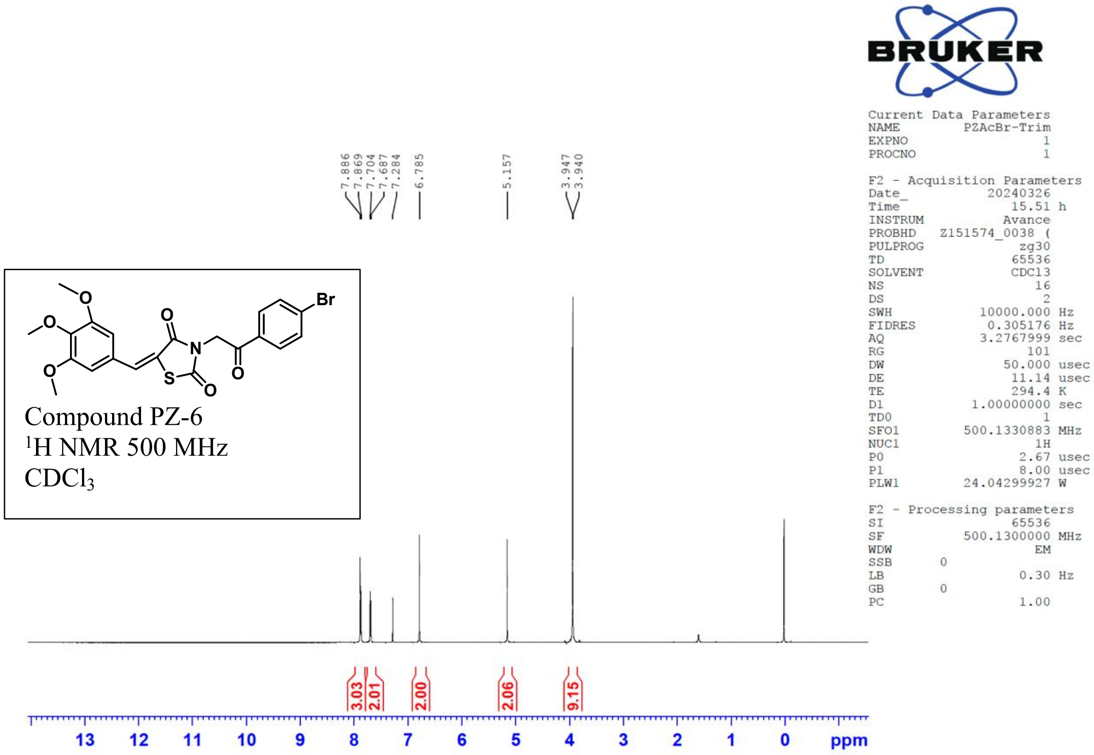

Figure S16. ^{1}H NMR spectrum of compound PZ-6

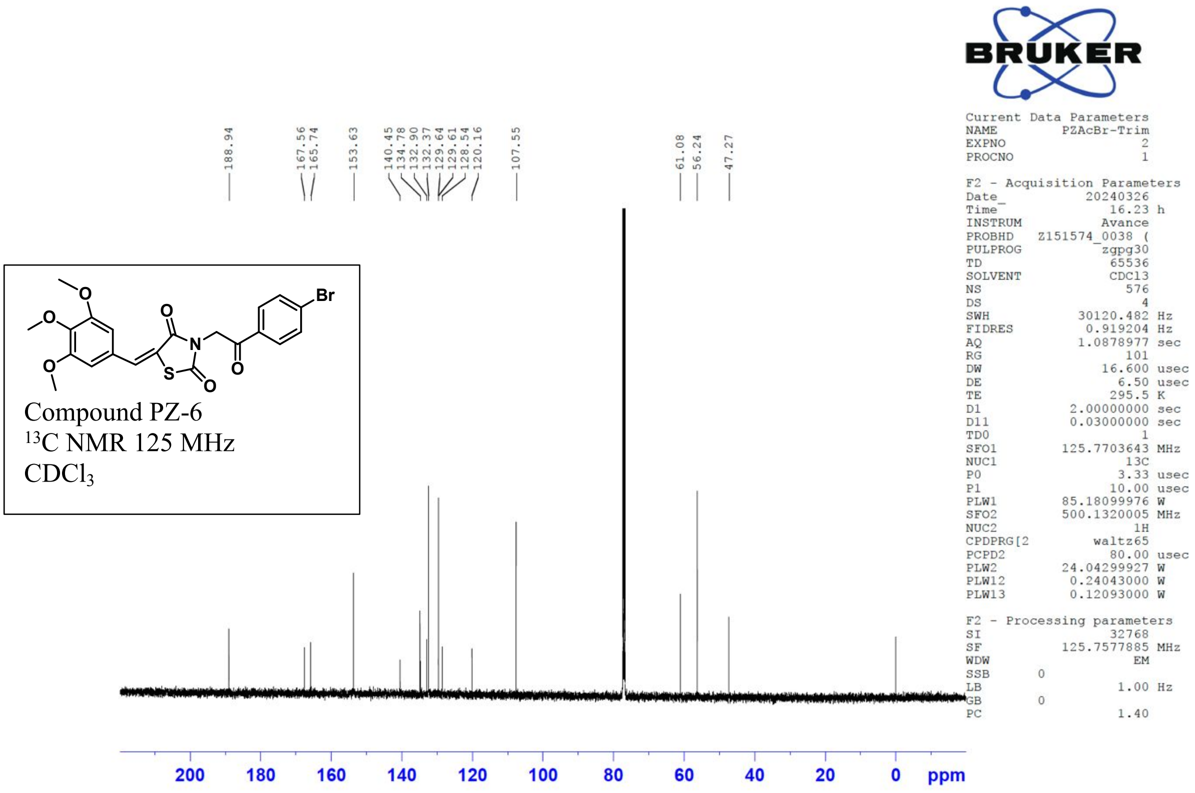

Figure S17. ^{13}C NMR spectrum of compound PZ-6

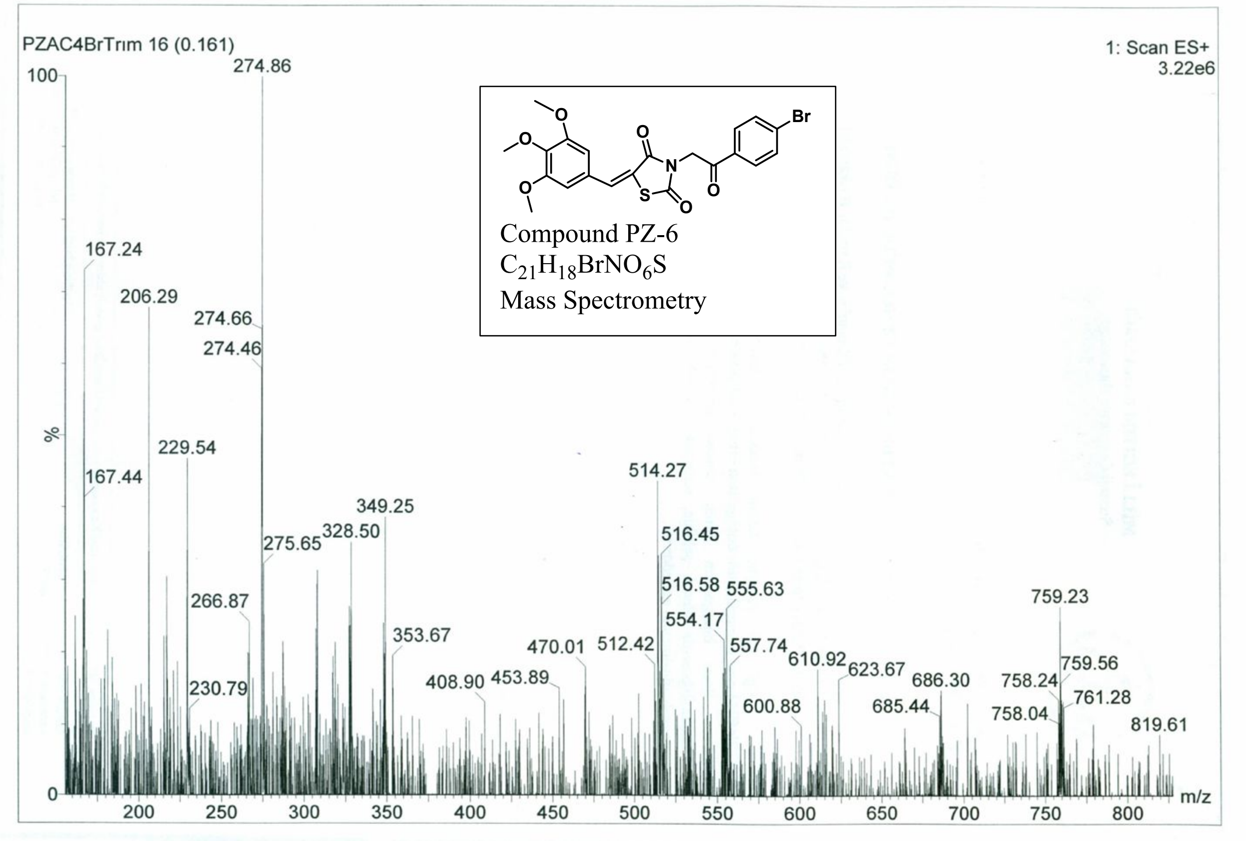

Figure S18. ESI-MS spectrum of compound PZ-6

(E)-3-(2-(3,4-dichlorophenyl)-2-oxoethyl)-5-(3,4,5-trimethoxybenzylidene) thiazolidine-2,4-dione dione (PZ-7)

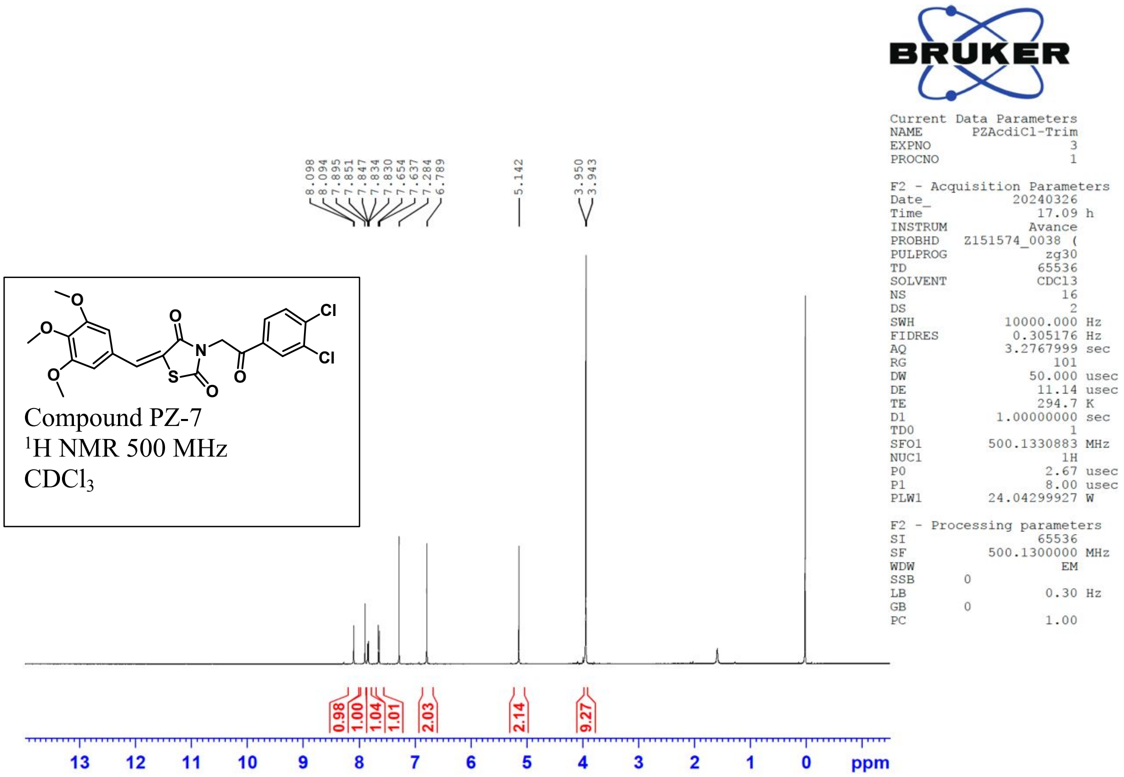

Figure S49. ^{1}H NMR spectrum of compound PZ-7

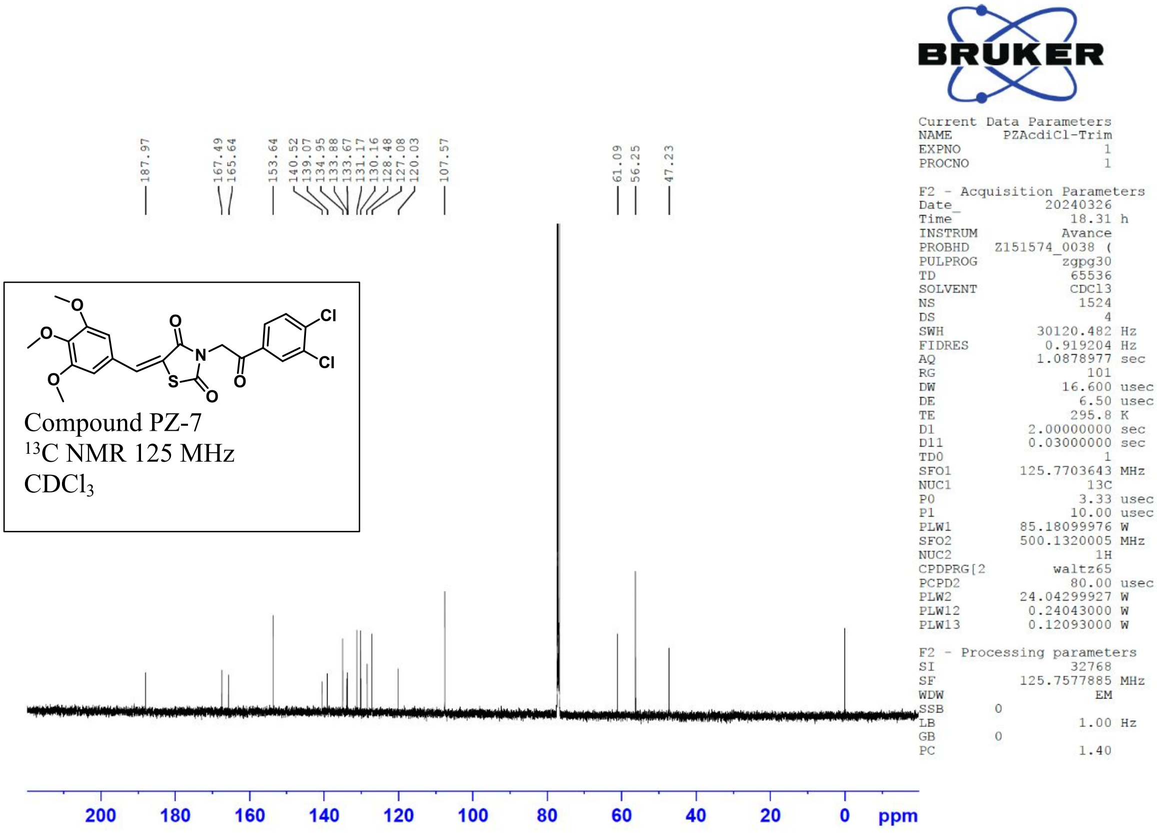

Figure S20. ^{13}C NMR spectrum of compound PZ-7

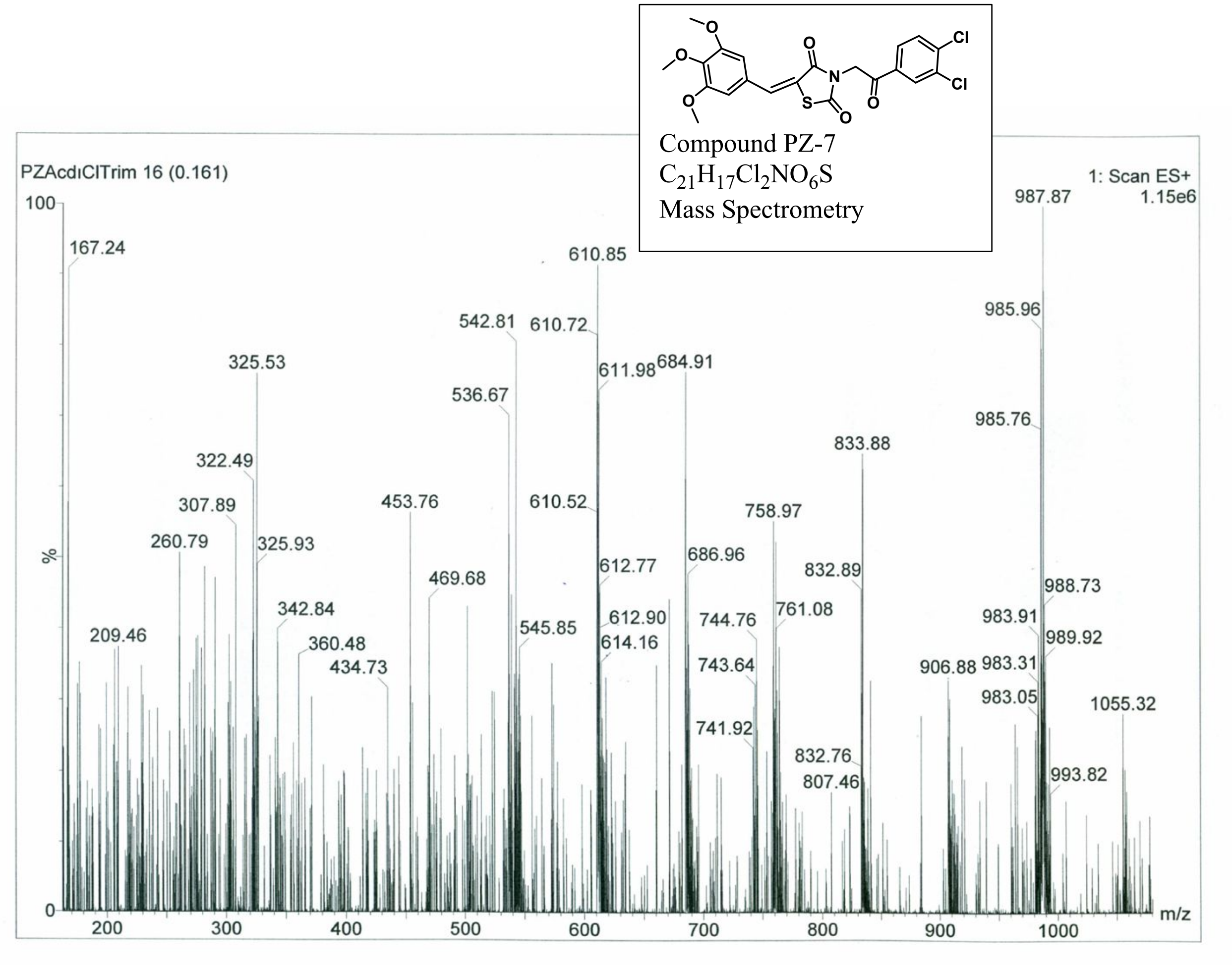

Figure S21. ESI-MS spectrum of compound PZ-7

(E)-5-(4-chlorobenzylidene)-3-(2-(4-fluorophenyl)-2-oxoethyl)thiazolidine-2,4-dione (PZ8) (PZ-8)

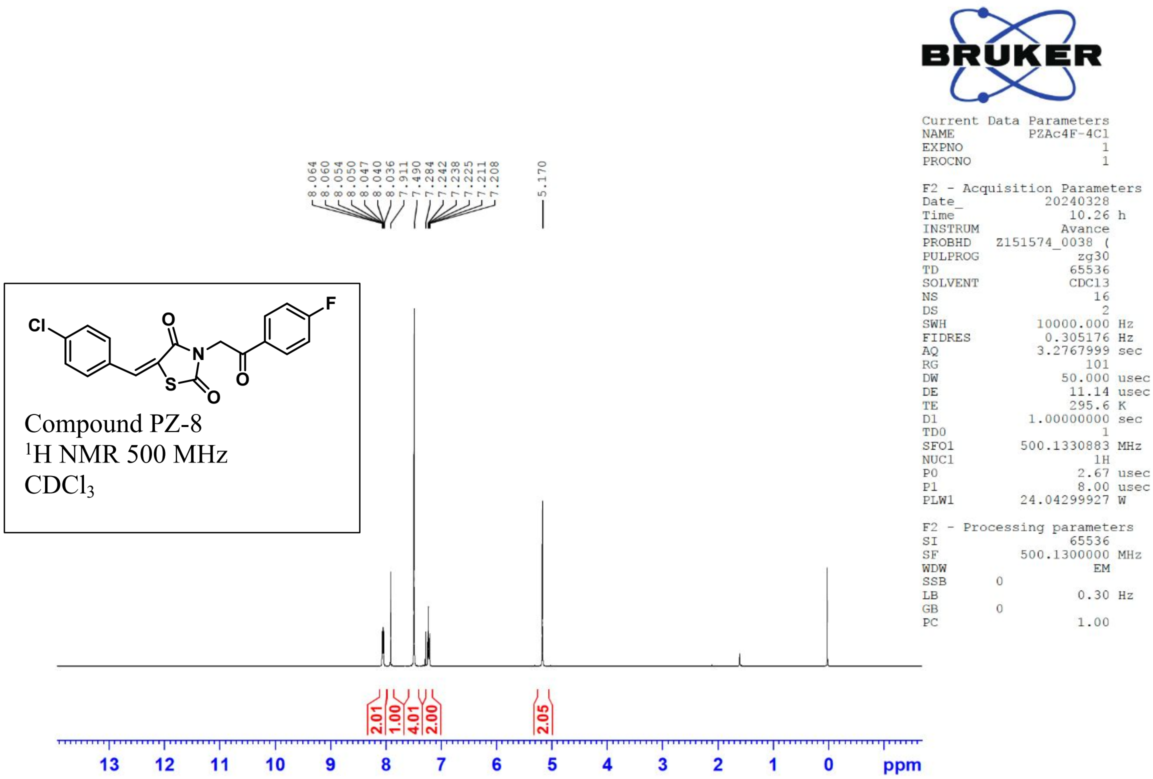

Figure S22. ^{1}H NMR spectrum of compound PZ-8

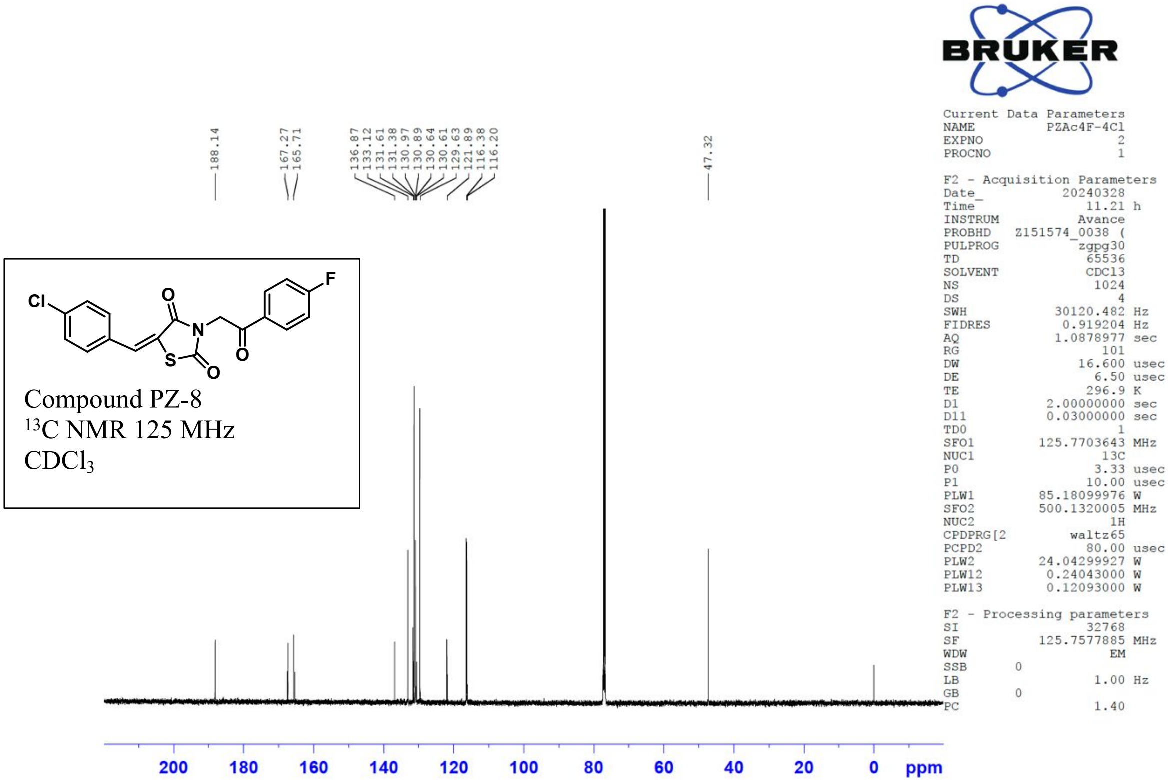

Figure S23. ^{13}C NMR spectrum of compound PZ-8

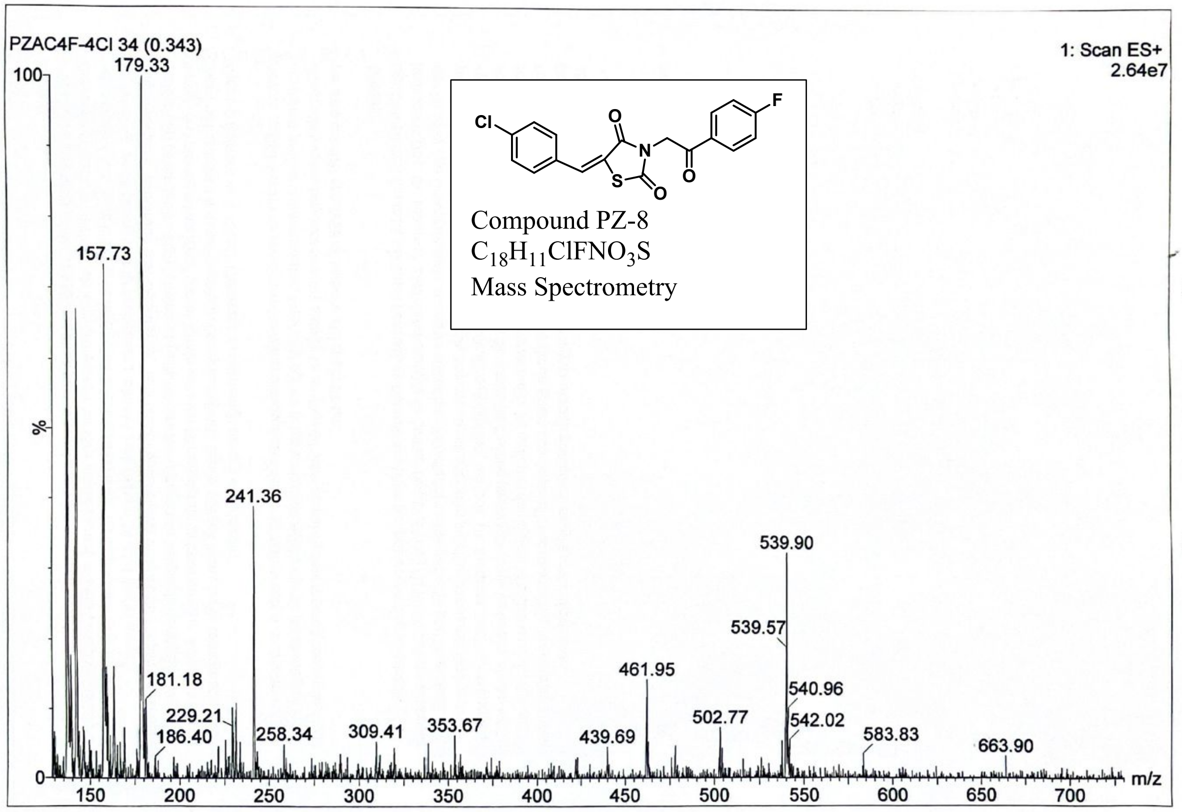

Figure S24. ESI-MS spectrum of compound PZ-8

(E)-5-(4-chlorobenzylidene)-3-(2-(3-methoxyphenyl)-2-oxoethyl)thiazolidine-2,4-dione (PZ-9)

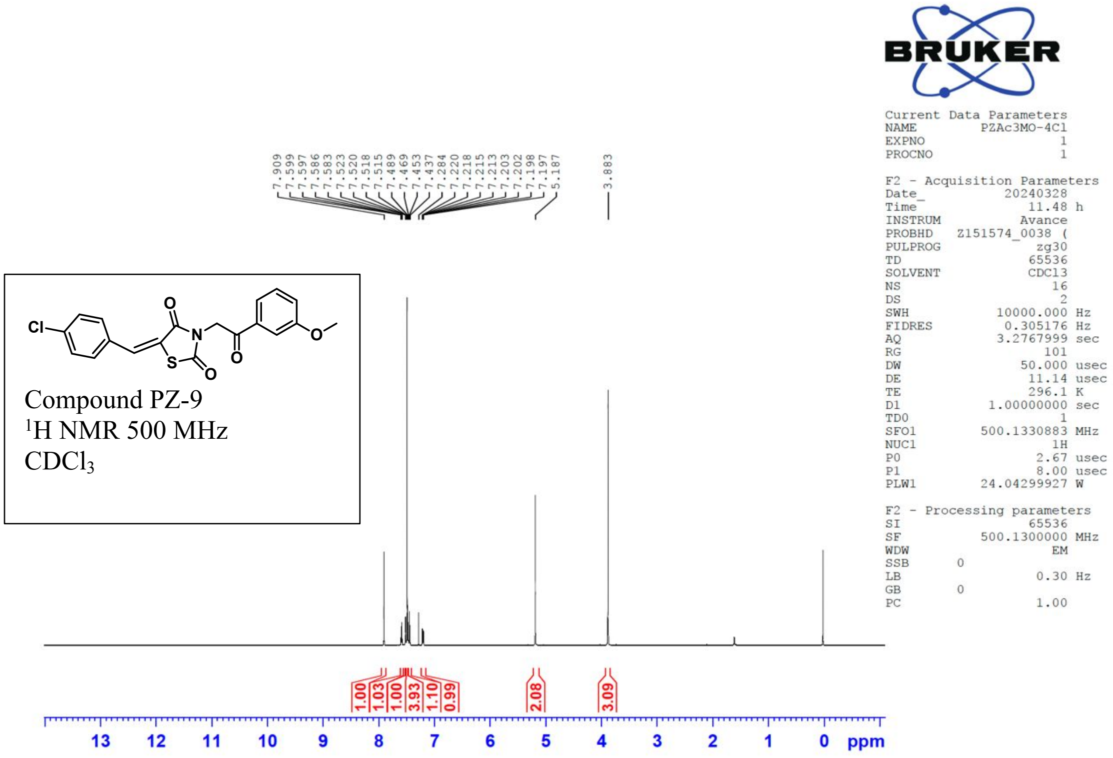

Figure S25. ^{1}H NMR spectrum of compound PZ-9

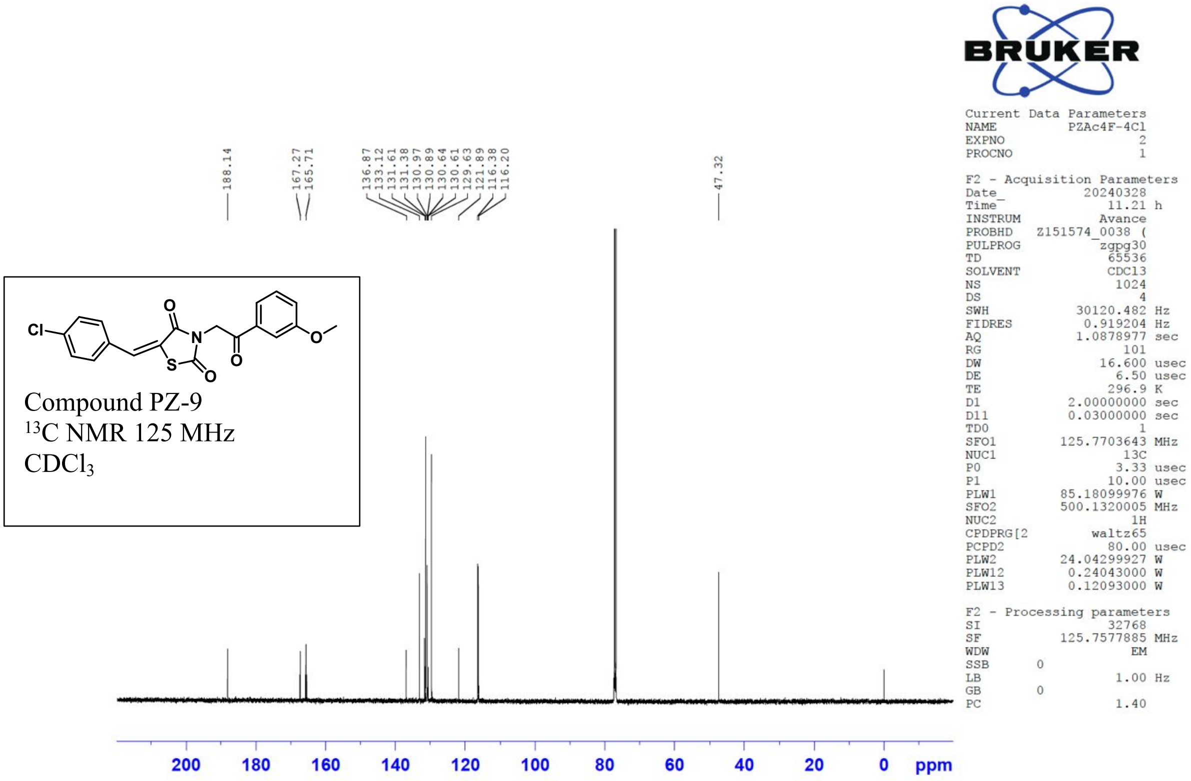

Figure S26. ^{13}C NMR spectrum of compound PZ-9

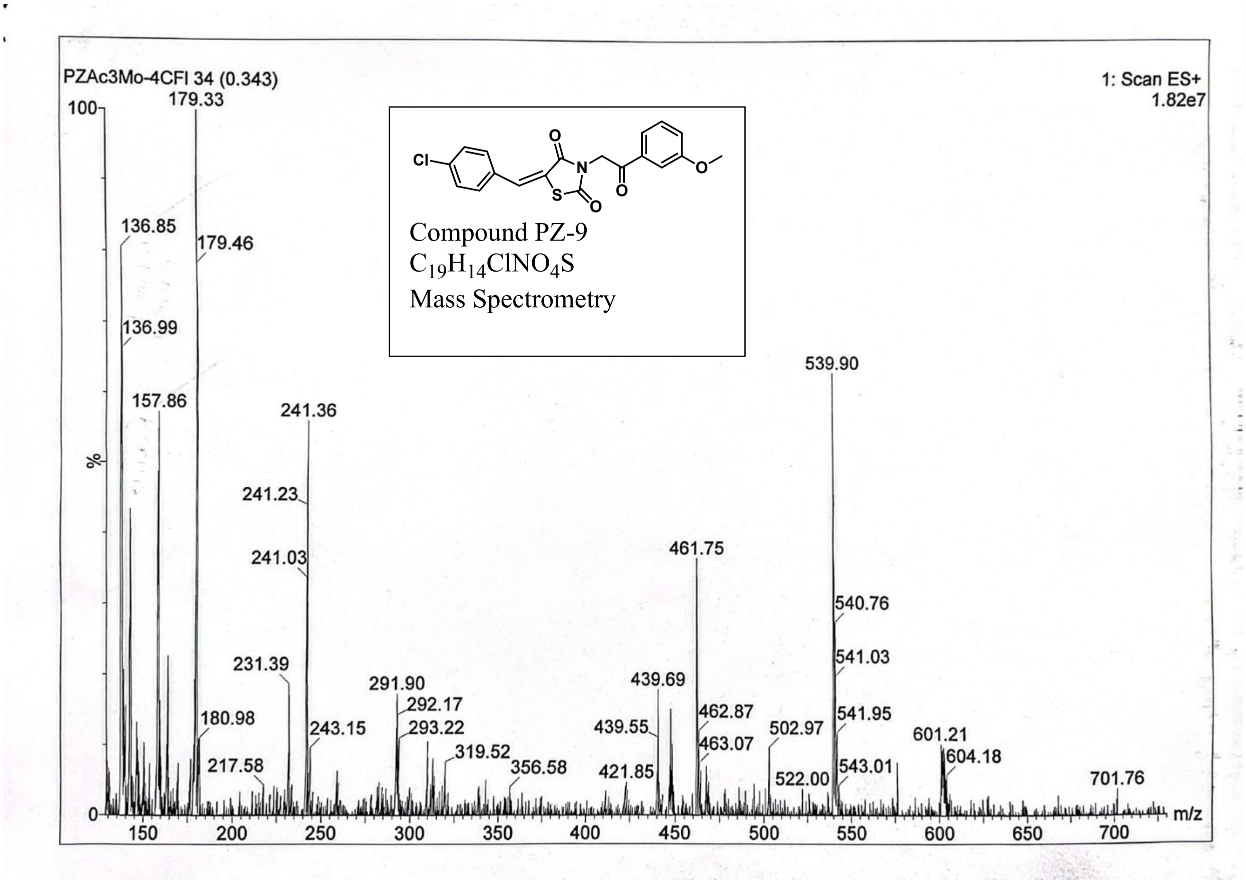

Figure S27. ESI-MS spectrum of compound PZ-9

(E)-5-(4-chlorobenzylidene)-3-(2-(3,4-dichlorophenyl)-2-oxoethyl)thiazolidine-2,4-dione (PZ-10)

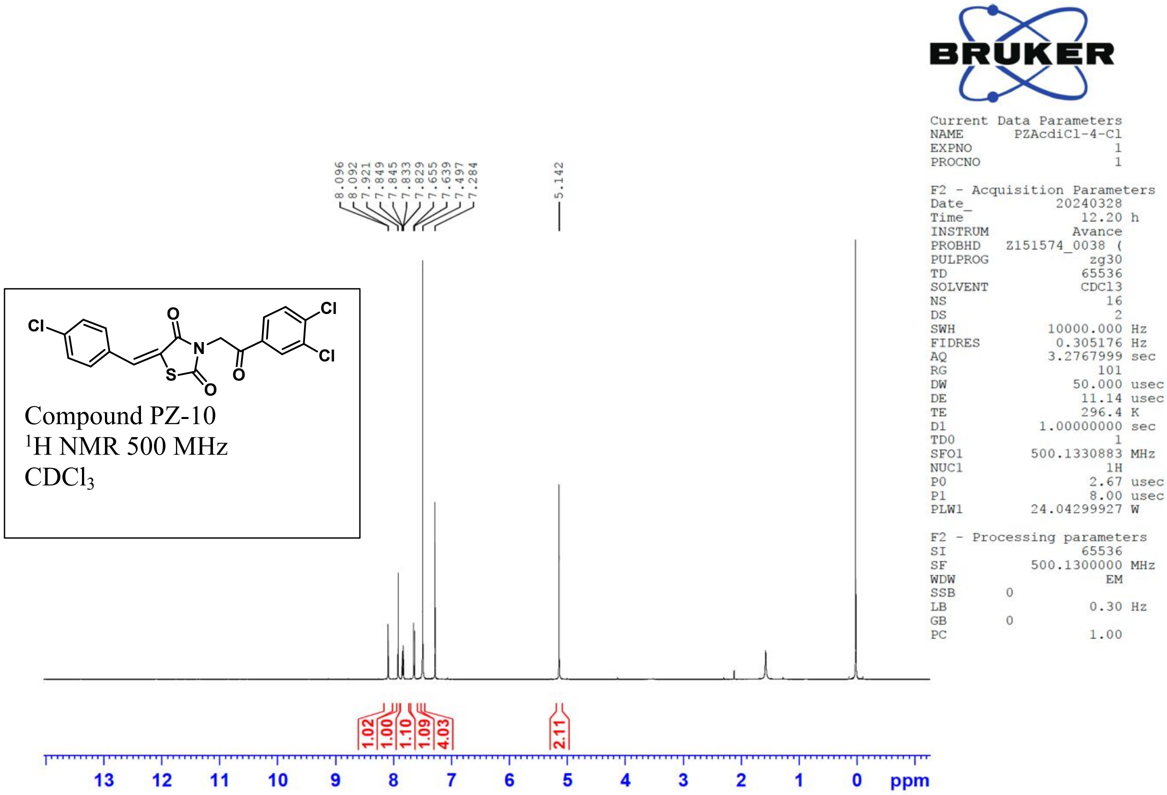

Figure S28. ^{1}H NMR spectrum of compound PZ-10

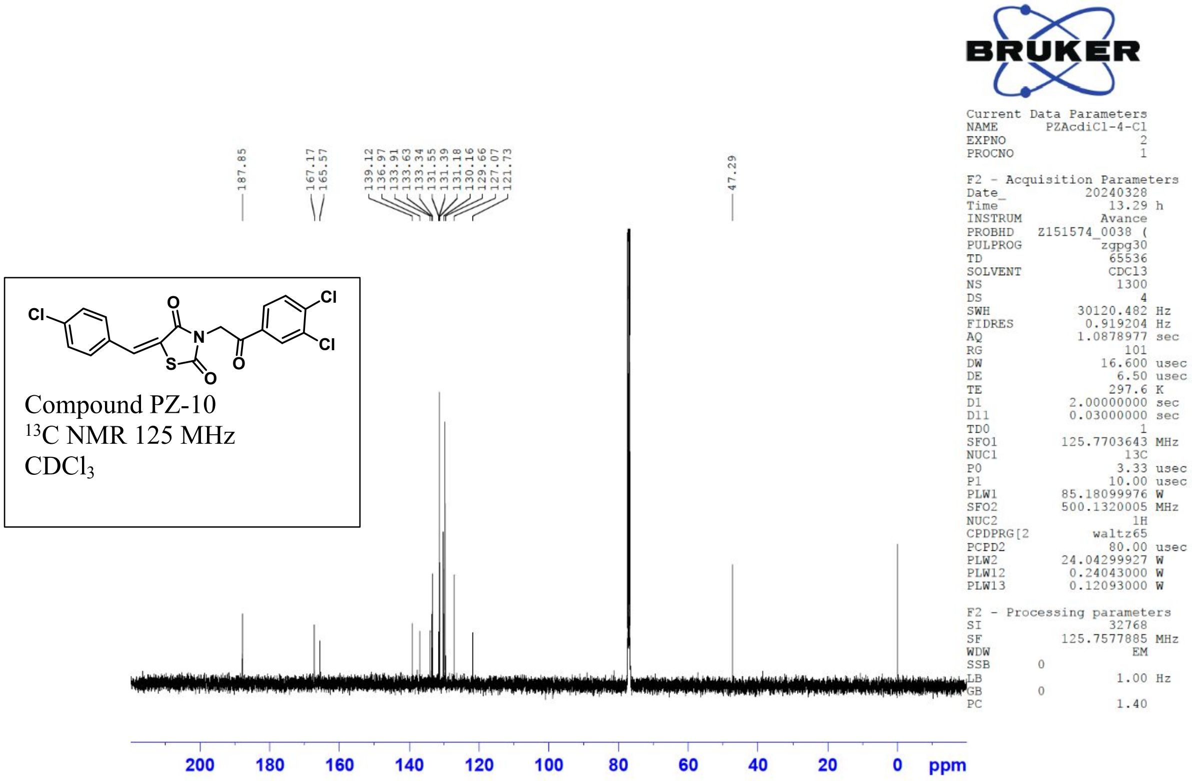

Figure S29. ^{13}C NMR spectrum of compound PZ-10

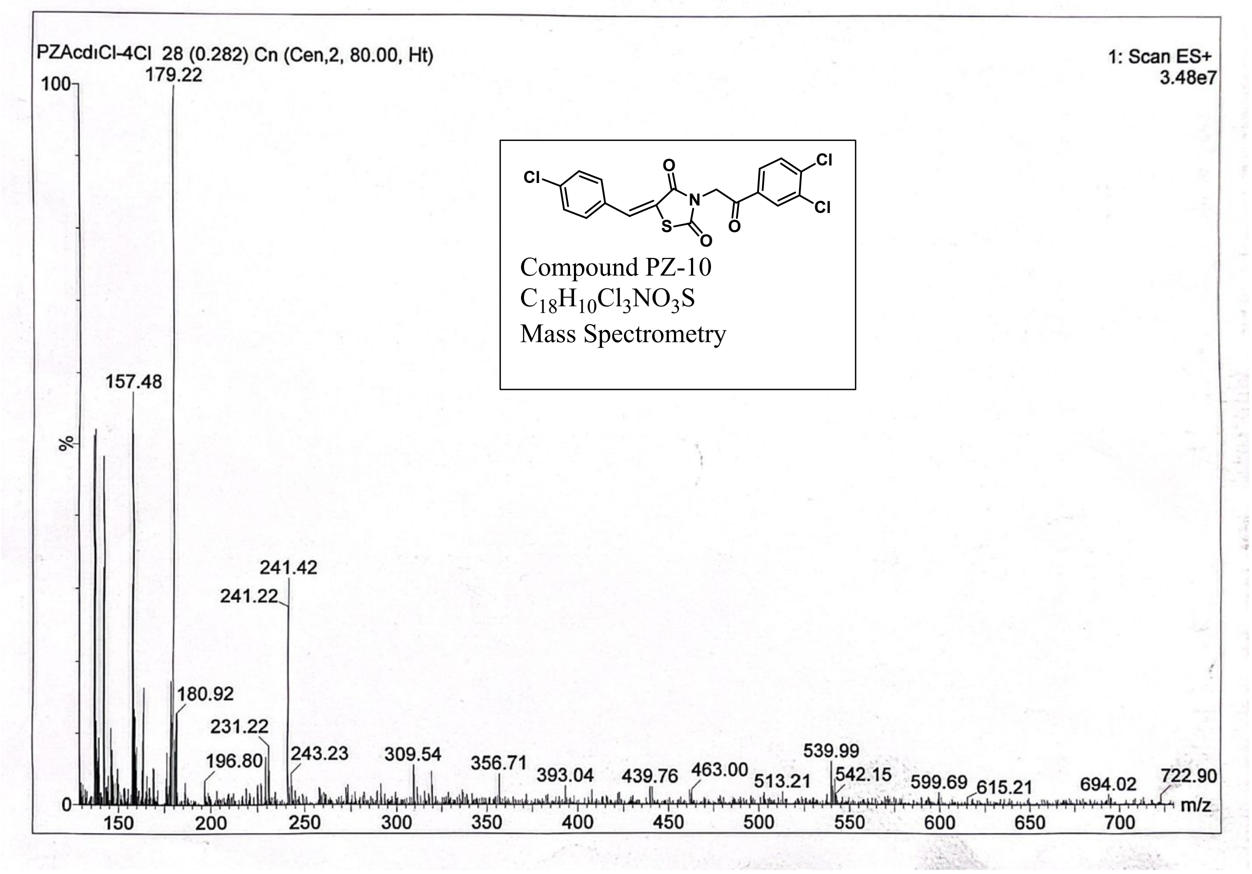

Figure S30. ESI-MS spectrum of compound PZ-10

(E)-5-(4-chlorobenzylidene)-3-(2-(3-nitrophenyl)-2-oxoethyl)thiazolidine-2,4-dione (PZ-11)

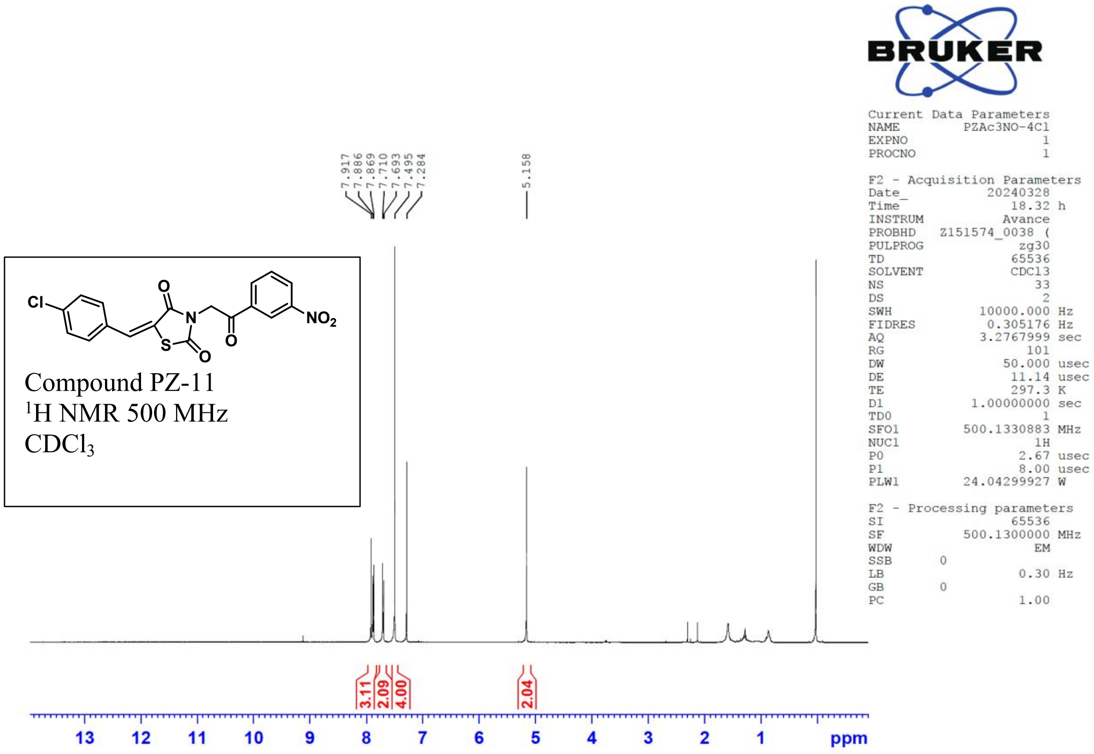

Figure S35. ^{1}H NMR spectrum of compound PZ-11

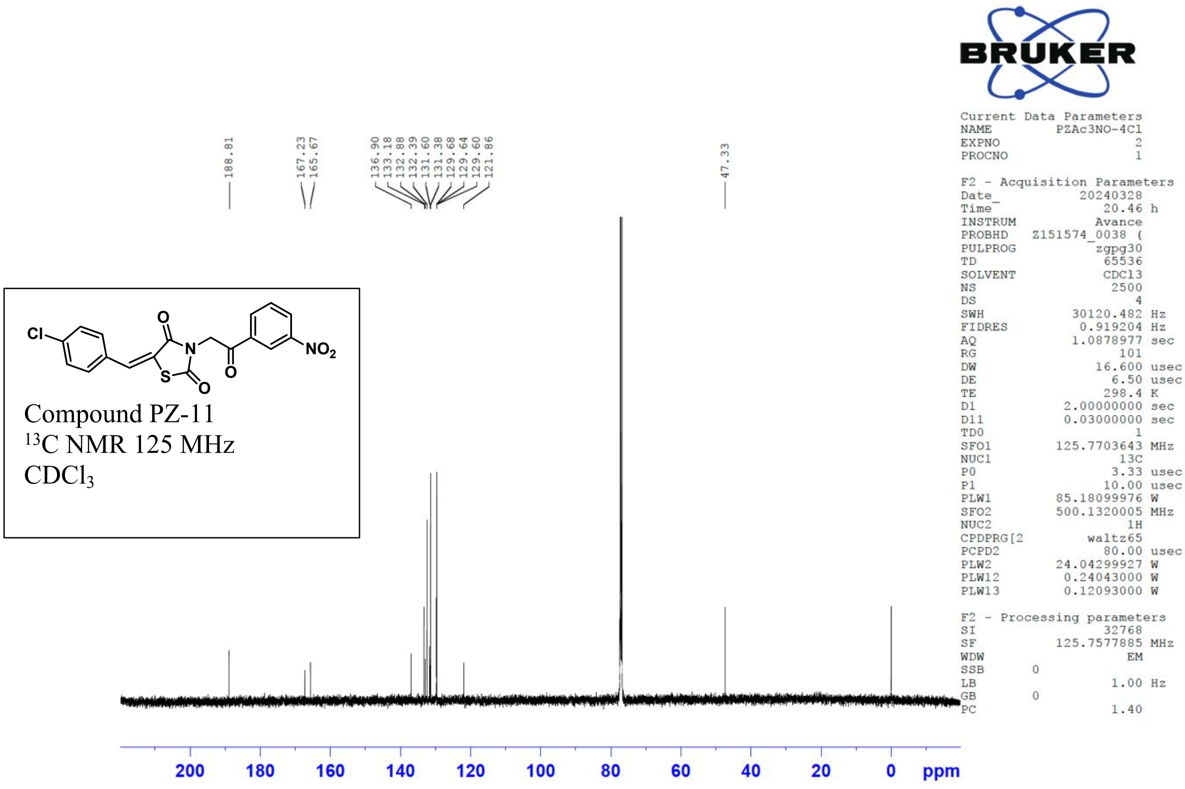

Figure S32. ^{13}C NMR spectrum of compound PZ-11

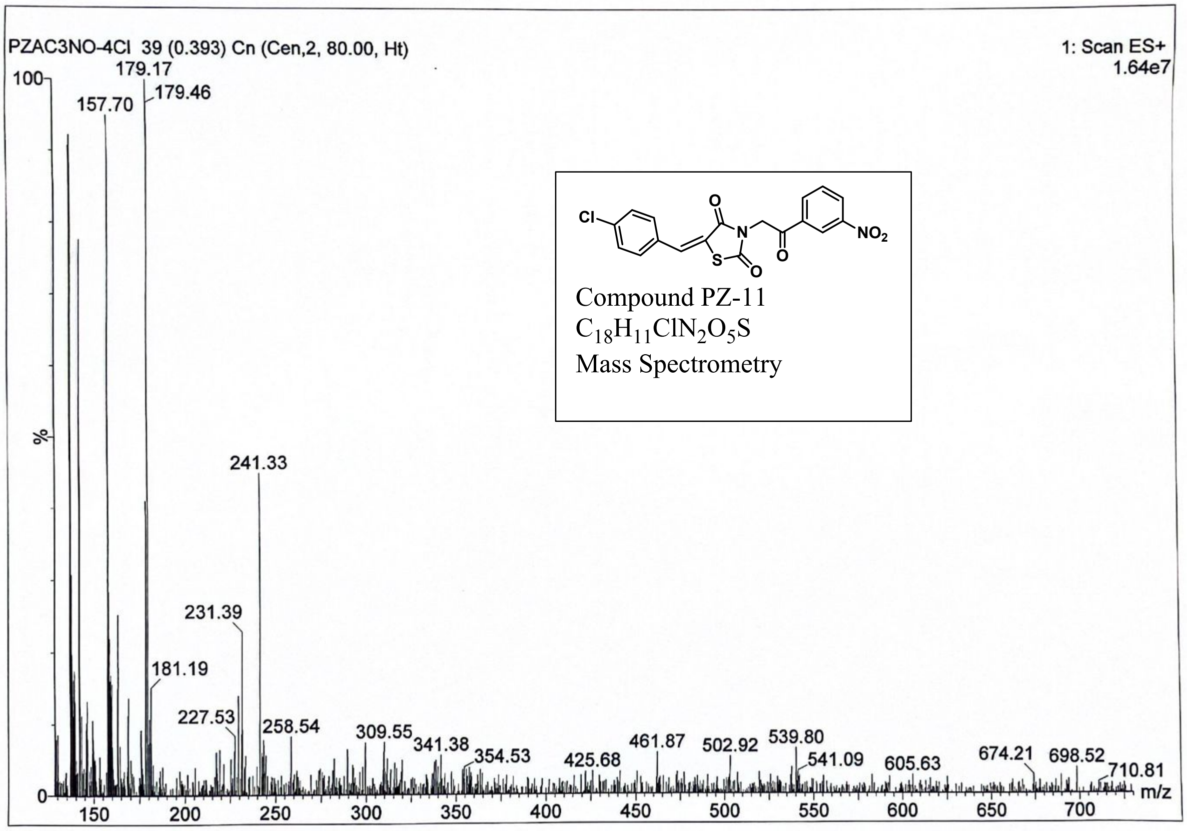

Figure S33. ESI-MS spectrum of compound PZ-11

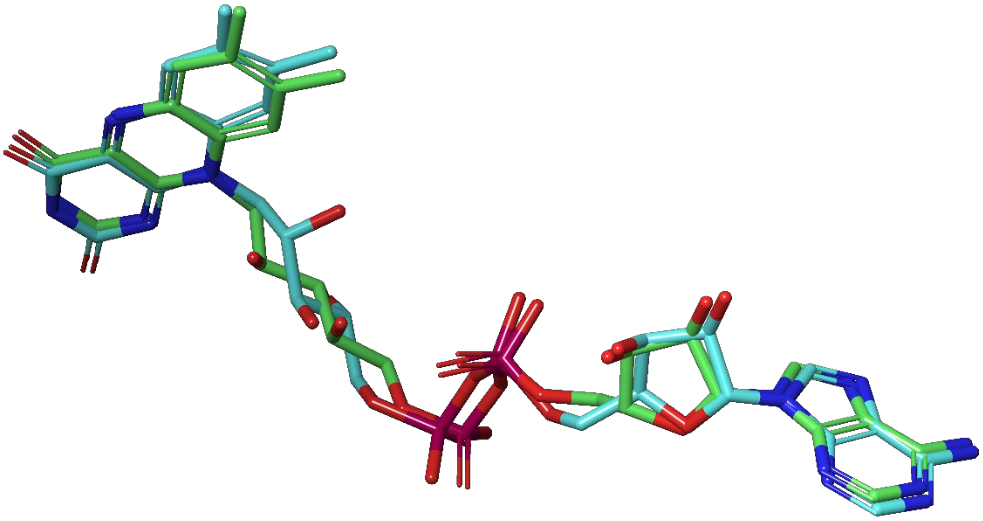

Figure S34. Alignment of AIF co-ligand FAD (Cyan color) and its re-docked conformer (Green). Root mean square deviation (RMSD) between the natural pose and docked poses is 0.93 Å.
